# Somatic copy number alteration and fragmentation analysis in circulating tumor DNA for cancer screening and treatment monitoring in colorectal cancer patients

**DOI:** 10.1186/s13045-022-01342-z

**Published:** 2022-09-02

**Authors:** Ariane Hallermayr, Tobias Wohlfrom, Verena Steinke-Lange, Anna Benet-Pagès, Florentine Scharf, Ellen Heitzer, Ulrich Mansmann, Christopher Haberl, Maike de Wit, Holger Vogelsang, Markus Rentsch, Elke Holinski-Feder, Julia M. A. Pickl

**Affiliations:** 1 MGZ – Medizinisch Genetisches Zentrum, Munich, Germany; 2 Pettenkofer School of Public Health, Munich, Germany; 3 Institute for Medical Information Processing, Biometry, and Epidemiology –IBE, LMU Munich, Munich, Germany; 4 Medizinische Klinik Und Poliklinik IV, Campus Innenstadt, Klinikum Der Universität München, Munich, Germany; 5 Institute of Neurogenomics, Helmholtz Zentrum München - German Research Center for Environmental Health, Neuherberg, Germany; 6 Institute of Human Genetics, Diagnostic and Research Center for Molecular Biomedicine (Austria), Medical University of Graz, Graz, Austria; 7 BioTechMed-Graz, Graz, Austria; 8 Christian Doppler Laboratory for Liquid Biopsies for Early Detection of Cancer, Graz, Austria; 9 Department of Oncology and Hematology, Barmherzige Brüder, Klinikum St. Elisabeth, Straubing, Germany; 10 Department of Hematology, Oncology and Palliative Medicine, Vivantes Klinikum Neukoelln, Berlin, Germany; 11 Department of Oncology, Vivantes Auguste-Viktoria-Klinikum, Berlin, Germany; 12 Department of General, Visceral, Thoracic and Endocrine Surgery, Klinikum Garmisch-Partenkirchen, Teaching Hospital, Ludwig Maximilian University Munich, Garmisch-Partenkirchen, Germany; 13 Department of General, Visceral and Thorax Surgery, Klinikum Ingolstadt, Ingolstadt, Germany; 14 Department of General, Visceral, Vascular and Transplant Surgery, University Hospital Munich, Ludwig-Maximilians University of Munich, Campus Großhadern, Munich, Germany

**Keywords:** ctDNA, Colorectal cancer, Liquid biopsy, Whole-genome sequencing, Somatic copy number alterations, cfDNA fragmentation, Chromatin signatures

## Abstract

**Background:**

Analysis of circulating free DNA (cfDNA) is a promising tool for personalized management of colorectal cancer (CRC) patients. Untargeted cfDNA analysis using whole-genome sequencing (WGS) does not need a priori knowledge of the patient´s mutation profile.

**Methods:**

Here we established LIquid biopsy Fragmentation, Epigenetic signature and Copy Number Alteration analysis (LIFE-CNA) using WGS with ~ 6× coverage for detection of circulating tumor DNA (ctDNA) in CRC patients as a marker for CRC detection and monitoring.

**Results:**

We describe the analytical validity and a clinical proof-of-concept of LIFE-CNA using a total of 259 plasma samples collected from 50 patients with stage I-IV CRC and 61 healthy controls. To reliably distinguish CRC patients from healthy controls, we determined cutoffs for the detection of ctDNA based on global and regional cfDNA fragmentation patterns, transcriptionally active chromatin sites, and somatic copy number alterations. We further combined global and regional fragmentation pattern into a machine learning (ML) classifier to accurately predict ctDNA for cancer detection. By following individual patients throughout their course of disease, we show that LIFE-CNA enables the reliable prediction of response or resistance to treatment up to 3.5 months before commonly used CEA.

**Conclusion:**

In summary, we developed and validated a sensitive and cost-effective method for untargeted ctDNA detection at diagnosis as well as for treatment monitoring of all CRC patients based on genetic as well as non-genetic tumor-specific cfDNA features. Thus, once sensitivity and specificity have been externally validated, LIFE-CNA has the potential to be implemented into clinical practice. To the best of our knowledge, this is the first study to consider multiple genetic and non-genetic cfDNA features in combination with ML classifiers and to evaluate their potential in both cancer detection and treatment monitoring.

*Trial registration* DRKS00012890.

**Supplementary Information:**

The online version contains supplementary material available at 10.1186/s13045-022-01342-z.

## Background

Liquid biopsy (LB) is a highly promising tool for personalized patient management [1–5]. An important LB marker is circulating tumor DNA (ctDNA), which represents the fraction of circulating free DNA (cfDNA) released by tumor cells [6]. A major challenge in ctDNA analysis is the very low fractions of ctDNA in total cfDNA (commonly < 5%) [6, 7]. Therefore, methods with high analytical sensitivity and specificity are required [8–10], but to date, mainly methods targeting frequent hotspot variants have been validated [11–14]. However, this approach limits the application of LB to patients with known genetic tumor profiles. To extend the advantages of LB to all cancer patients, highly sensitive untargeted methods are required.

A commonly used approach for untargeted ctDNA detection is shallow whole-genome sequencing (WGS) (i.e., < 1× coverage) to identify genome-wide somatic copy number alterations (SCNAs) [15] However, this approach requires ctDNA fractions of at least 5% to 10% that may be present in a subset of CRC samples only [15–17]. Various studies suggest that enrichment of the ctDNA fraction in cfDNA by size selection, tumor-specific fragmentation patterns, and epigenetic signatures can enhance ctDNA detection [18–21].

In this study, we developed LIquid biopsy Fragmentation, Epigenetic signature and Copy Number Alteration analysis (LIFE-CNA) as an untargeted approach to detect ctDNA with high sensitivity in plasma samples of colorectal cancer (CRC) patients as a diagnostic, predictive and prognostic marker. To enable detection of ctDNA fractions < 5%, we increased coverage from shallow WGS to ~ 6× and combined the Illumina DRAGEN CNV (copy number variation) workflow with the Plasma-Seq pipeline for copy number profiling [15, 22], a fragmentation pipeline, and LIQUORICE, a tool for the identification of coverage in open-chromatin regions [20]. With this workflow, we integrated detection of multiple cfDNA features, including focal SCNAs, cfDNA fragmentation patterns and chromatin signatures, and established machine learning (ML) classifiers for the highly sensitive detection of ctDNA. Using LIFE-CNA, we aimed to establish cutoffs for ctDNA detection to facilitate translation of untargeted LB analysis into clinical practice. We further evaluated whether ctDNA analysis using LIFE-CNA is able to predict response or resistance to treatment. For analytical validation and a clinical proof-of-concept of LIFE-CNA, 259 cfDNA samples from 50 patients with stage I-IV CRC and 61 healthy controls were analyzed. To the best of our knowledge, this is the first study combining SCNA and fragmentation profiles for disease monitoring and providing a complete analytically validated workflow showing a clinical proof-of-concept that can be easily implemented into clinical practice to support CRC patient management.

## Methods

### Study design and participants

A total of 259 plasma samples were collected from 50 patients with UICC stage I-IV CRC (7 stage I, 14 stage II, 11 stage III, 18 stage IV) and 61 healthy individuals aged 20 to 88 years from March 2018 until April 2022 (Additional file 1: Table S1, Figure S1; Additional file 2: Table S2) [23]. 55 healthy controls were included in the reference set. Six healthy controls were used for external validation of LIFE-CNA. 198 plasma samples from 50 CRC patients were collected at diagnosis and during follow-up. These samples were categorized according to the time of sample collection during the course of disease (Additional file 1: Methods, Table S1). The course of disease was monitored by colonoscopies and imaging during routinely scheduled follow-up examinations. 134 of the 198 plasma samples from CRC patients collected during the course of disease with clinically diagnosed tumor burden served as positive controls. To identify molecular residual disease (MRD) following surgery, baseline blood samples were collected up to eight days pre-surgery and follow-up samples were collected one day up to six weeks post-surgery in 33 patients. For treatment monitoring, plasma samples from 15 patients were collected at several time points throughout the course of disease.

The study was approved by the ethics commission of the Bavarian Medical Association (No. 17059) and is registered with the German registry for clinical trials (trial registration ID: DRKS00012890). Neither clinicians nor patients were informed about the results. All participants provided informed written consent prior to blood and tissue specimen collection.

### Clinical sample collection and categorization, DNA extraction, droplet digital PCR, CEA analysis, library preparation and in silico dilutions

Information on sample collection and categorization, DNA extraction, droplet digital PCR (ddPCR), carcinoembryonic antigen (CEA) analysis, library preparation, and in silico dilutions are provided in the Supplementary Methods (Additional file 1).

### Whole-genome sequencing bioinformatics analysis

Following paired-end sequencing with 2 × 101 bp reads on the NovaSeq 6000 system (Illumina, San Diego, California, USA), demultiplexing of samples was performed using BCL Convert (Illumina), and raw sequencing data were processed using the DRAGEN DNA Pipeline on the Illumina DRAGEN Bio-IT Platform (Illumina) v3.9. After adapter trimming, sequencing reads were aligned to GRCh38/hg38. Duplicates and reads with a mapping quality < 30 were removed from analysis. A second bam file with 90–150 bp fragments only was generated for SCNA analysis. In all of the following analysis regions overlapping with ENCODE blacklist [24] and the UCSC gap track [25] were excluded.

### Global and regional fragmentation analysis

Global and regional fragmentation of cfDNA was analyzed as described by Peneder et al. in 2021 [20]. Briefly, fragment length was determined using Picard CollectInsertSizeMetrics (version 2.26.6) and global fragmentation was derived as the fraction of fragments with distinct lengths. Regional fragmentation was established as the z-scored difference in the ratio of short (90–150 bp) to long (151–220 bp) fragments (S/L ratio) in 100 kb bins compared to the 55 healthy controls. Z-scores of the fragmentation of healthy controls were calculated by comparison to the other 54 healthy control samples. Data of genomic regions harboring SCNAs were excluded to avoid bias due to regionally enriched ctDNA. The computational analysis described by Peneder et al. in 2021 [20] was adapted that regions harboring SCNAs were identified based on the SCNA workflow, described below rather than ichorCNA. Furthermore, we used a significant enrichment of short fragments (90–150 bp) as indicator for ctDNA based on global, and significantly different z-scored S/L-ratios on at least one chromosome arm as indictor for ctDNA based on regional fragmentation.

### Coverage in CRC-specific regions of interest

The LIQUORICE tool (v.0.5), developed by Peneder et al. in 2021 [20], was used to identify ctDNA based on significant coverage drops in CRC-specific transcriptionally active chromatin regions (epigenetic signatures). We analyzed the coverage in CRC-specific active chromatin regions, published by Chiara et al. in 2021 [26] and in universal DNase I hypersensitivity sites (DHS). The neighboring 20 kb of each region set were split into 500 bp bins to identify the mean coverage around the regions of interest. To correct for bias due to regionally enriched ctDNA, SCNAs, identified with the SCNA workflow, described below, were provided to LIQUORICE. Significant coverage drops compared to healthy controls in at least two of the analyzed region sets were considered as indicator for ctDNA.

### Somatic copy number alterations

The CNV workflow provided with the Illumina DRAGEN Bio-IT Platform (Illumina) was performed based on 90–150 bp fragments, since higher sensitivity for SCNA calling was previously described for these short fragments [18, 20]. In detail, reads were counted in 50 kb bins, followed by GC bias correction and normalization based on a reference set containing data from 55 healthy control samples. Segmentation was performed by circular binary segmentation with disabled merging of two adjacent segments (merge-threshold = 0). Following the DRAGEN CNV workflow, SCNAs were identified according to the Plasma-Seq pipeline described by Heitzer et al. [15] applying chromosome specific thresholds (Additional file 1: Figure S3, Methods; Additional file 2: Tables S3 and S4).

### Focal somatic copy number alterations

Focal SCNAs, identified within the Plasma-Seq pipeline were defined as described by Ulz and Belic et al. in 2016 [22]. SCNAs of < 20 Mb, overlapping with ≤ 100 genes of the COSMIC cancer gene census [27] with a higher or lower log2 ratio than the chromosome specific LOB compared to the neighboring 20 Mb were identified as focal SCNAs. In addition segments with a higher log2 ratio of 0.58 (~ three copies) compared to the neighboring 20 Mb were identified as focal amplifications, even if no gene of the COSMIC cancer gene census [27] overlapped.

### Machine learning model for tumor detection

For ctDNA detection in samples collected from CRC patients, different machine learning (ML) classifiers were trained as described by Peneder et al. in 2021 [20]. Briefly, support vector machines, feed-forward neural networks, random forests and binomial generalized linear models with elastic-net regularization were trained and evaluated using 100 bootstrapping iterations with fivefold cross-validation in each training set.

We evaluated the performance of ML classifiers on the following feature sets: (i) Global fragmentation, (ii) regional fragmentation, and (iii) a meta-learner (Additional file 1: Methods; Additional file 2: Table S5) [18–20].

For each feature set the support vector machine was selected as best ML classifier to build a final ML model on the complete data.

### Statistical analysis

Differences in global and regional fragmentation of healthy individuals and CRC patients were determined using a Mann–Whitney-U test. Bonferroni correction was used to adjust p-values for multiple testing. All statistical analyses were performed using statistical functions within the Python module SciPy v.1.8 (scipy.stats) with Python version 3.10.

## Results

### Tumor-specific global fragmentation pattern

To establish a comprehensive data set for LB analysis in all stages of CRC, we applied WGS with a median coverage of 6x (SD = 2.37) in 259 plasma samples of CRC patients (*n* = 50) and healthy controls (*n* = 61) (Additional file 2: Table S2). We first evaluated, whether the global fragmentation pattern of cfDNA may be a suitable marker for untargeted ctDNA detection. Fragmentation patterns are a result of various chromatin states that are associated with altered expression of tumor-associated genes [19, 21, 28, 29].

**Fig. 1:**
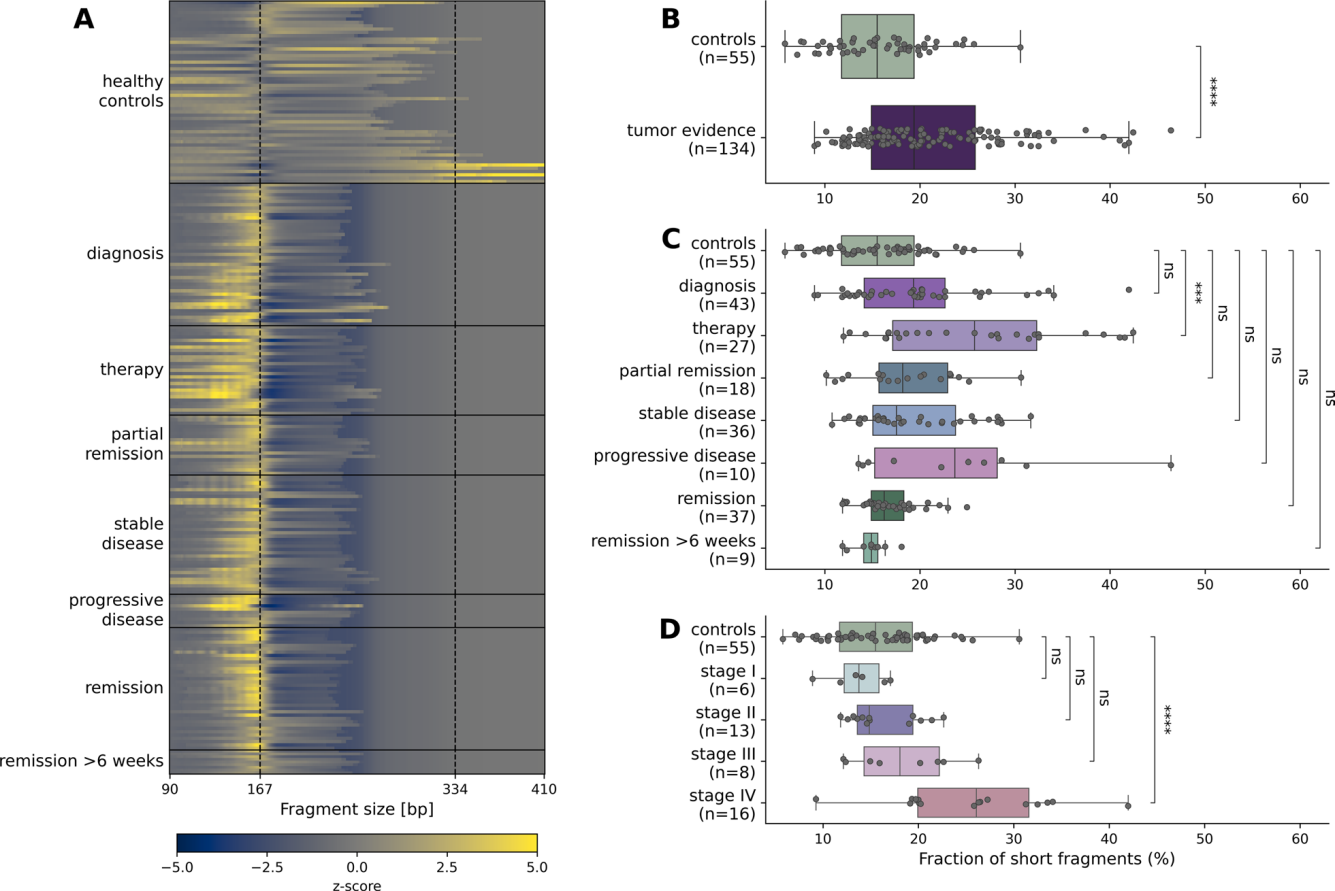
Differences in global fragmentation between cfDNA from CRC patients and healthy controls. **A** Heat map showing enrichment or decrease in cfDNA fragments from 90 to 410 bp according to their length as z-scores of each sample compared to healthy controls. **B** Short cfDNA fragments (90–150 bp) are significantly enriched in samples collected from CRC patients with clinically diagnosed tumor burden. **C** Only for samples collected in the beginning of therapy a significantly enriched fraction of short fragments can be observed. **D** At diagnosis a significant enrichment in short fragments was only observed in patients with stage IV CRC. (ns: *p*-value ≤ 1; *: *p*-value ≤ 5*10^–2^, **: *p*-value ≤ 1*10^–2^, ***: *p*-value ≤ 1*10^–3^, ****: *p*-value ≤ 1*10^–4^)

We compared the global fragmentation of cfDNA from CRC patients to cfDNA from healthy controls which typically present with a peak of ~ 167 bp corresponding to DNA bound by one nucleosome plus linker DNA [20] (Fig. 1A). We observed a significant enrichment of short fragments (90–150 bp) in CRC patient samples with clinically diagnosed tumor burden (*n* = 134) compared to healthy controls (*n* = 55) (Mann–Whitney-U test, *p*-value = 4.75*10^–5^) (Fig. 1B). When allocating CRC patient samples according to the course of disease, we observed a significant enrichment of short fragments during therapy (between surgery and adjuvant chemotherapy, or during chemotherapy before staging) (*n* = 27) (*p*-value = 1.48*10^–4^). A tendency (albeit not statistically significant) toward a higher proportion of short fragments could be identified in all other progression sample groups with clinically diagnosed tumor burden (Fig. 1C). When stratifying CRC patient samples collected at diagnosis according to their disease stage, we further observed a significant enrichment of short fragments in patients with stage IV CRC (*n* = 16) (*p*-value = 7.25*10^–5^) (Fig. 1D).

Interestingly albeit not statistically significant, we detected different fragmentation profiles due to enrichment of short fragments < 167 bp when analyzing samples from CRC patients in remission with no evidence of disease compared to healthy controls. When focusing on samples from CRC patients in remission more than six weeks post-treatment, we did no longer observe an enrichment of short fragments < 167 bp (Fig. 1A). The observed enrichment within the first weeks post-surgery is likely associated with the intake of low-molecular weight heparin, in accordance with previous findings [30, 31]. Taken together, our results indicate that cfDNA is more fragmented in CRC patients compared to healthy controls and can therefore support untargeted detection of ctDNA.

### Tumor-specific regional fragmentation profiles

**Fig. 2:**
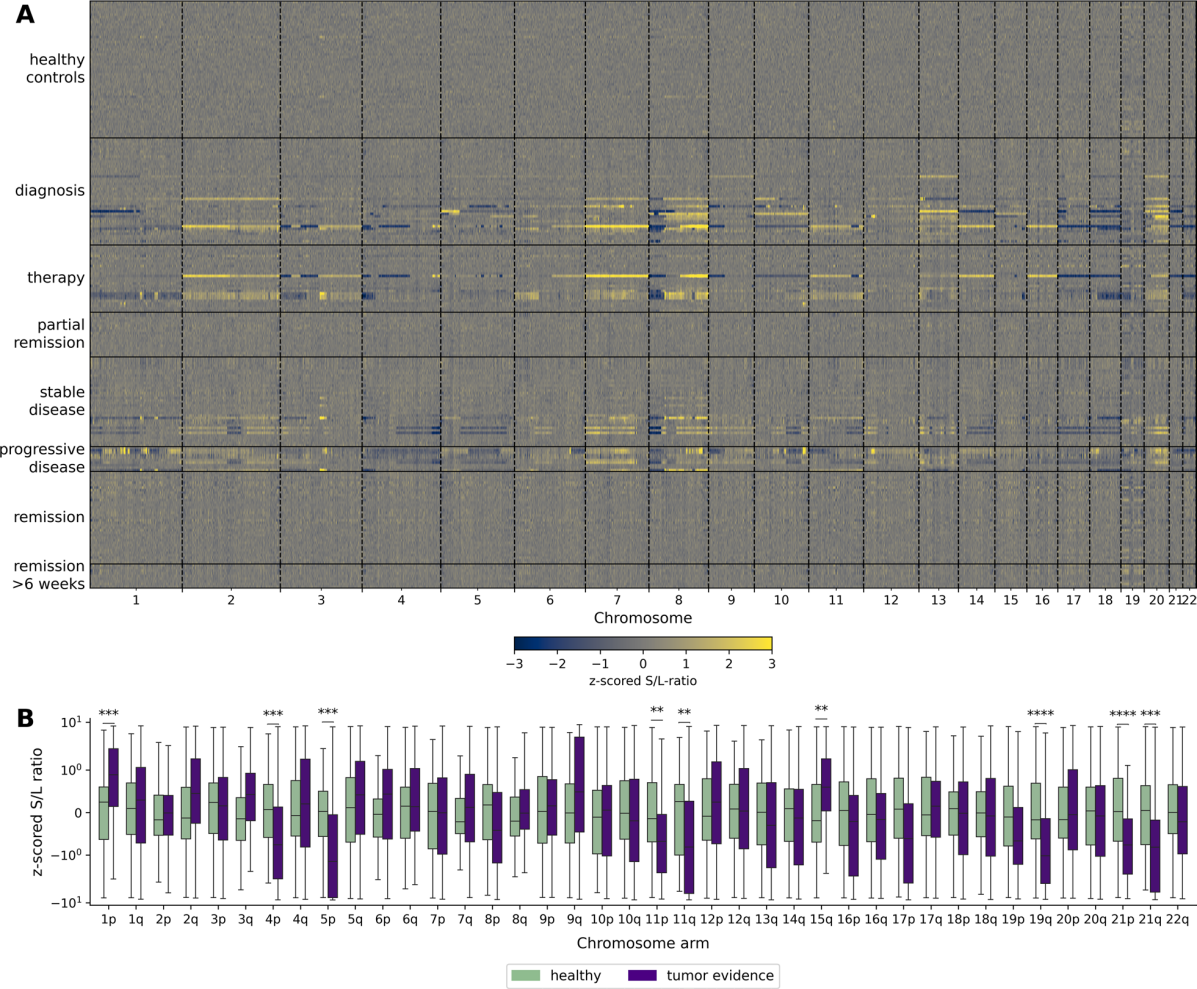
Differences in regional fragmentation between cfDNA from CRC patients and healthy controls. **A** Heat map showing the z -scored of S/L-ratios in 100 kb bins of each sample compared to healthy controls. **B** Significant differences in z-scored S/L-ratios between samples collected from CRC patients with clinically diagnosed tumor burden and healthy controls were observed on multiple chromosome arms. (*: *p*-value ≤ 5*10^–2^, **: *p*-value ≤ 1*10^–2^, ***: *p*-value ≤ 1*10^–3^, ****: *p*-value ≤ 1*10^–4^)

To assess whether regional fragmentation across the genome could serve as another non-genetic marker for ctDNA detection in CRC patients, we calculated the ratio of short (100–150 bp) to long (151–220 bp) fragments (S/L ratio) in 100 kb bins for each chromosome in CRC patients and healthy controls, as recently described [19, 20]. Notably, data of chromosome arms harboring SCNAs were excluded to avoid bias due to regionally enriched ctDNA. Compared to healthy controls, we observed distinct differences in the S/L ratio of CRC patients at diagnosis, during therapy, and with stable or progressive disease. In contrast, in CRC patients with partial remission or in remission, we did not observe such differences (Fig. 2A). Focusing on CRC patient samples with clinically diagnosed tumor burden, we observed a significant enrichment in short fragments on chromosome arms 1p and 15q, and significant enrichment of long fragments on chromosome arms 4p, 5p, 11p, 11q, 19q, 21p and 21q (Fig. 2B). Overall, we were able to detect ctDNA in 75% (100/134) of samples collected from CRC patients with clinically diagnosed tumor burden based on significantly different regional fragmentation on at least one chromosome arm.

The differences in regional fragmentation between CRC patients and healthy controls support recent findings identifying cfDNA fragmentation as independent biological feature representing chromatin profiles of the cells of origin [19, 20].

### Combination of global and regional fragmentation analysis using machine learning

**Fig. 3:**
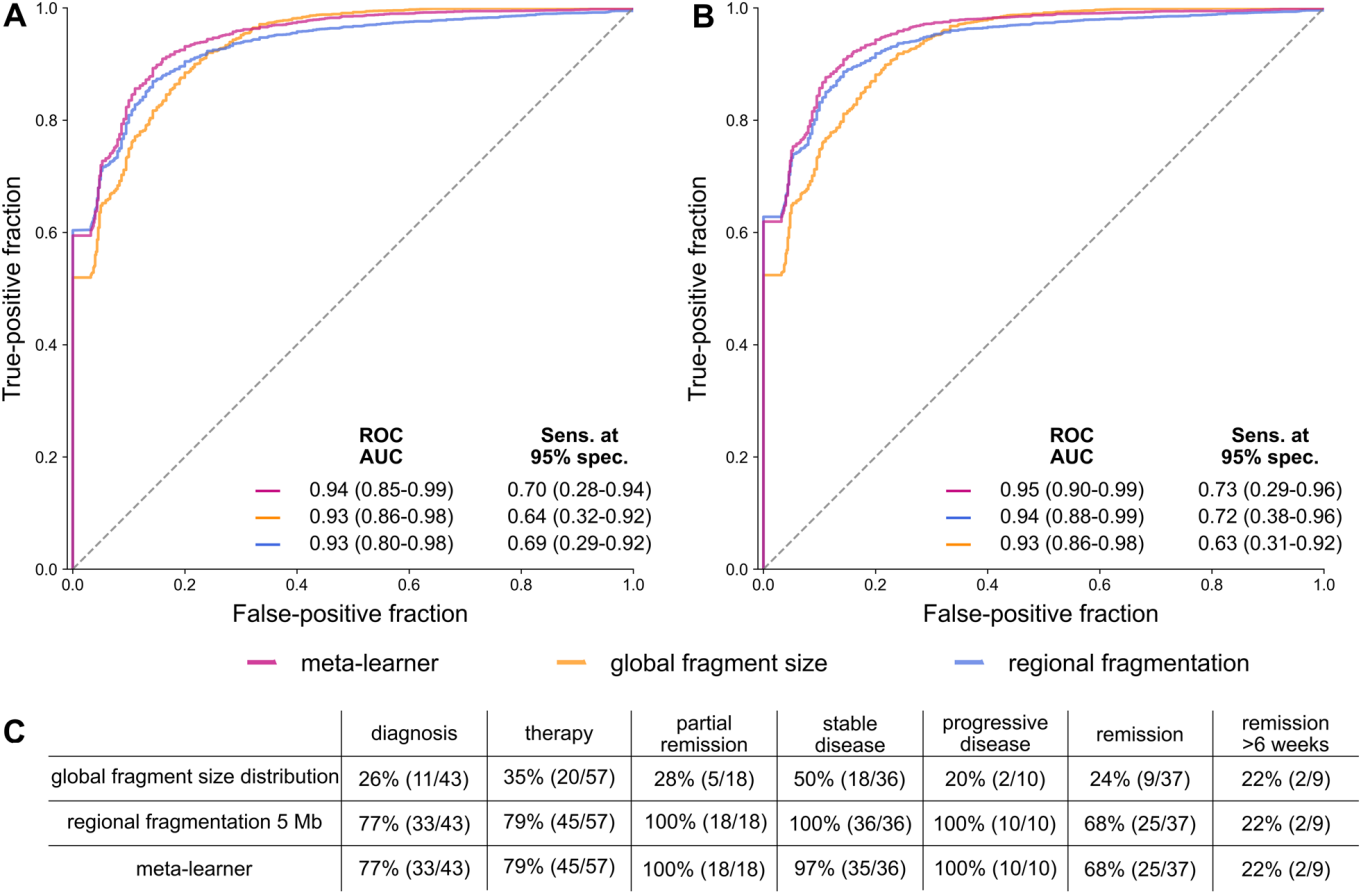
Performance of ML classifiers based on global and regional fragmentation as well as a meta-learner. Performance was assessed over 100 bootstrapping iterations with fivefold cross validation **A** using the best performing model out of four classifiers for each iteration and **B** only a support vector machine over all iterations. **C** The three final classifiers detect ctDNA in CRC patients with high sensitivity

To test whether machine learning (ML) classifier based on global fragmentation and regional fragmentation in 5 Mb bins increase accurate detection of ctDNA, we trained four ML algorithms using 100 bootstrapping iterations with fivefold cross-validation (see Materials and Methods). For each iteration the prediction of the best model was stored and predictions for the two classifiers based on global and regional fragmentation were combined within a supervised meta-learner [20]. Samples collected from CRC patients with clinically diagnosed tumor burden (*n* = 134) served as positive cohort, and healthy individuals, including samples collected from patients in remission more than six weeks post-treatment without any known recurrence at a later time point (*n* = 63) served as control cohort for a better representation of biological variability (Additional file 1: Figure S1). All classifiers showed high prediction performance to distinguish cfDNA from CRC patients and healthy controls, with receiver operating characteristic (ROC) area under the curve (AUC) values of up to 94% and sensitivity at 95% specificity of up to 70% (Fig. 3A). Since our ultimate goal was to develop a workflow applicable in clinical practice, we trained a final model based on the best performing ML algorithm for each feature set. Evaluating the performance of ML classifiers using only the support vector machine, we observed ROC AUC values and sensitivity at 95% specificity of up to 95% and 75%, respectively (Fig. 3B). Eventually, we trained final ML models for both feature sets as well as the meta-learner including all data of CRC patients (*n* = 134) and controls (*n* = 63) without further subsetting. Applying these models with 95% specificity, ctDNA presence was correctly predicted in 36% (48/134) of samples based on global fragmentation (34/91 metastatic, 14/43 localized), and in 90% of samples based on regional fragmentation (121/134: 85/91 metastatic, 36/43 localized) and based on the meta-learner (120/134: 84/91 metastatic, 36/43 localized). However, also samples collected from patients in remission, especially within the first six weeks post-surgery were classified as ctDNA positive (Fig. 3C). These results in combination with the findings above indicate that the non-genetic cfDNA features analyzed within LIFE-CNA are not informative for the correct identification of ctDNA within the first six weeks post-surgery. However, the effects of surgery on cfDNA fragmentation seem to normalize after six weeks, indicating a potential use for recurrence monitoring starting at this time point.

### CRC-specific active chromatin for ctDNA detection

We evaluated whether CRC specific chromatin signatures can be detected based on coverage changes using the LIQUORICE tool [20] and whether these chromatin signatures represent an independent marker for ctDNA detection. Specifically, we analyzed five sets of enhancer regions identified to be active in CRC including (i) active distal ChromHMM-defined [32] enhancer regions, (ii) CRC-specific gained enhancers identified by Hi-C [33], (iii) gained enhancers occupied by the transcriptional coactivators YAP/TAZ, (iv) highly conserved enhancers occupied by YAP/TAZ, and (v) active transcriptional start sites (TSS) in CRC [26]. In addition, we analyzed the coverage in universal DHS. In total, we observed significantly stronger coverage drops in all region sets in samples collected from CRC patients compared to healthy controls. In 5% (3/55) of healthy controls significantly stronger coverage drops in one of the analyzed region sets were detected when comparing the coverage to all 54 other healthy control samples. Therefore, to ensure a specificity of ≥ 95% for ctDNA detection based on the coverage in CRC-specific active chromatin regions, significantly stronger coverage drops need to be identified in at least two of the analyzed region sets rather than one. Overall, we detected ctDNA based on differential coverage in 33% (44/134) of samples collected from CRC patients with clinically diagnosed tumor burden (Additional file 2: Table S6). However, we obtained similar values [32% (12/37)] for remission patients and [33% (3/9)] for remission patients more than six weeks post-treatment. Taken together, coverage-based chromatin site analysis for ctDNA detection is suitable at diagnosis, but not for recurrence (also not > 6 weeks).

### Quantification of the ctDNA fraction in CRC patients

To quantify the ctDNA fraction as a complement to fragmentation and coverage-based chromatin site analysis, we used the ichorCNA tool [17], which led to correct prediction of ctDNA in only 35% (47/134) of samples with clinically diagnosed tumor burden, even when selectively enriching for ctDNA-associated 90–150 bp fragments (Additional file 1: Figure S3) [18, 20].

### Detection of genome-wide and focal SCNAs in CRC patients

**Fig. 4:**
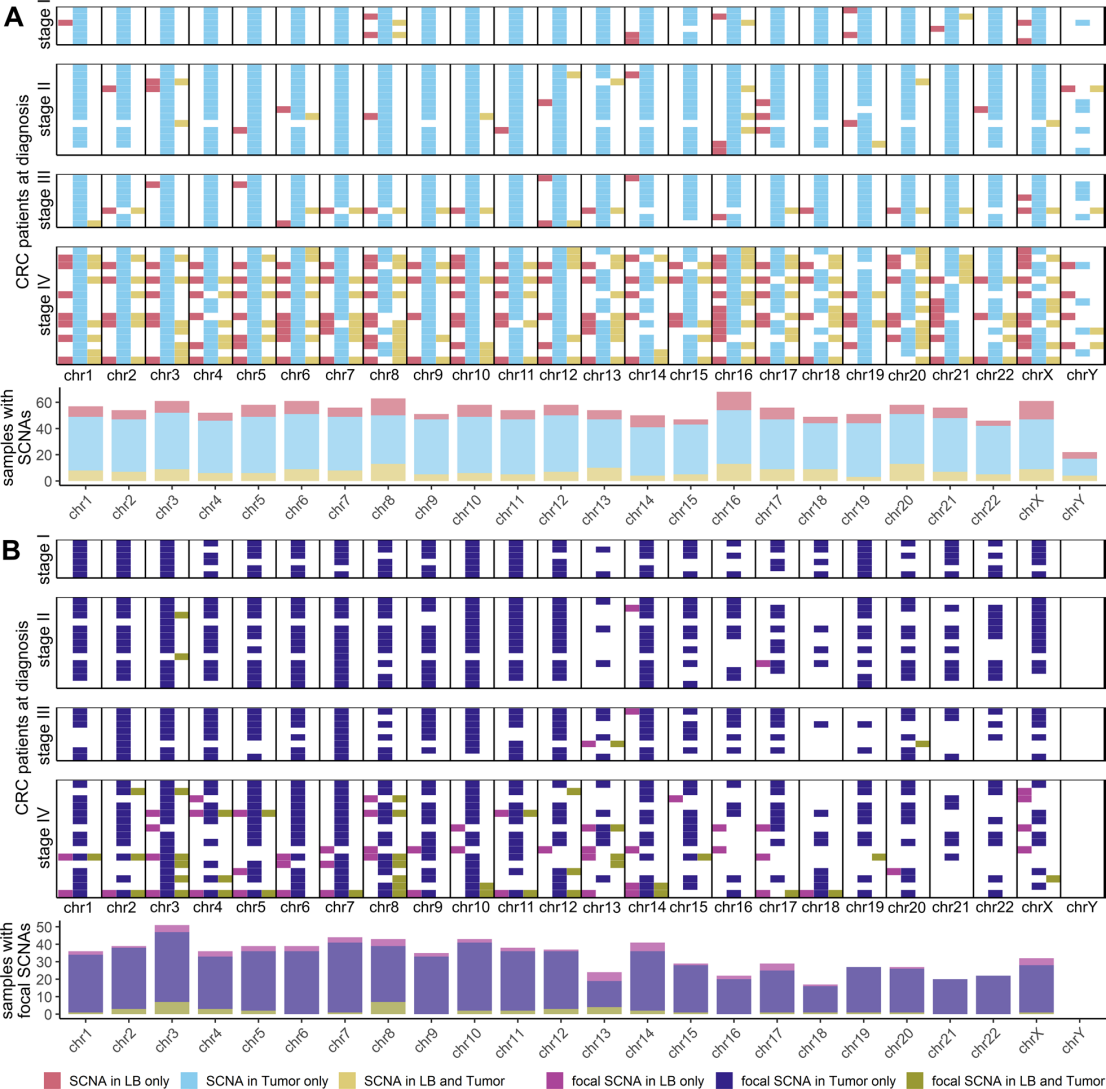
Matched plasma and tumor analysis. To validate the SCNA analysis integrated in LIFE-CNA we performed a matched analysis of plasma samples collected at diagnosis with tumor tissue. **A** Total SCNAs present in plasma (red) or tumor (blue) only or in both plasma and tumor (yellow) and **B** focal SCNAs present in plasma (pink) or tumor (violet) only or in both plasma and tumor (green) present on each chromosome for individual patients and summarized over all patients below. Since more than one SCNA can be present per chromosome, it is possible that on the same chromosome different SCNAs are detected in plasma only, tissue only or in both plasma and tumor tissue

To identify genome-wide and focal SCNAs we applied a combination of the Illumina DRAGEN CNV workflow and Plasma-Seq [15, 22], considering ctDNA-associated 90–150 bp fragments (Additional file 1: Methods, Figures S4 and S5) [18, 20]. We analyzed paired tumor tissue and plasma samples collected at diagnosis to validate the SCNA pipeline. To correct for germline CNVs, constitutional DNA from saliva was additionally analyzed. In 44% (12/27) of patients with localized- and in 94% (15/16) of patients with metastatic CRC genome-wide SCNA profiles were highly concordant to the corresponding tissue. SCNAs unique to plasma were identified in 78% (21/27) of patients with localized- and 82% (13/16) of patients with metastatic CRC (Fig. 4A). In addition, we identified focal SCNAs in plasma matching tumor tissue in 4% (1/27) of patients with localized-, and in 63% (10/16) of patients with metastatic CRC, and focal SCNAs only in plasma in 15% (4/27) of patients with localized-, and in 63% (10/16) of patients with metastatic CRC (Fig. 4B). Certain genetic events found in plasma may not be present in tumor tissue because of the representation of only one site of the entire tumor mass rather than the complete tumor heterogeneity including metastatic sites. It is likely that low amplitude SCNAs may not be detected in plasma since ctDNA represents only a fraction of total cfDNA. Overall, although some SCNAs might be missed in plasma, with our approach we are able to detect genome-wide SCNAs in plasma from CRC patients over all stages, including subclonal events not identified in tumor tissue.

### Complementary ctDNA detection by combining cfDNA features

**Fig. 5:**
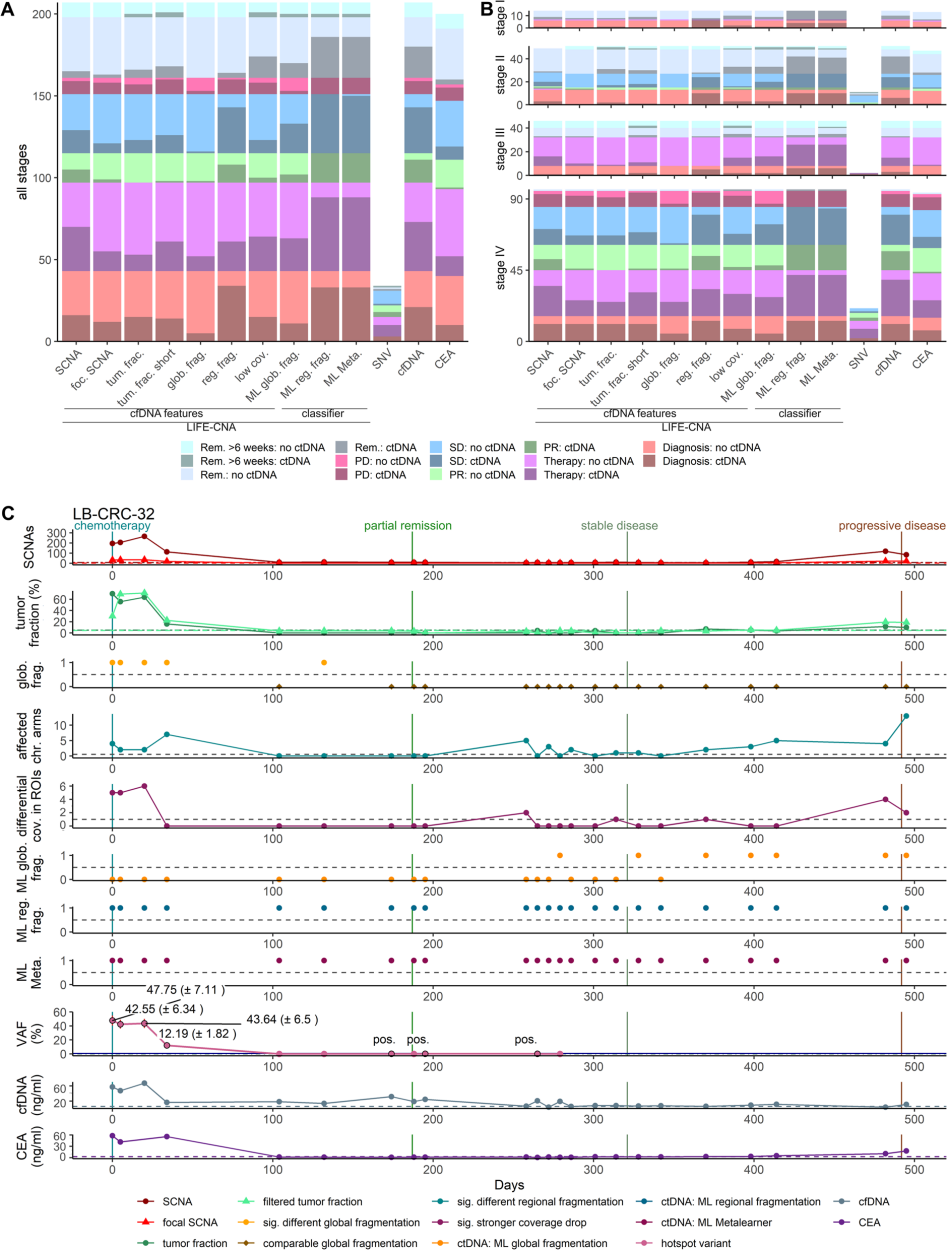
LIFE-CNA enables accurate disease monitoring in CRC patients. SCNAs, focal SCNAs (foc. SCNA), tumor fraction in all (tum. frac.) and filtered fragments (tum. frac. short), enrichment in fragments from 90 to 150 bp (glob. frag.), regional fragmentation (reg. frag.), and significantly stronger coverage drops (low cov.) were analyzed with LIFE-CNA. In addition ctDNA was predicted with machine learning classifiers based on global (ML glob. frag.) and regional fragmentation (ML reg. frag.), and a meta-learner (ML Meta.) integrated into LIFE-CNA. To assess performance of LIFE-CNA, hotspot variants (SNVs) cfDNA concentration (cfDNA) and CEA were analyzed **A** in samples from CRC patients collected at different time points during disease summarized over all samples and **B** stratified by disease stage. **C** LB-CRC-32 was used as one example to show response and resistance to treatment throughout the course of disease

Based on our results showing that global and regional fragmentation as well as chromatin signatures, and SCNAs are capable to independently detect ctDNA, we compared the sensitivity of all features in CRC patients in general and across stages considering the time point of sample collection in the course of disease (Fig. 5A, B).

Regional fragmentation and coverage in active chromatin enabled ctDNA detection in 77% (33/43) and 23% (10/43) of patients with localized- and in 74% (67/91) and 37% (34/91) of patients with metastatic CRC with clinically diagnosed tumor burden, respectively. As expected, increased numbers of called SCNAs as well as elevated tumor fractions (Additional file 1: Data) were mainly observed in patients with metastatic CRC (57%, 52/91 vs. 26%, 11/43 and 45%, 41/91 vs. 14%, 6/43, respectively). Enriched short cfDNA fragments enabled ctDNA detection only in a small number of patients with metastatic CRC (19%, 17/91). Considering the three ML classifiers integrated in our LIFE-CNA workflow, we observed that the classifiers based on regional fragmentation and the meta-learner have a higher sensitivity for ctDNA detection (90%, 121/134 and 120/134, respectively), compared to the classifier based on global fragmentation (36%, 48/134). However, when focusing on samples collected within the first six weeks post-surgery, we observed ctDNA predictions with all non-genetic cfDNA features besides the global fragmentation, with the highest numbers of 68% (25/37) being with the ML classifiers based on regional fragmentation and the meta-learner. When focusing on only those samples collected from patients in remission more than six weeks post-treatment ctDNA detection rates decreased.

### LIFE-CNA for accurate treatment monitoring in CRC patients

The analysis of multiple ctDNA features improves the sensitivity of untargeted ctDNA detection. To assess the clinical validity of LIFE-CNA for disease monitoring, we assessed changes of our measures over a median follow-up time of 7.5 months (range 1–35.5 months) in 15 patients and correlated these changes with treatment outcome as a proof-of-concept (Additional file 2: Table S6). In addition to LIFE-CNA, we analyzed the commonly used serum protein marker CEA, plasma cfDNA concentration, and SNVs for patients with available hotspot variant data (*n* = 5). We were able to predict response to treatment in 77% (10/13) of patients (7/7 metastatic, 3/5 localized) by decreasing numbers of SCNAs, normalizing regional or global fragmentation, and/or normalizing coverage in regions of interest. CEA was informative in only 25% (3/12) of patients in two of those patients ~ 2 months later than LIFE-CNA, and decreasing plasma cfDNA concentrations could be correlated to treatment response in only 46% (6/13) of patients in one of those patients ~ 1 month later than LIFE-CNA. Further, LIFE-CNA correctly predicted progressive disease in 100% (5/5) of patients up to four months before clinical evidence with increasing differences to healthy controls of all analyzed cfDNA features. CEA was informative in only 80% (4/5) of patients in one of those patients ~ 3.5 months later than LIFE-CNA and cfDNA concentration was informative in only 20% (1/5) of patients ~ 9 months later than LIFE-CNA, respectively (Additional file 1: Figures S6–S20). For example, response and resistance to treatment could be detected with LIFE-CNA in patient LB-CRC-32 up to five and three months before clinical evidence, respectively (Fig. 5C). CEA identified response to treatment > 2 months later and resistance to treatment in parallel to LIFE-CNA. Although, decreasing cfDNA concentration was associated with response to treatment, at the time of progression no increase could be observed which is in line with previous reports showing low sensitivity and specificity of cfDNA concentration for treatment monitoring [34]. For SNVs, response to treatment could be identified in 3/4 samples, whereas no data were available to evaluate changing SNV levels for progression detection.

### LIFE-CNA for cancer screening but not for MRD

To analyze whether LIFE-CNA could be applied for the detection of MRD post-surgery, plasma samples of 33 CRC patients collected up to 8 days pre-surgery and follow-up samples collected between 1 and 42 days post-surgery were analyzed (Additional file 1: Figure S21). Pre-surgery, we detected ctDNA in 92% (22/24) of patients with localized- and in 89% (8/9) of patients with metastatic CRC. Post-surgery, ctDNA was identified in 96% (23/24) of patients with localized- and in 100% (9/9) of patients with metastatic CRC, in particular due to the classifiers based on regional fragmentation and the meta-learner. Further, significant differences in coverage were observed in a large number of post-surgery samples (Additional file 1: Figures S21–S54). Decreasing ctDNA predictions more than six weeks post-treatment might enable the application of LIFE-CNA for recurrence monitoring (Fig. 5A&B, turquoise: remission more than six weeks post-treatment). In addition, the high sensitivity of ctDNA detection at diagnosis of patients with localized CRC (92%) suggests the great potential of LIFE-CNA for cancer screening.

### Proof-of-principle of LIFE-CNA using six healthy controls and in silico dilutions

**Fig. 6:**
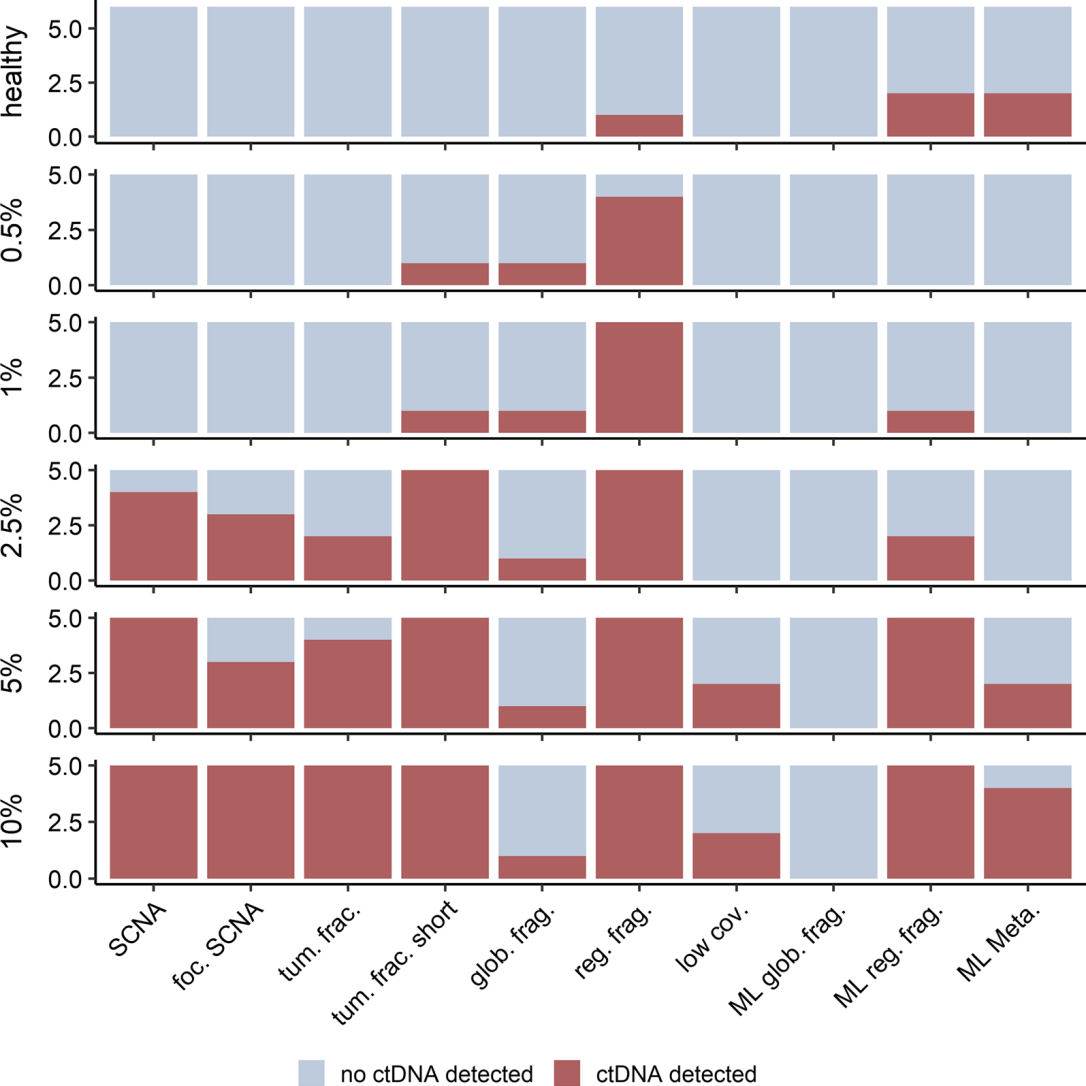
Proof-of principle showing the high sensitivity of LIFE-CNA. Focal SCNAs (foc. SCNA), tumor fraction (tum. frac.), tumor fraction in 90 to 150 bp fragments(tum. frac. short), enrichment in fragments from 90 to 150 bp (glob. frag.), differential regional fragmentation (reg. frag.), significantly stronger coverage drop in at least to region sets (low cov.), classifier based on global fragmentation (ML glob. frag.), classifier based on regional fragmentation (ML reg. frag.), and classifier based on meta-learner (ML Meta.) were analyzed in six additional healthy controls not included in the panel of normals and in in silico dilutions with 0.5%, 1%, 2.5%, 5% and 10% tumor fraction as a proof-of-principle for ctDNA detection using LIFE-CNA

We evaluated the specificity of all cfDNA features by analyzing six additional healthy controls not included in the reference set. Of all analyzed cfDNA features only differential regional fragmentation was detected in 1/6 healthy controls while the remaining cfDNA features did not indicate ctDNA (Fig. 6). The ML classifiers based on regional fragmentation and the meta-learner, predicted ctDNA in 2/6 healthy controls. These results indicate low specificity of the regional-fragmentation and meta-learner based classifiers for ctDNA detection.

In addition to specificity, we also assessed the sensitivity of LIFE-CNA for the detection of low ctDNA levels using in silico dilutions with tumor fractions of 0.5%, 1%, 2.5%, 5% and 10% (Additional file 2: Table S7). Analogous to disease monitoring, also for the in silico dilutions we observed the highest sensitivity for ctDNA detection based on regional fragmentation that correctly identified ctDNA in 4/5 samples with 0.5% tumor fraction and in all samples with 1% tumor fraction. At 0.5% tumor fraction, elevated tumor fractions based on ichorCNA and significant enrichment of short fragments could be predicted in one sample. Further, SCNAs could be detected in 4/5 samples with 2.5% tumor fraction. These results indicate that the sensitivity of our SCNA analysis could be increased compared to the previously described required tumor fractions above 5% to 10%. Focusing on the ML classifiers for ctDNA prediction, it was not possible to detect ctDNA based on global fragmentation in any of the in vitro dilutions. Using the classifier based on regional fragmentation, we detected ctDNA in 1/5 samples with 1% tumor fraction.

## Discussion

Non-invasive and highly-sensitive ctDNA analyses allow real-time monitoring of patients throughout disease. The untargeted detection of ctDNA has the potential to extend the advantages of LB analysis to patients with cancer across all stages, and independently from knowledge about the presence of somatic hotspot variants. However, clinical validity of untargeted ctDNA analysis could so far mainly be shown for patients with metastatic cancer due to their high tumor fractions. Here, we developed LIFE-CNA for genome-wide ctDNA detection and disease monitoring based on multiple tumor-specific alterations across genetics, epigenetics and fragmentomics in patients with localized and metastasic CRC. We further provide analytical validation as well as a clinical proof-of-concept using a total of 259 plasma samples from 50 CRC patients and 61 healthy individuals. In contrast, a similar study conducted by Cristiano et al. [19] focused on one cfDNA feature (regional fragmentation analysis) for ctDNA detection only. Another study by Peneder et al. [20] also analyzed multiple cfDNA features in Ewing-sarcoma patients.

To facilitate clinical implementation of genome-wide ctDNA analysis suitable for all CRC patients, we defined distinct cutoffs or significance tests for each analyzed cfDNA feature. Establishing and validating definite criteria to report true ctDNA signals further are an important step towards the development of generic guidelines for the analytical validation of untargeted LB analyses, complementing the existing guidelines for targeted hotspot analyses [9, 35].

We evaluated performance of the various cfDNA features and of ML classifiers. CfDNA features achieved a higher sensitivity than ML classifiers for ctDNA detection at diagnosis of patients with localized and metastatic CRC, while false-positive predictions in external healthy controls were higher with the ML classifiers. Other applied ML classifiers reported in the literature achieved slightly better performance characteristics from training and testing procedures for early detection of ctDNA [20, 36]. One previous study performed external validation of a final ML classifier on a cohort of lung cancer patients and thereby achieved comparable sensitivity with slightly higher specificity compared to our ML classifier [37]. Although thorough external validation is required, considering an (albeit small) set of external samples indicates that our ML classifier might achieve a similar performance for CRC patients. Besides focusing solely on ML classifiers or the analysis of multiple cfDNA features, we also investigated whether a combination of ML classifiers with the analysis of multiple cfDNA features can improve the sensitivity of untargeted ctDNA detection. Concretely, combining the analysis of global and regional fragmentation, SCNAs and active chromatin coverage with the ML classifiers resulted in a slightly improved sensitivity for ctDNA detection at diagnosis of patients with localized and metastatic CRC and increased false-positive predictions in external healthy controls. We conclude that considering cfDNA features without ML classifiers may be favorable in cancer screening, as the number of false-positives is markedly reduced, with only a limited reduction in sensitivity, providing comparable performance to colonoscopies, the current gold standard in CRC screening [38]. However, before clinical implementation of LIFE-CNA, sensitivity and specificity needs to be externally validated in a larger cohort.

When evaluating the clinical sensitivity and specificity of LIFE-CNA for residual disease detection and treatment monitoring in a proof-of-concept study, we find a (too) high number of ctDNA positive predictions in R0-resected patients within the first six weeks post-treatment, showing that LIFE-CNA is probably not suited for residual disease detection. This may be explained by the fact that gene regulation and cfDNA fragment length, both factors being considered in the cfDNA features of LIFE-CNA, are perturbed after surgery. Multiple studies described altered gene regulation following surgery in response to cellular trauma [39–41] and the association of low-molecular weight heparin with increased levels of short cfDNA fragments [30, 31], which is given to patients directly after treatment.

There are some limitations that should be considered. Training the ML classifiers on a small a cohort of 134 CRC patient samples and 63 controls might cause overfitting. To overcome false-positive predictions caused by biological variability, larger control and positive cohorts to improve training and external validation for testing would be required before implementation of ML classifiers into clinical practice becomes feasible. Further, the median age of CRC patients (73) is much higher than the median age of healthy controls (32). With regard to the association between cfDNA fragmentation and nucleosome occupancy, which may change during life, future studies with age-matched healthy controls are highly important for validation of LIFE-CNA. Another limitation of this study is that a retrospective analysis of our rather small cohort enabled only the evaluation of a clinical proof-of-concept of LIFE-CNA but not the clinical validity. To establish the clinical utility a large prospective study would be required. If the clinical validity and utility of LIFE-CNA are demonstrated, simple blood sampling may allow rapid and non-invasive treatment monitoring, avoiding unnecessary colonoscopies and radiation introduced by imaging.

## Conclusions

Taken together, we assume that considering multiple cfDNA features across different types of tumor-specific alterations in an untargeted genome-wide approach and evaluating them for various applications including screening and treatment monitoring, is an important step toward translating the high potential of liquid biopsy for future personalized medicine applications. Further, when analyzing active chromatin regions specific to other tumor entities we believe that LIFE-CNA can be easily transferred to all solid tumors.

## Supplementary Information

**Additional file 1**: Methods and Data including Table S1 and Figures S1–S54. **Methods** contain more detailed information about the study cohort and methods required to reproduce the experiments. **Data** contain detailed information on results not shown in the main document and results obtained for each individual patient analyzed for residual disease detection or treatment monitoring.

**Additional file 2:** Data required to reproduce the results shown in Tables S2–S8.

## Data Availability

The sequence data have been deposited at the European Genome-phenome Archive (EGA) under accession number EGAS00001006490. These data are available under a controlled access regimen to ensure the protection of personally identifiable data; access can be obtained by contacting A.H. and J.P. Remaining data generated or analyzed during this study are included in this published article, and its supplementary information files or from the corresponding author on reasonable request. Code for sample analysis is modified from the publication by Heitzer et al. [15], Ulz et al. [22] and Peneder et al. [20] and is available from the corresponding author on reasonable request.

## Abbreviations

CEA: Carcinoembryonic antigen
cfDNA: Circulating free DNA
CNV: Copy number variation
CRC: Colorectal cancer
ctDNA: Circulating tumor DNA
ddPCR: Droplet digital PCR
DHS: DNase I hypersensitivity sites
LB: Liquid biopsy
LIFE-CNA: LIquid biopsy Fragmentation, Epigenetic signature and Copy Number Alteration analysis
ML: Machine learning
MRD: Molecular residual disease
S/L ratio: Short-to-long ratio
SCNA: Somatic copy number alteration
SNV: Single nucleotide variant
TSS: Transcriptional start site
WGS: Whole-genome sequencing
