# Supplementary material for: Somatic copy number alteration and fragmentation analysis in circulating tumor DNA for cancer screening and treatment monitoring in colorectal cancer patients
Source: J Hematol Oncol. 2022 Sep 2;15:125. doi: 10.1186/s13045-022-01342-z (PMC9438339; doi:10.1186/s13045-022-01342-z)
Supplement: Supplementary file 1 — Additional file 1: Methods and Data including Table S1 and Figures S1–S54. Methods contain more detailed information about the study cohort and methods required to reproduce the experiments. Data contain detailed information on results not shown in the main document and results obtained for each individual patient analyzed for residual disease detection or treatment monitoring. [file 13045_2022_1342_MOESM1_ESM.pdf]

Supplementary Methods

Clinical sample collection and categorization

198 plasma samples from 50 CRC patients, including 21 females and 29 males with a median age of 73 (39 to 88) were collected at diagnosis and at routinely scheduled follow-up examinations of CRC patients until death or patient withdrawal from the study. Follow-up examinations were conducted in accordance to the German S3-guidelines every six months in the first two years after surgery and once a year in the third to fifth year post-surgery. Throughout treatment patient plasma samples were collected every two to four weeks before administration of treatment, if possible. Saliva was collected as germline reference in parallel to first plasma sample collection for 45 of the 50 CRC patients. Tumor tissue was retrieved during diagnostic colonoscopy or primary surgery for all 50 CRC patients. Clinical data were collected for all patients throughout the course of disease and categorized according to the time of sample collection (Supplementary Table 1). Samples collected at baseline before treatment were categorized as 'diagnosis', samples collected between surgery and adjuvant chemotherapy or during chemotherapy before staging were categorized to 'therapy', and samples collected following the clinical diagnosis of 'partial remission', 'stable disease', 'progressive disease', or 'remission' were added to the appropriate categories. Diagnosis and staging of patients were performed during routinely scheduled follow-up examinations by colonoscopy and/or imaging. Plasma samples were retrospectively analyzed without prior knowledge of patients´ outcome.

Plasma samples from 61 healthy individuals, including 28 females and 33 males with a median age of 32 (20 to 73) were collected at a singular time-point.

Control samples

For positive controls used for Droplet Digital PCR (ddPCR), *in vitro* dilutions containing mutant variant allele frequencies (VAFs) ranging from 1% to 5% were generated by spiking genomic DNA (gDNA) from cell lines into gDNA from whole blood of healthy controls. For *BRAF* p.V600E variant, gDNA from SK-MEL-28 cell line (#Cat 300337GD1) and for *KRAS* p.G12S variant, gDNA from A-549 cell line (#Cat300114GD1) were purchased from CLS (Eppelheim, Germany). For wild-type (WT) controls, gDNA from whole blood of healthy individuals was used.

Sample preparation

Blood samples were collected in Cell-Free DNA BCT (STRECK, La Vista, Nebraska, USA, #Cat 230244) tubes. Plasma was separated from whole blood by centrifugation at 1,600 x g for 10 min at room-temperature

(RT) and was transferred to a new 15 ml reaction tube, followed by two centrifugations at 16,000 x g for 10 min at 4°C. 500 µl of plasma were transferred to a separate 1.5 µl reaction tube for CEA analysis. Plasma was stored at -80°C until further sample processing.

cfDNA extraction

Cell-free DNA from 2 to 7.5 ml plasma was isolated using the QIAamp circulating nucleic acid kit (Qiagen, Hilden, Germany, #Cat 55114). All buffer volumes were adjusted to the respective plasma volumes. All membrane washing steps were performed twice. cfDNA concentration was quantified using the High Sensitivity NGS Fragment Analysis Kit (Agilent, Santa Clara, California, USA, #DNF-474-0500) on the Fragment Analyzer system (Agilent).

gDNA extraction

Genomic DNA from whole blood was extracted on a Biomek® FX system (Beckman Coulter, Brea, California, USA) using the NucleoMag® Blood 3 ml Kit (Machery-Nagel, Düren, Germany, #REF 744502.1) as by manufacturer's instructions. gDNA from saliva samples was extracted using the prep IT L2P (DNAGenotek, Ottawa, Ontario, Canada, #PT-L2P) kit as by manufacturers' instructions. gDNA from formalin-fixed paraffin embedded (FFPE) tissue was extracted using the QIAamp DNA FFPE tissue kit (Qiagen, #Cat 56404) as by manufacturers' instructions. Concentration of gDNA was determined using a Slide-200 on an Xpose – Benchtop Spectrophotometer (Trinean, Pleasanton, California, USA).

Droplet Digital PCR

Droplet Digital PCR (ddPCR) was performed using single Probe ddPCR *BRAF* p.V600E assay (Bio-Rad, Hercules, California, USA, #dHsaMDV2010027) and *KRAS* p.G12/p.G13 screening kit (Bio-Rad, #1863506) on the QX200 system (Bio-Rad) according to the manufacturer's instructions (see also Supplementary Methods). DNA was mixed with 10 µl of ddPCR™ Supermix for Probes (no dUTPs) (Bio-Rad, #Cat 1863023) and 1 µl of the primer/probe mixture. For gDNA from whole blood, cell lines or tumor specimens, 5 µl of extracted DNA at a concentration of 4-6 ng/µl were used in single reactions. For cfDNA from plasma samples, 1 µl, 5 µl or 8 µl of extracted DNA at concentrations of >15 ng/µl, between 2 ng/µl and 15 ng/µl, and between 0.6 ng/µl and 2 ng/µl respectively, were used in three replicates. 70 µl of Droplet Generation Oil for Probes (Bio-Rad, #Cat 1863005) was added to the reaction mix for droplet generation in a DG8 cartridge. Droplets were transferred into a 96-well plate (Bio-Rad, #Cat 12001925) and thermal cycled (Eppendorf, Hamburg, Germany, Mastercycler pro): 10 minutes at 95°C, 40 cycles of 94°C for 30 s, 55°C for 1 minute followed by 98°C for 10 minutes (Ramp Rate 2°C/sec). Gating was performed based on mutant variant and wild-type (WT) control samples, and variant populations were identified. ctDNA was quantified

in terms of the mutant VAF. In the WT control, background was determined using the QX Manager software (Bio-Rad, v.1.1). The VAF describes the abundance of detected mutant alleles within all detected alleles, including mutant and WT alleles and is calculated as follows (Supplementary Equation 1):

Supplementary Equation 1 Variant allele frequency

VAF: Variant allele frequency; N_{mut}: Number of mutant alleles; N_{WT}: Number of wild type alleles

$$VAF\ (\%) = \frac{N_{mut}}{N_{mut} + N_{WT}} * 100$$

The LOB and LOQ of ddPCR assays were established in accordance to the "Protocols for determination of limits of detection and limits of quantitation" [1] as previously described [2]. For determination of the LOB at least 60 healthy controls were measured to establish the cutoff for ctDNA detection with ≥95% specificity. For determination of the LOQ at least 40 replicates with the targeted variant present in a known VAF were measured to assess the assay specific dispersion. Samples with VAFs > LOB (limit of blank) are defined with ctDNA positive status, and samples with VAFs > LOQ (limit of quantification) harbor quantifiable ctDNA VAFs.

CEA analysis

CEA levels were determined in plasma samples collected for cfDNA isolation using the Human CEA ELISA Kit (Biorbyt, Cambridge, UK, Cat# orb438561) according to manufacturer´s instructions.

Library preparation and sequencing

Whole-genome sequencing of cfDNA and gDNA samples was performed using the NEBNext® Ultra™ DNA Library Prep Kit for Illumina® (New England Biolabs, Ipswich, Massachusetts, USA, #E7370) as by manufacturers' instructions. 5 to 10 ng of cfDNA, and 20 ng of gDNA samples were used as input for material for library prep. Fragmentation of gDNA from saliva and FFPE tissue was performed with a pulsed shearing program on the Covaris E220 system (Covaris, LLC., Woburn, Massachusetts, USA) in 8 microTUBE-50 AFA Fiber Strip V2 (Covaris, #PN 520174) with the following settings: 18 repeats of 10 sec treatment with a peak incident power of 75 W, 15% duty factor, and 500 cycles per burst at 7°C. Libraries from cfDNA and gDNA from saliva were amplified with ten PCR cycles. gDNA from FFPE tissue was treated with the NEBNext® FFPE DNA Repair Mix (New England Biolabs, #M6630) prior to library prep, and libraries were amplified with 12 PCR cycles. Libraries were quantified using a High Sensitivity DNA Kit (Agilent, #5067-4626) on the Bioanalyzer system (Agilent). Paired-end sequencing with 2x101 bp reads was performed on the NovaSeq 6000 system (Illumina, San Diego, California, USA).

Details for whole-genome sequencing bioinformatics analysis

Tumor content quantification based on ichorCNA

Tumor content of cfDNA samples was quantified with ichorCNA, a tool specifically designed to identify genome-wide SCNAs and predict tumor content in cfDNA samples [3]. To estimate tumor fractions in all fragments and only 90-150 bp fragments, ichorCNA analysis was performed with both inputs. Reads were counted in 500 kb bins using the readCounter tool from the HMMcopy R package v1.36.0 [4]. For ichorCNA analysis samples were normalized to a reference set of 55 healthy controls. The LOB for tumor content estimation was established based on a non-parametric approach allowing an α-error of 5% (Supplementary Equation 2).

Supplementary Equation 2 Determination of the LOB using a non-parametric approach

LOB: Limit of blank; N_B: Number of healthy controls; $P_{1-\alpha}$: Percentile at level 1-α

$$LOB = Result\ at\ position\ [0.95 \times N_B + 0.5] = P_{1-\alpha}$$

Therefore, tumor content estimation was only accounted for samples with a predicted tumor content above this cutoff. Tumor fractions ranged from 5.1% to 70.1% and from 4.7% to 75.9% when including all fragments or 90 to 150 bp fragments, respectively.

Somatic copy number alterations

Sex- and chromosome-specific cutoffs for the identification of somatic copy number gains (positive LOB) and losses (negative LOB) were established based on the distribution of log2 ratios in each bin of 55 healthy controls for 80% confidence intervals (CIs)(Supplementary Equation 3). Log2 ratios, deviating more than four times the standard deviation from the mean, were removed from this analysis.

Supplementary Equation 3 Determination of the LOB with 80% CI using a parametric approach

μ_B: Mean of healthy control log2 ratios; σ_B: standard deviation of healthy control log2 ratios

$$LOB = \mu_B \pm 1.282\sigma_B$$

Segments with log2 ratios above the positive LOB or below the negative LOB were identified as gain or loss, respectively.

Details to machine learning model for tumor detection

Machine learning classifiers were build based on 134 samples of 50 CRC patients with clinically evident tumor burden and 63 samples of negative controls. In detail, negative controls consisted of 55 healthy controls and 8 samples collected from patients more than 6 weeks in remission.

For performance evaluation predictions of the test set with the best model of each iteration were stored for receiver operating characteristic (ROC) analysis. The ROC curve and its area under the curve (AUC) were calculated by averaging over the 100 individual curves. To combine the models obtained for the different feature sets, the meta-learner published by Peneder et al. in 2021 [5] was used.

We evaluated the performance of ML classifiers on the following feature sets: (i) Global fragmentation as described by the proportion of fragments with the following lengths: 100 to 150 bp, 160 to 180 bp, 180 to 220 bp, 250 to 320 bp, the ratio of 100 to 150 bp by 163 to 169 bp, and the ratio of 160 to 180 bp by 180 to 220 bp. Also the amplitude at 10 bp was included [5, 6]. (ii) Regional fragmentation in terms of read depth of short (90 to 150 bp) and long (151 to 220 bp) fragments, and log2 transformed S/L ratio in 639 5 Mb bins as well as z-scored and log2 transformed S/L ratio over complete chromosome arms (Supplementary Table 4) [5, 7].

In silico dilutions

For proof-of-principle of the developed LIFE-CNA workflow *in silico* dilutions of five samples from CRC patients at diagnosis with predicted tumor fractions of >10% were prepared in five healthy control samples with target tumor content of 10%, 5%,2.5%, 1% and 0.5%. Based on the ichorCNA predicted tumor content and the mapping rate of individual samples, first CRC patient samples and healthy control samples were downsampled to the respective target reads using Picard DownsampleSam (v.2.25) (Supplementary Table 5). Following downsampling, the bam files of CRC patients and healthy controls were merged using samtools merge (v.1.10).

Supplementary Data

Study cohort

Supplementary Table 1 Baseline characteristics of study participants

Characteristics	Healthy	CRC	Stage I	Stage II	Stage III	Stage IV
**Number of individuals**	**61**	**50**	**7**	**14**	**11**	**18**
**Number of plasma samples**						
Total	61	198	14	48	40	96
Median	1	2	2	2	2	2
Range	1	1-23	1-3	1-16	1-10	1-23
**Blood samples collected at diagnosis**	**NA**	**43**	**6**	**13**	**8**	**16**
**Blood samples collected during follow-up**	**NA**	**155**	**8**	**35**	**32**	**80**
Therapy	NA	54	1	NA	24	29
partial remission	NA	18	NA	2	NA	16
stable disease	NA	36	NA	12	NA	24
progressive disease	NA	10	NA	NA	NA	10
Remission	NA	37	7	21	8	1
Remission >6 weeks	NA	9	0	3	6	0
**Hotspot variant analysis**						
Blood samples collected at Diagnosis	NA	3	NA	NA	1	2
Blood samples collected during follow-up	NA	30	NA	10	1	19
**CEA**						
Healthy	55	NA	NA	NA	NA	NA
Diagnosis	NA	40	5	12	8	15
Follow-up	NA	151	8	32	32	79
**MRD**	**NA**	**33**	**6**	**12**	**6**	**9**
**Monitoring**	**NA**	**15**	**0**	**1**	**4**	**10**
**Follow-up time (months)**						
Median	NA	0.4	0.3	0.6	0.3	1.3
Range	NA	0-18.3	0-1.3	0-16	0-18.3	0-16.1
**Age at enrollment**						
Median	32	73	76	77.5	74	70
Range	20-73	39-88	46-84	50-84	48-88	39-79
**Sex**						
Female	28	21	5	5	3	8
Male	33	29	2	9	8	10

Study design

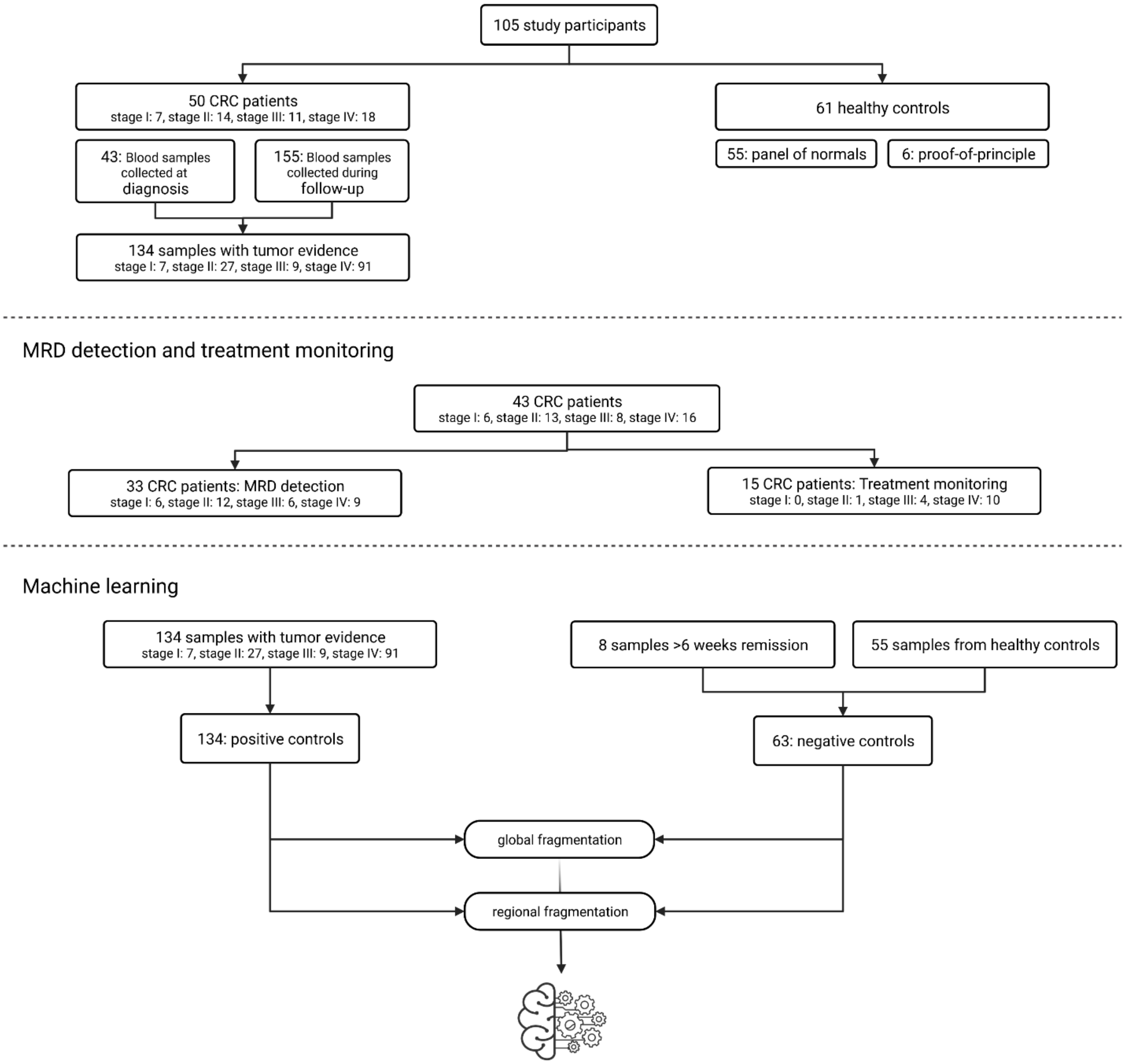

Supplementary Figure 1 Study design

Created with BioRender.com.

Coverage in CRC-specific active chromatin

Using the LIQUORICE tool [5], we analyzed the coverage in six CRC-specific active chromatin region sets. We compared samples collected from CRC patients at various time points of disease to healthy controls (n=55). Significantly stronger coverage drops could be observed for all region sets in samples collected from CRC patients at diagnosis, during therapy, with stable disease, and progressive disease. Further, significantly lower coverage was also observed in samples collected from patients in remission within the first six weeks post-surgery (Supplementary Figure 3).

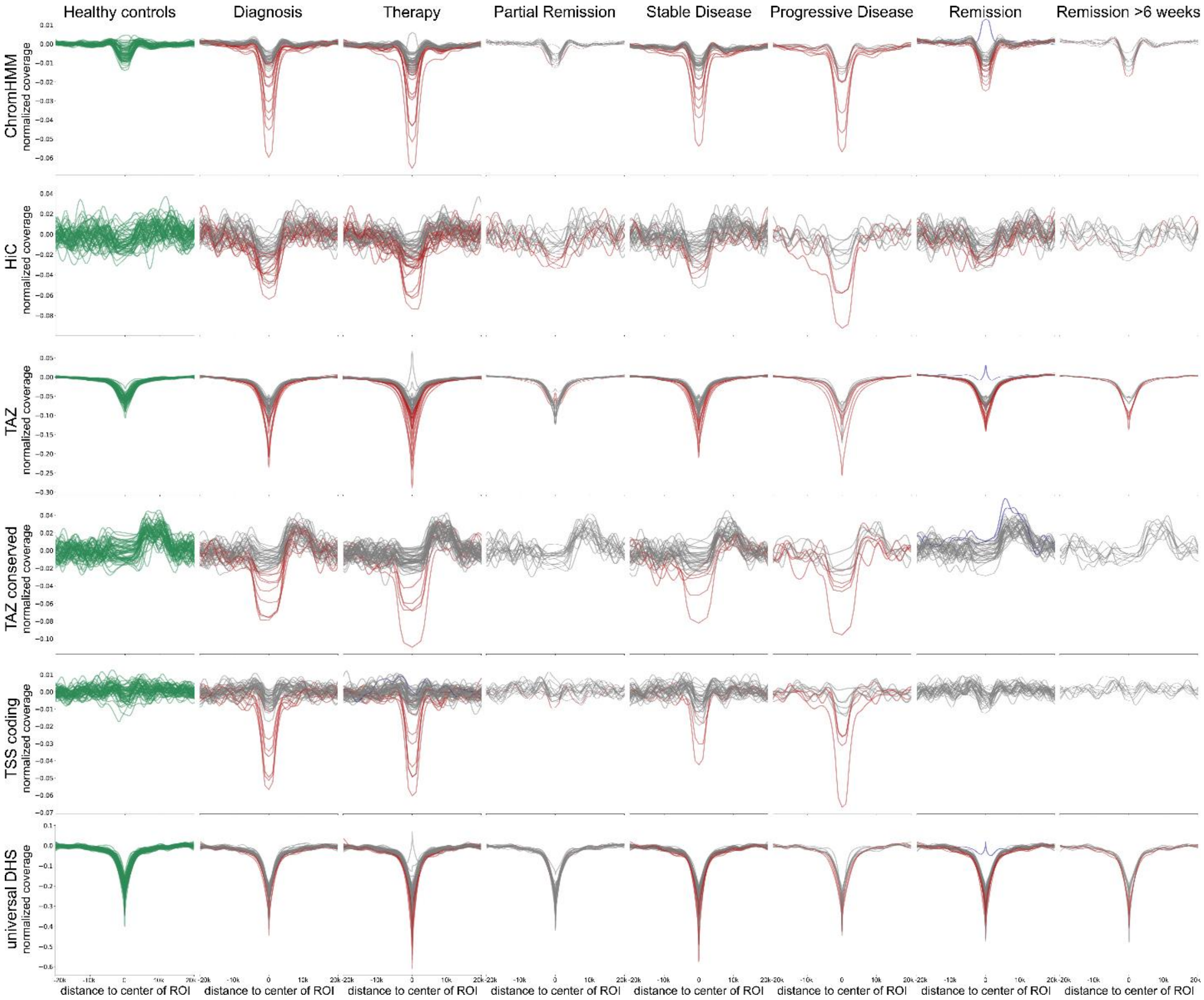

Supplementary Figure 2 Coverage drop in different region sets of interest

Coverage drops in six different region sets were analyzed in CRC patients collected at different time points during disease and in healthy controls (green). Significantly stronger coverage drops are shown in red, significantly weaker coverage drops are shown in green and coverage drops comparable to healthy controls are shown in grey.

Comparison of ML classifiers to hotspot assays

We further observed that the integrated classifiers detected ctDNA in all samples collected from patients with clinically evident tumor burden with high sensitivity. These finding could be supported by more sensitive predictions of ctDNA throughout treatment compared to targeted hotspot assays. While the classifier based on global fragmentation detected ctDNA in 12/30 samples, and the classifiers based on regional fragmentation and the meta-learner detected ctDNA in 100% (30/30) of samples, targeted hotspot assays detected ctDNA in only 13/30 samples from CRC patients with clinically evident tumor burden. However, in contrast to hotspot assays these classifiers are not informative for the detection of response or resistance to treatment as they only identify the presence of ctDNA but cannot support ctDNA quantification. (Figure 5, Supplementary Figures 6, 9, 10, 13, and 14).

SCNA-based tumor detection using ichorCNA is not suitable for ctDNA detection in CRC patients

To identify and quantify tumor content, we initially applied the ichorCNA tool [3]. We tested whether ichorCNA can reliably discriminate CRC patients across all stages from healthy controls based on tumor content estimation. To test specificity of this approach, we first analyzed 55 healthy controls. We observed false positive triploid genomes in 4 of the 55 healthy controls, leading to wrongly identified tumor content (Supplementary Figure 3). To ensure specificity of this approach, we calculated a cutoff for tumor content estimation with an α-error of 5% based on a non-parametric approach on healthy controls (Supplementary Methods). The cutoff for ichorCNA based tumor detection was 5.1%. Applying this cutoff, tumor could only be predicted in 27% (36/134) of samples from CRC patients with clinically diagnosed tumor burden. As size selection of 90-150 bp fragments leads to enrichment of the ctDNA fraction in cfDNA [6], the cutoff could be reduced to 4.6% and resulted in a slightly higher detection of ctDNA-positive tumor samples (35%, 47/134). Taken together, ichorCNA-based tumor detection is not suitable due to limited sensitivity across all CRC stages (Figure 5A&B).

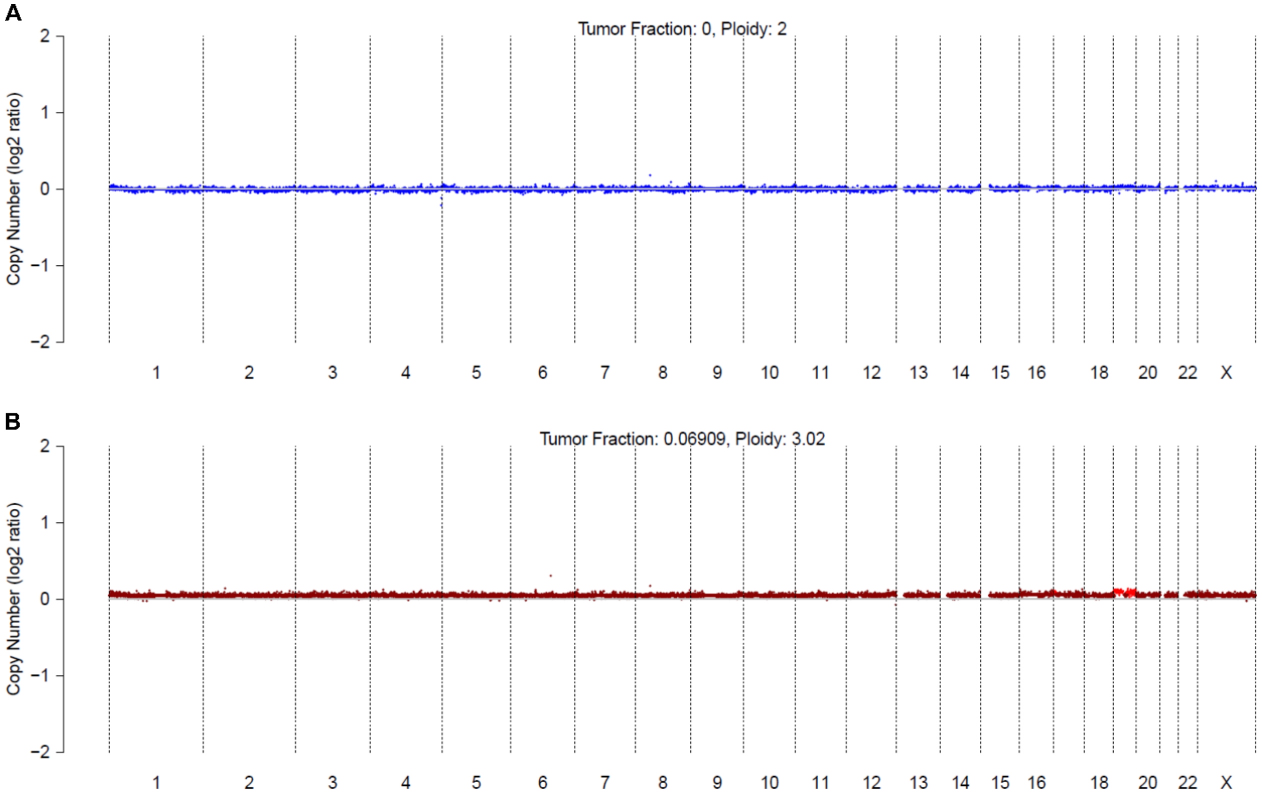

Supplementary Figure 3 Tumor fraction predicted with ichorCNA

ichorCNA tends to falsely predict triploid genomes and therefore tumor content in cfDNA based on SCNAs detected with shallow WGS. (A) Expected result for healthy control. (B) False positive detection of >5% tumor fraction in healthy control caused by prediction of triploid genome.

SCNA calling based on sex- and chromosome specific positive and negative LOBs

To ensure specificity of SCNA profiles in tumor samples, we calculated a cutoff for SCNA detection with a confidence interval of 80% based on a parametric approach on 55 healthy controls (Supplementary Methods, Supplementary Figure 3). We further, calculated a cutoff for presence of increased numbers of SCNAs in tumor compared to healthy controls with an α-error of 5% based on a non-parametric approach (Supplementary Methods). The cutoff for SCNA-based tumor detection were more than 7 SCNAs. Applying this cutoff, tumor could only be predicted in 47% (63/134) samples from CRC patients with clinically evident tumor. The cutoff for focal SCNA-based tumor detection were more than 2 focal SCNAs and resulted in a lower detection of ctDNA-positive tumor samples (25%, 34/134). However, at diagnosis we identified increased numbers of SCNAs in 36/43 patients (84%) in both, patients with localized and metastatic CRC (Figure 5A).

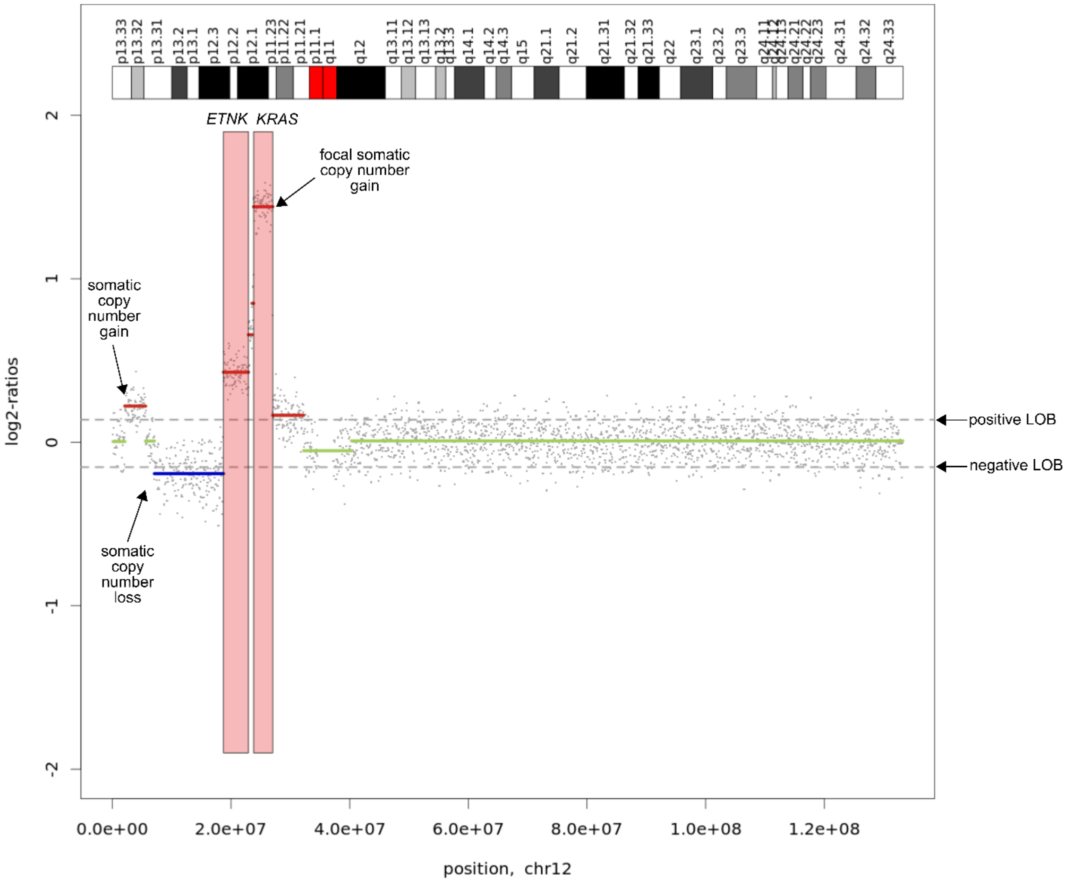

Supplementary Figure 4 SCNA calling based on sex- and chromosome specific positive and negative LOBs

The positive and negative LOB are calculated based on the distribution of log2 ratios in all analyzed bins on each chromosome. Segments above the positive LOB are identified as somatic copy number gains. Segments below the negative LOB are identified as somatic copy number loss. Gains or losses overlapping with the COSMIC cancer gene census [8] are identified as focal SCNAs. (dots: log ratios of each bin; lines: segments)

Genomic localization of SNCAs

Focusing on the localization of SCNAs present in plasma of our cohort, we detected SCNAs including focal SCNAs on all chromosomes, yet SCNAs located on chromosomes 3, 8, 16 and X, and focal SCNAs located on chromosomes 3, 8 and 13 were detected in most patients (Figure 4). Especially SCNAs on chromosomes 8 and 13 are frequently found in CRC [9].

LIFE-CNA bioinformatics analysis

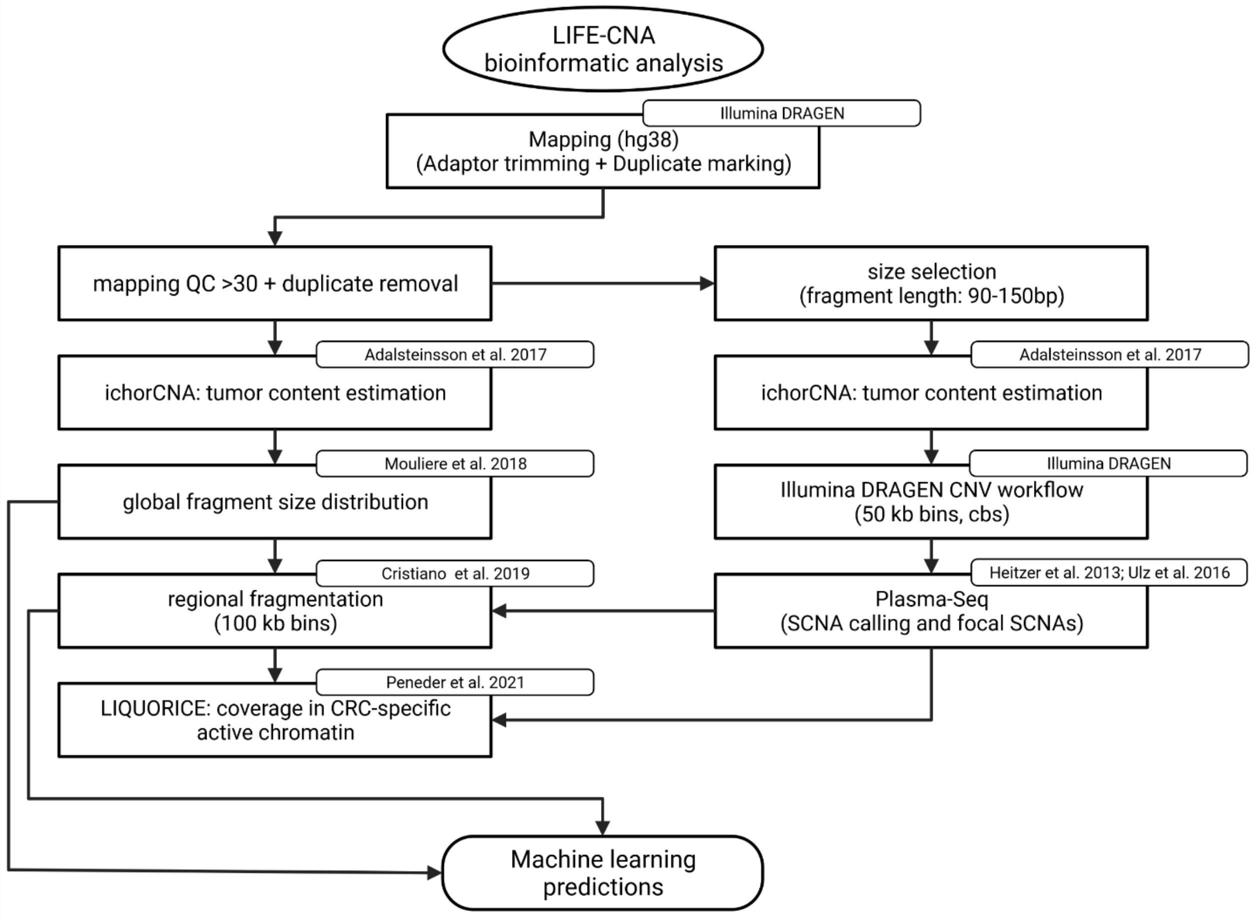

Supplementary Figure 5 LIFE-CNA bioinformatics analysis

Created with BioRender.com.

Disease monitoring

LB-CRC-07

Patient LB-CRC-07 was diagnosed with UICC stage II MSI CRC. *BRAF* p.V600E somatic variant was identified in tumor tissue. About eight months after R0 resection a singular liver metastasis was identified, which was surgically removed. Around seven months after surgical removal of the liver metastasis systemic nodal progression was diagnosed. Two weeks after diagnosis of systemic nodal progression chemotherapy was initiated. 1.5 months after initiation of chemotherapy partial remission was clinically confirmed. Another 4 months later stable disease was diagnosed. Throughout all following stagings disease remained stable and no progression was detected. Around 11 months after initiation of chemotherapy treatment was changed to checkpoint inhibitor therapy. Disease remained stable over the following 10 months, until the patient dropped out of the study, three years after initial diagnosis.

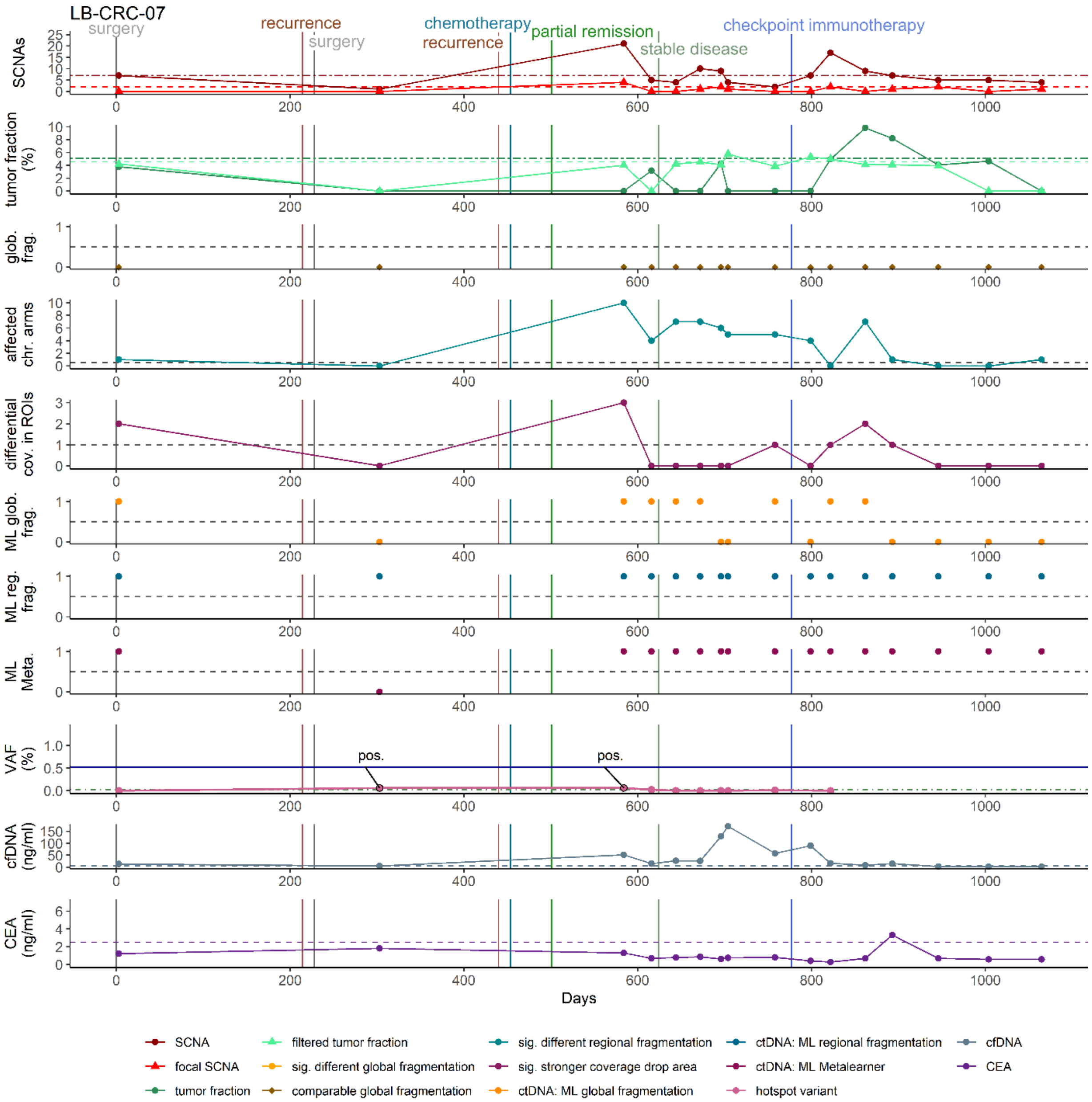

Supplementary Figure 6 Monitoring of cfDNA features analyzed with LIFE-CNA, mutant variant, CEA levels and cfDNA concentration in patient LB-CRC-07 stage IIA.

LB-CRC-08

Patient LB-CRC-08 was diagnosed with UICC stage IVC MSS CRC with metastasis in peritoneum and lung. *TP53* p.K321* somatic variant and *TBL1XR1*(Ex1)-*PIK3CA*(Ex2) gene fusion were identified in tumor tissue. 1.5 months following primary surgery the patient received adjuvant chemotherapy. Two months after initiation of adjuvant chemotherapy progressive disease was identified and treatment was changed. Two months later again progressive disease was detected and treatment was changed again. Following the identification of progressive disease 1.5 months later palliative chemotherapy was initiated. This change of treatment did not improve the patient's health and the patient died around one year after initial diagnosis.

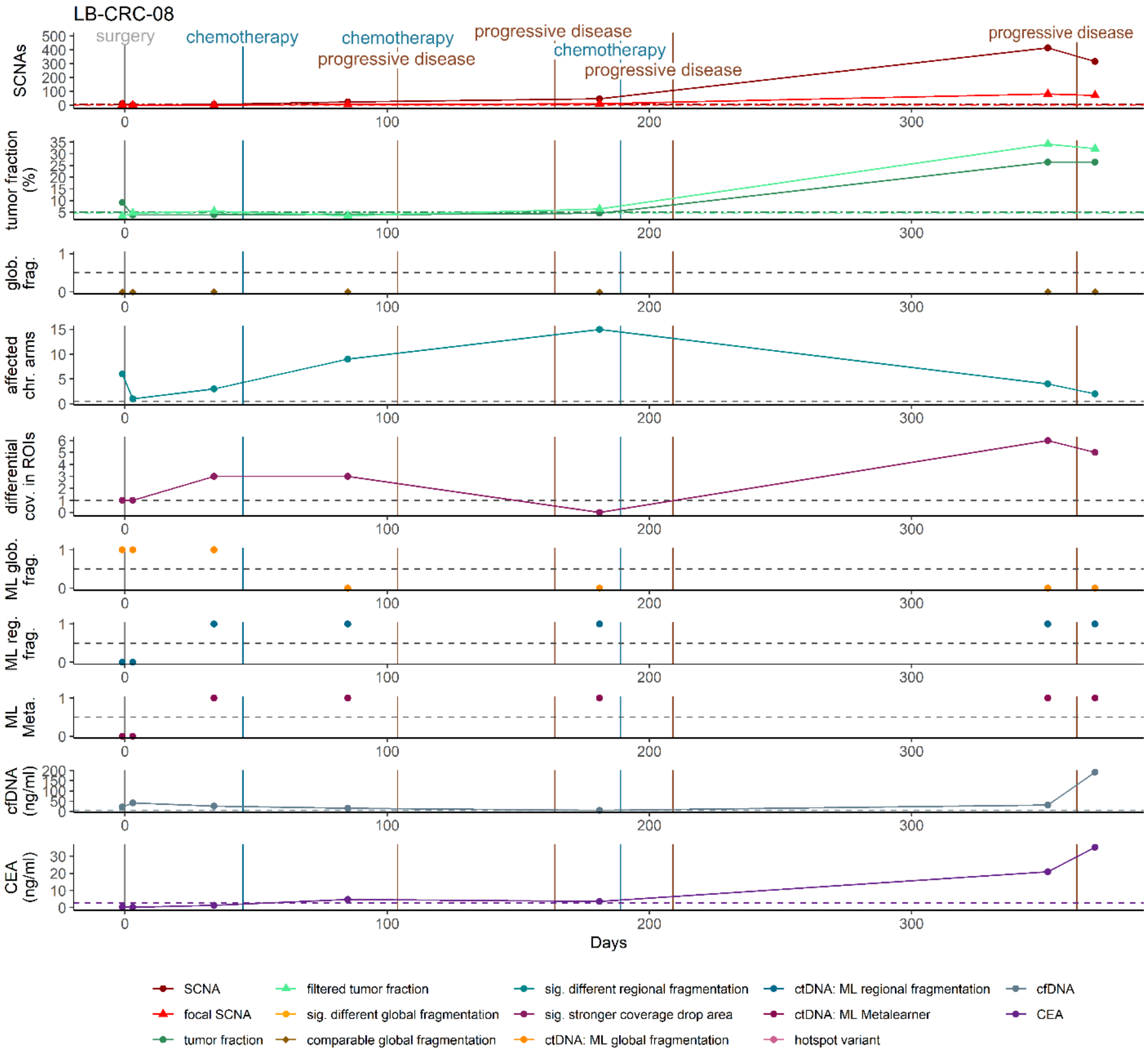

Supplementary Figure 7 *Monitoring of cfDNA features analyzed with LIFE-CNA, CEA levels and cfDNA concentration in patient* LB-CRC-08 stage IVC.

LB-CRC-21

Patient LB-CRC-21 was diagnosed with UICC stage IV CRC. The patient was treated with primary surgery and palliative chemotherapy.

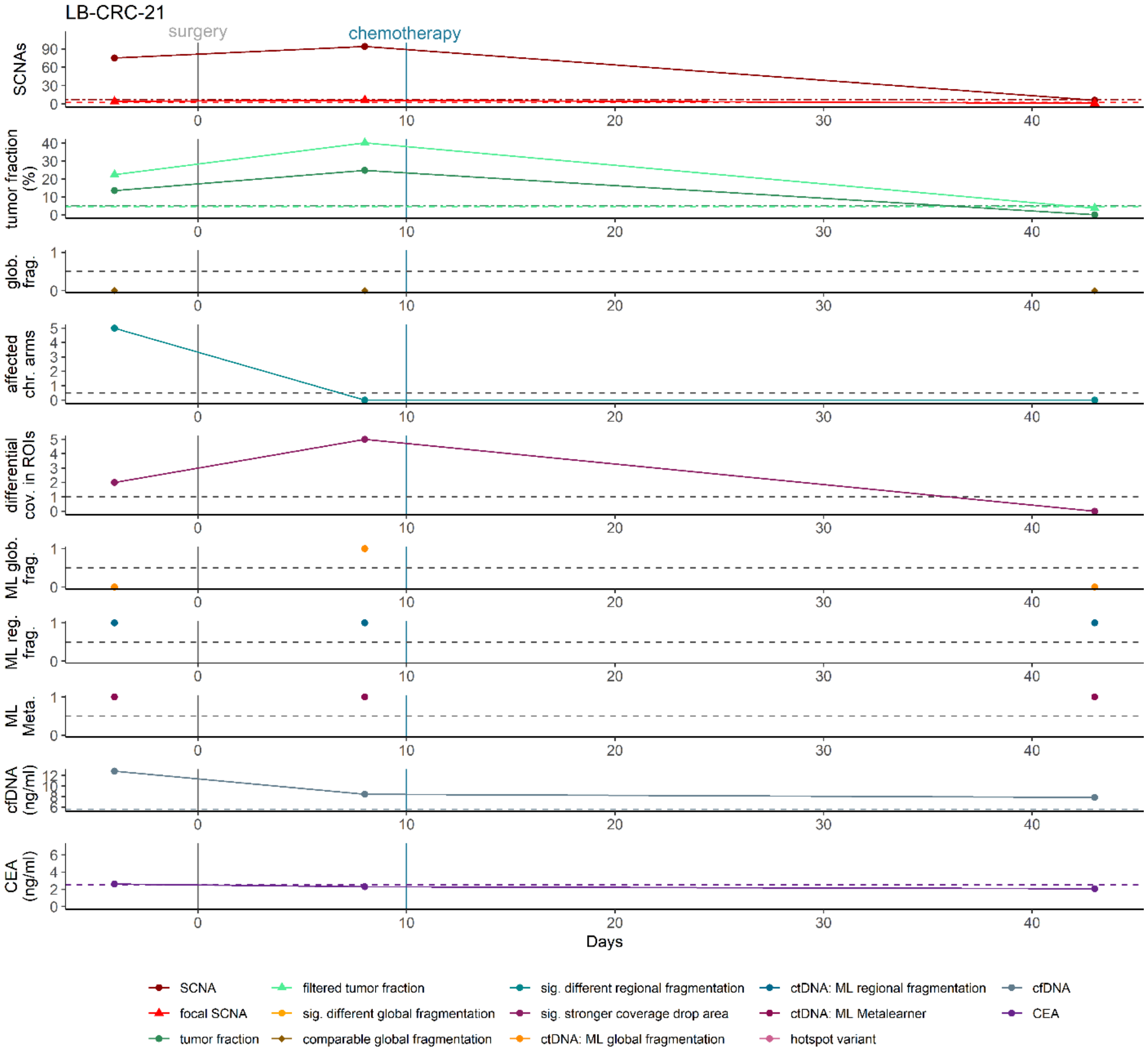

Supplementary Figure 8 Monitoring of cfDNA features analyzed with LIFE-CNA, CEA levels and cfDNA concentration in patient LB-CRC-21 stage IV.

LB-CRC-29

Patient LB-CRC-29 was diagnosed with UICC stage IIIB CRC. The patient was treated with primary surgery followed by adjuvant chemotherapy.

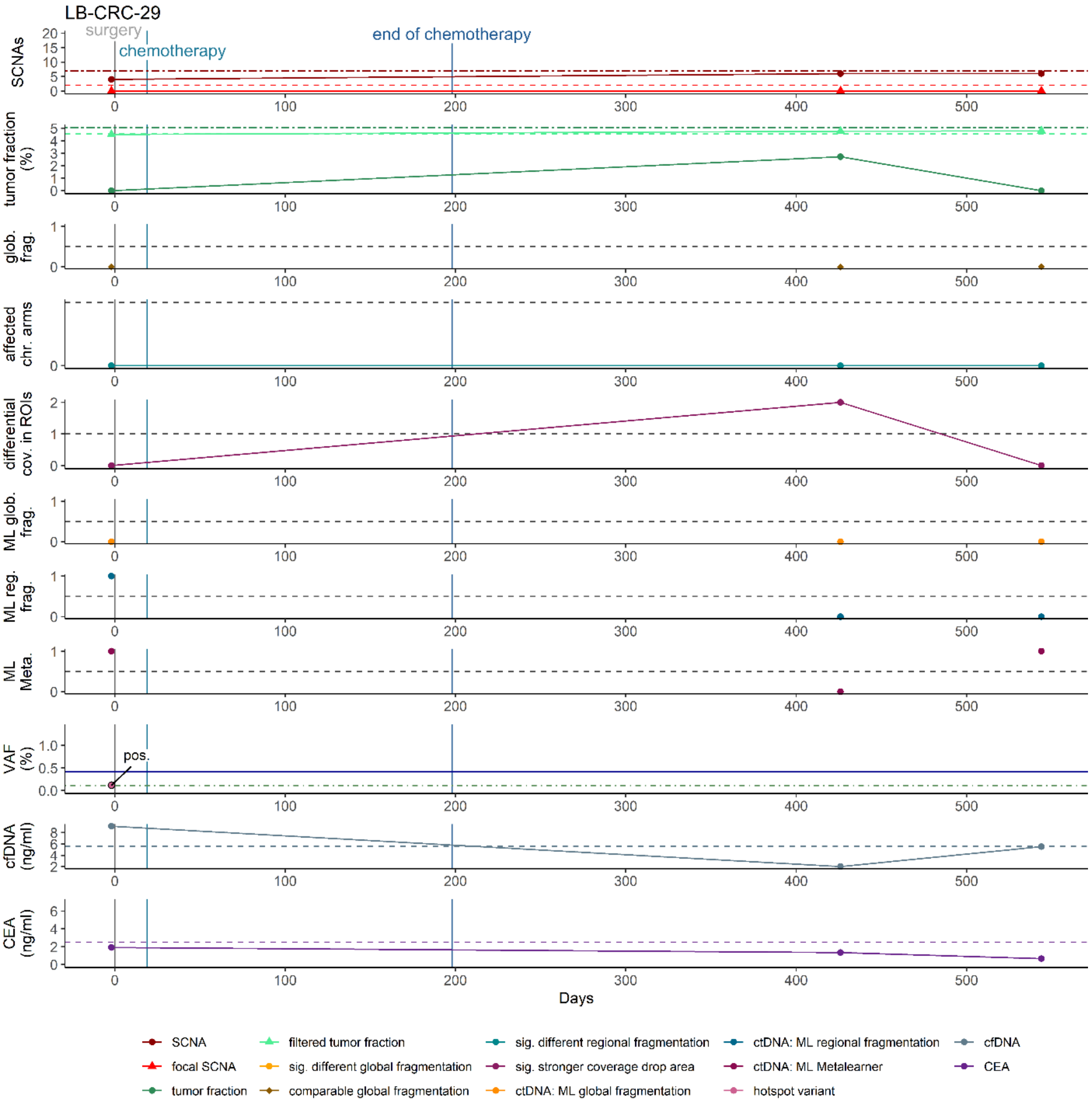

Supplementary Figure 9 Monitoring of cfDNA features analyzed with LIFE-CNA, mutant variant, CEA levels and cfDNA concentration in patient LB-CRC-29 stage IIIB.

LB-CRC-32

Patient LB-CRC-32 was diagnosed with UICC stage IV MSS CRC with liver metastasis. *BRAF* p.V600E somatic variant was identified in tumor tissue. Six months after initiation of palliative chemotherapy partial remission was identified. Around 4.5 months later the patient presented with stable disease. Another six months later progressive disease was detected.

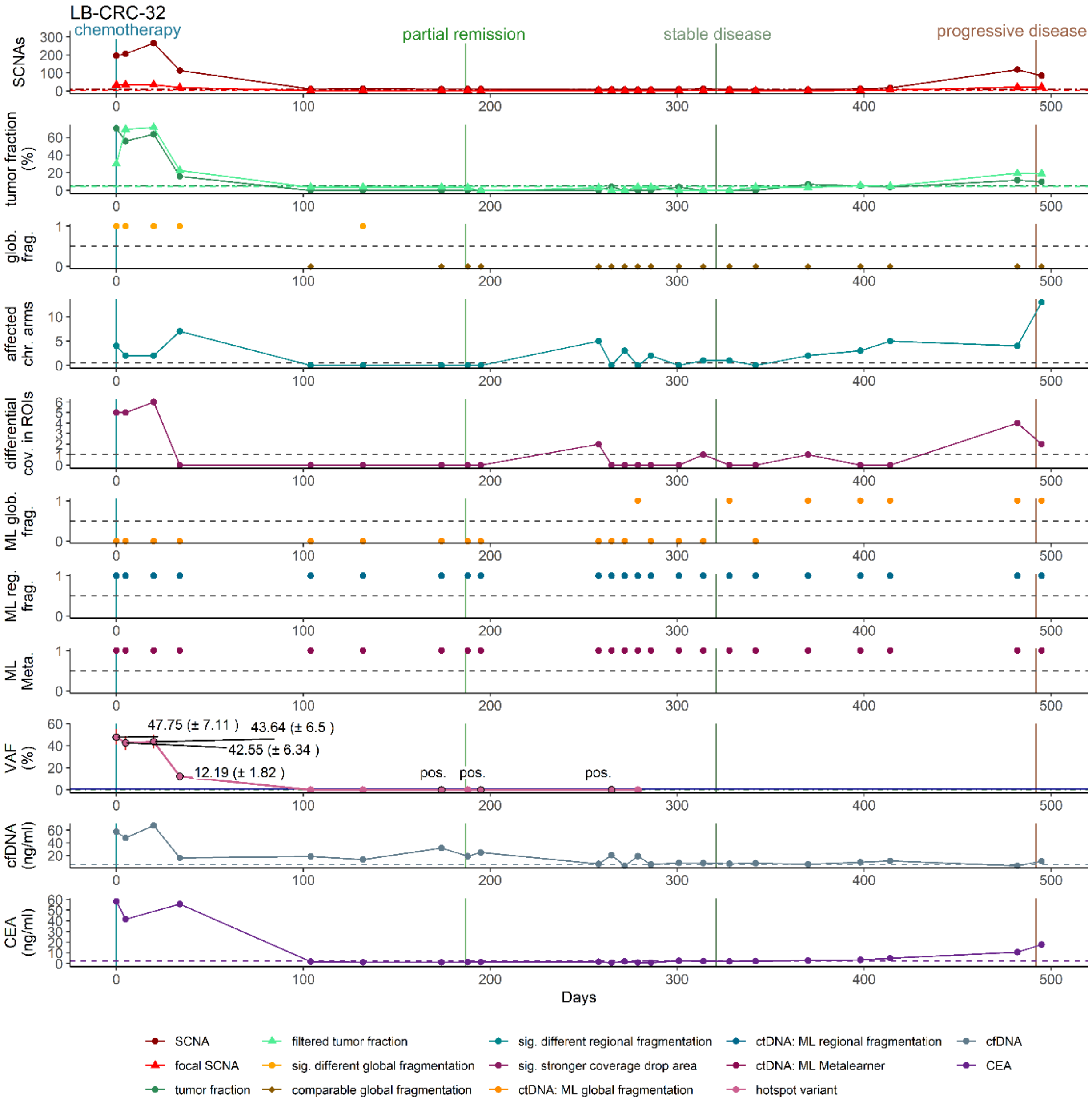

Supplementary Figure 10 Monitoring of cfDNA features analyzed with LIFE-CNA, CEA levels and cfDNA concentration in patient LB-CRC-32 stage IV.

LB-CRC-34

Patient LB-CRC-34 was diagnosed with UICC stage IV MSS CRC with metastasis in liver and lung. Two months after initiation of palliative chemotherapy partial remission was detected. Another three months later stable disease was identified and treatment was paused. After 1.5 months of paused treatment maintenance therapy was initiated. Two month later progressive disease was diagnosed and treatment was changed. This change of treatment did not improve the patient’s condition and the patient died around one year after initial diagnosis.

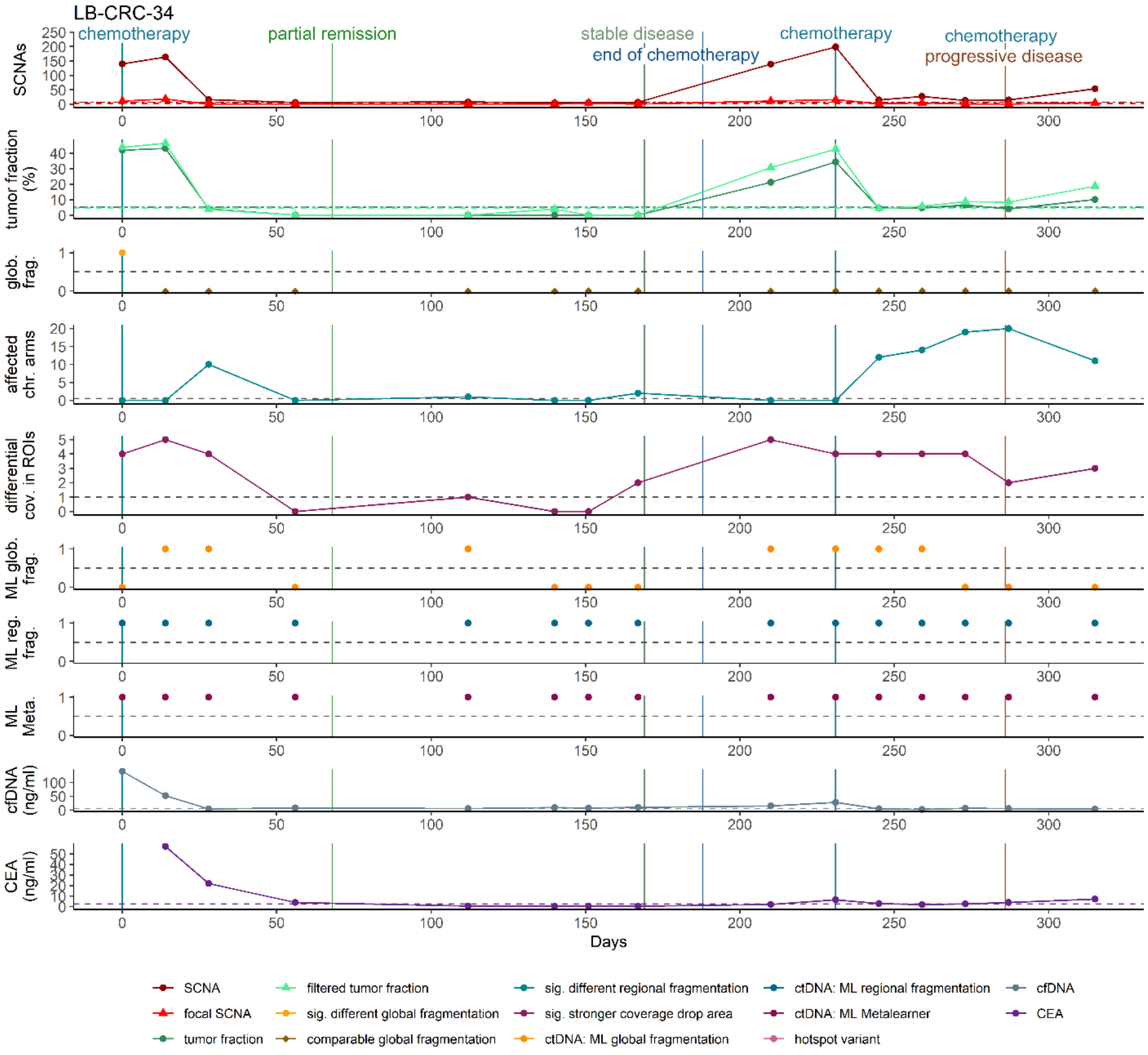

Supplementary Figure 11 Monitoring of cfDNA features analyzed with LIFE-CNA, CEA levels and cfDNA concentration in patient LB-CRC-34 stage IV.

LB-CRC-35

Patient LB-CRC-35 was diagnosed with UICC stage III MSS CRC. The patient was treated for two months with neoadjuvant radiochemotherapy. Two months after neoadjuvant treatment the patient received surgery. One month after surgery the patient was treated for two months with adjuvant radiochemotherapy. Following adjuvant chemotherapy no clinically evident tumor was observed over four months of follow-up.

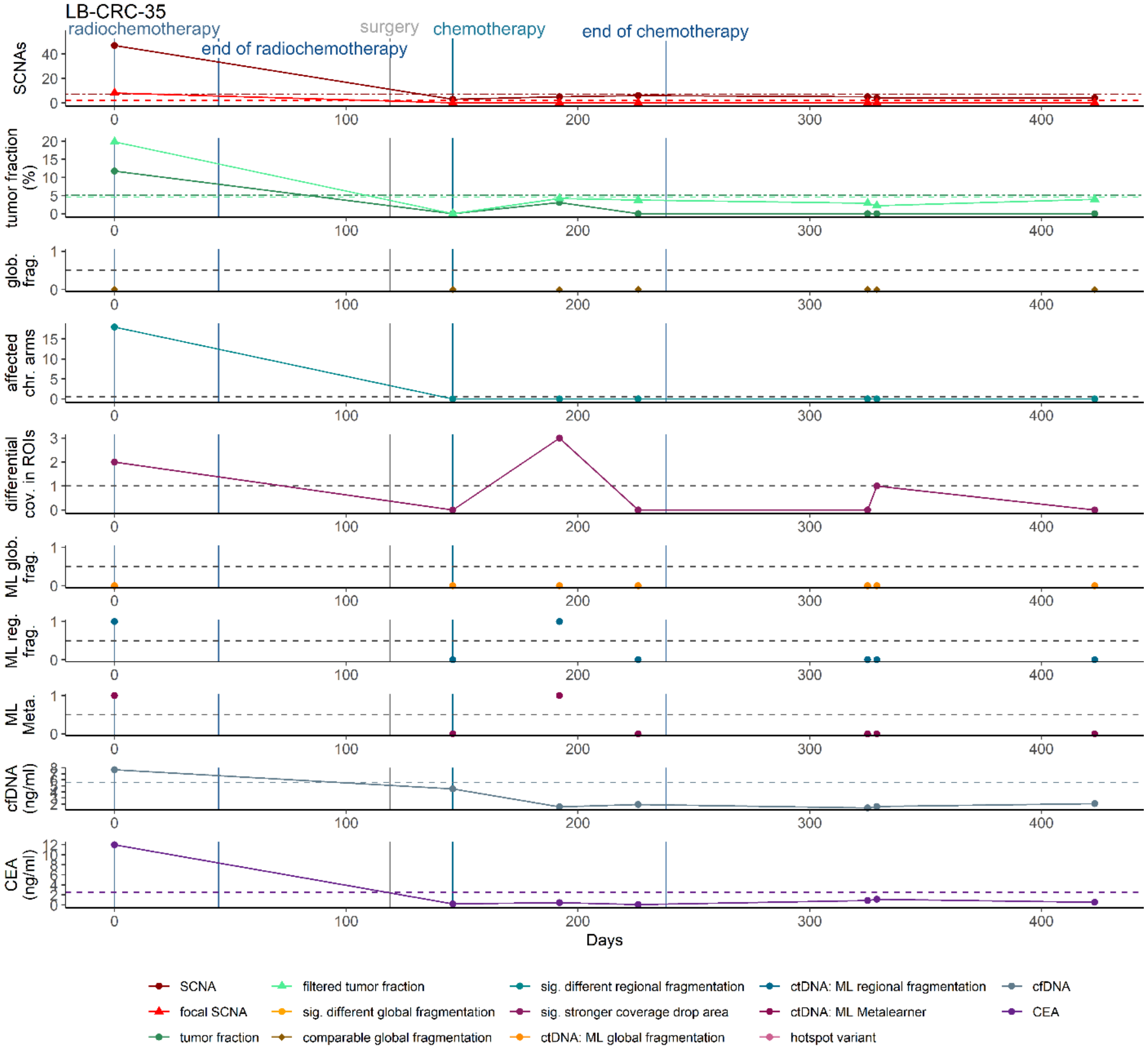

Supplementary Figure 12 Monitoring of cfDNA features analyzed with LIFE-CNA, CEA levels and cfDNA concentration in patient LB-CRC-35 stage III.

LB-CRC-38

Patient LB-CRC-38 was diagnosed with UICC stage IV CRC with peritonealcarcinosis. One month after primary surgery palliative chemotherapy was initiated. Three months after initiation of palliative chemotherapy stable disease was observed.

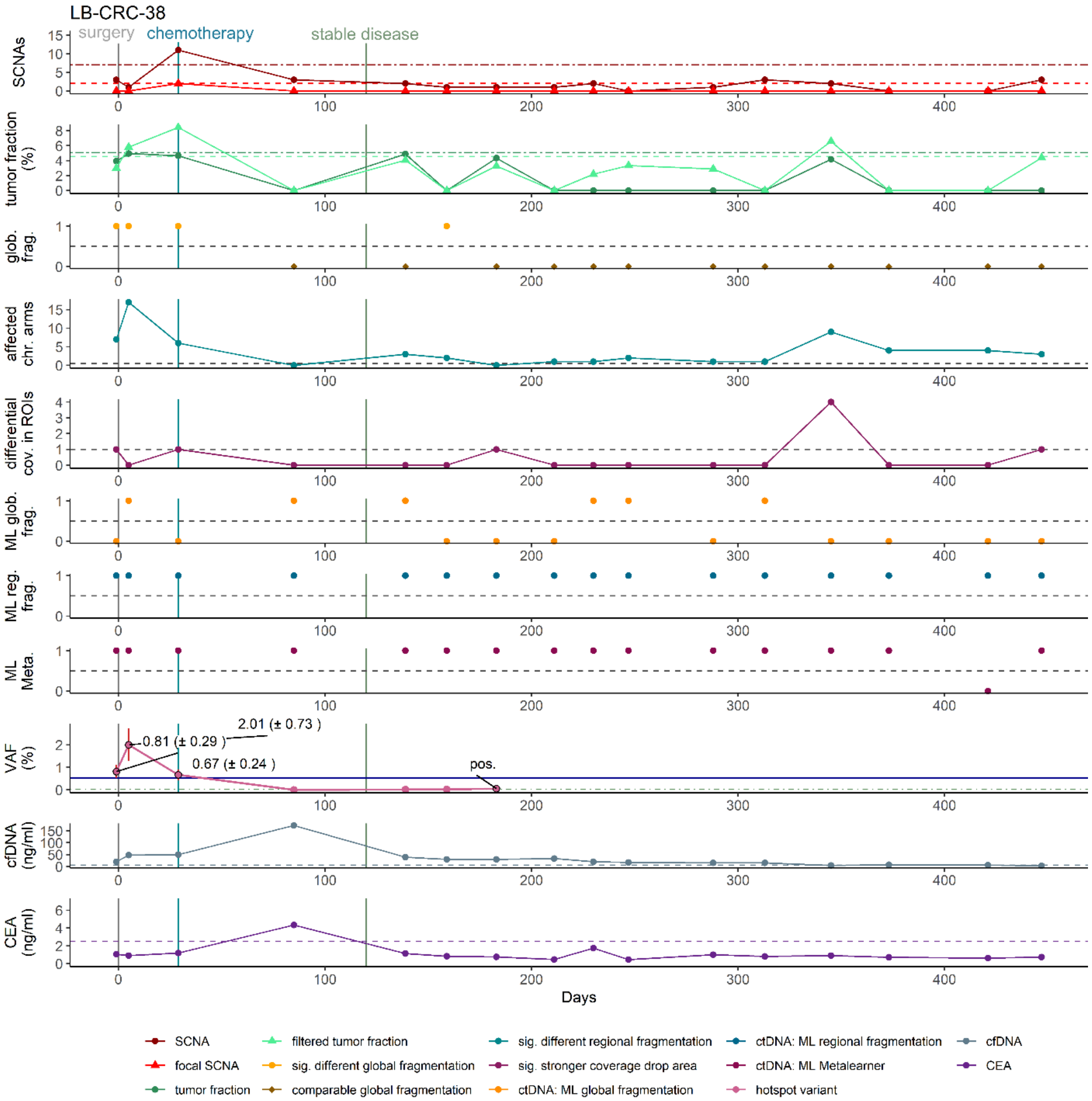

Supplementary Figure 13 Monitoring of cfDNA features analyzed with LIFE-CNA, mutant variant, CEA levels and cfDNA concentration in patient LB-CRC-38 IVC.

LB-CRC-43

Patient LB-CRC-43 was diagnosed with UICC stage IVA MSS CRC with metastasis in liver and lung. The patient was treated with initial palliative chemotherapy for two months. Following chemotherapy partial remission was identified. One month after completion of initial chemotherapy liver metastasis were resected followed by radiation of the colon carcinoma over one month. Following radiation the colon carcinoma was surgically removed. Around 4 months after rectum resection progressive disease was detected. Therefore the patient was treated with a second chemotherapy over four months. Two months after initiation of the second chemotherapy, stable disease was detected.

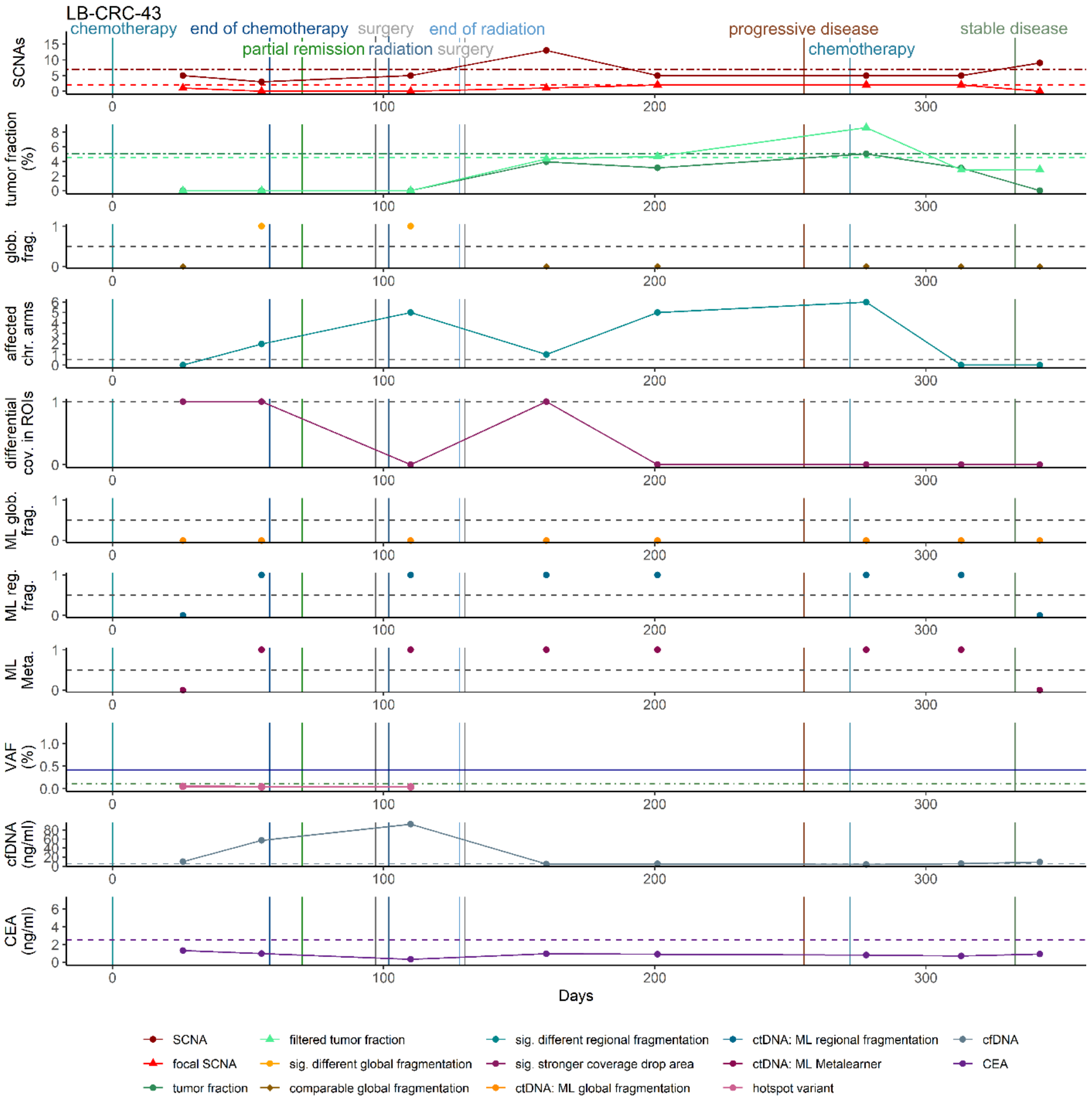

Supplementary Figure 14 Monitoring of cfDNA features analyzed with LIFE-CNA, mutant variant, CEA levels and cfDNA concentration in patient LB-CRC-43 stage IVA.

LB-CRC-47

Patient LB-CRC-47 was diagnosed with UICC stage III CRC. The patient was treated with primary surgery followed by adjuvant chemotherapy for five months around one month later. Following adjuvant chemotherapy the patient was in remission.

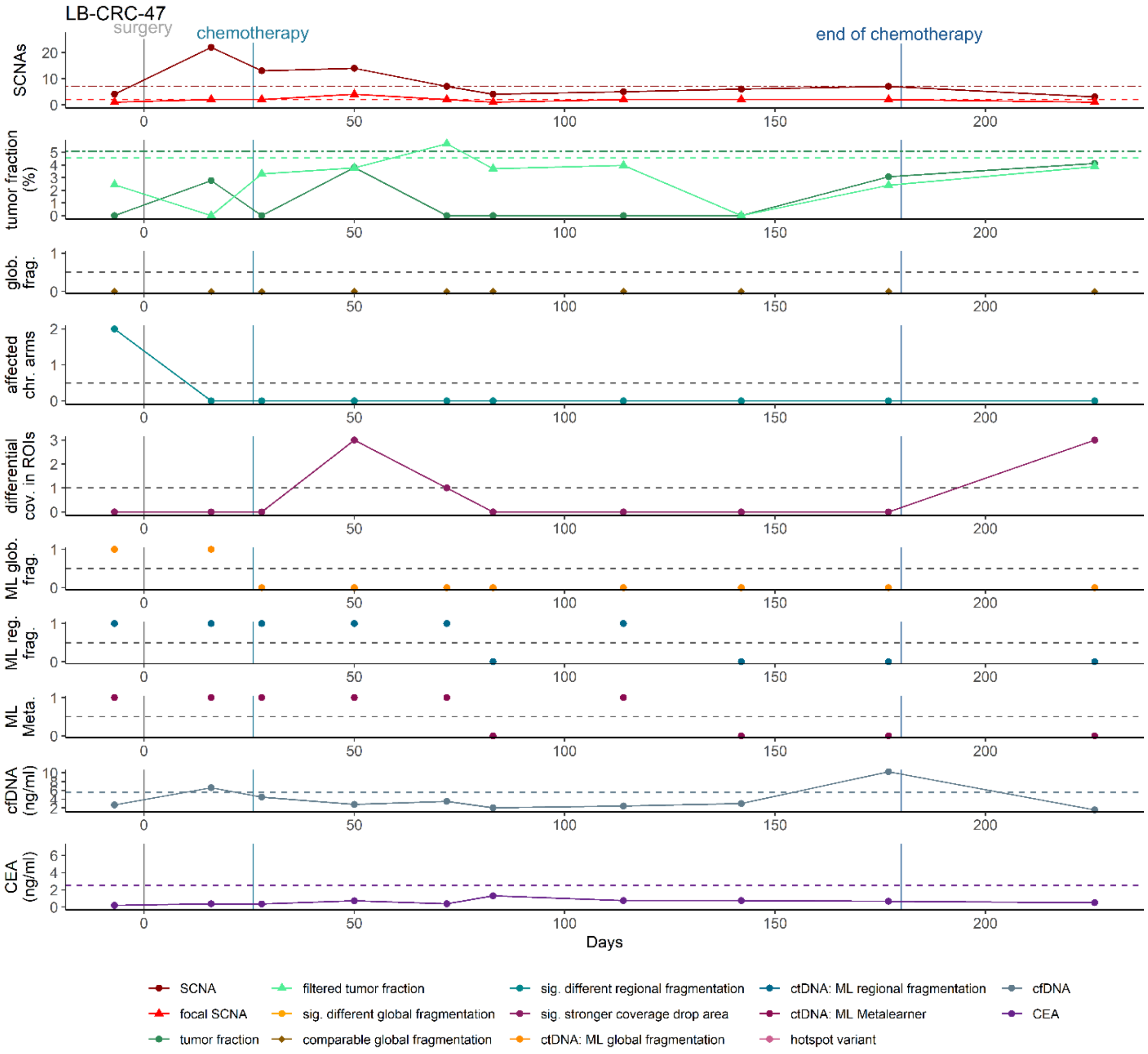

Supplementary Figure 15 Monitoring of cfDNA features analyzed with LIFE-CNA, CEA levels and cfDNA concentration in patient LB-CRC-47 stage III.

LB-CRC-48

Patient LB-CRC-48 was diagnosed with UICC stage IV MSI CRC with metastasis in liver and lung. The patient was treated with palliative chemotherapy. Already one week after initiation of chemotherapy progressive disease was identified and the patient received radiation for two weeks. Chemotherapy was discontinued two weeks after initiation. Following identification of still ongoing progressive disease one week after completion of radiation immunotherapy was initiated. The patient died one month later.

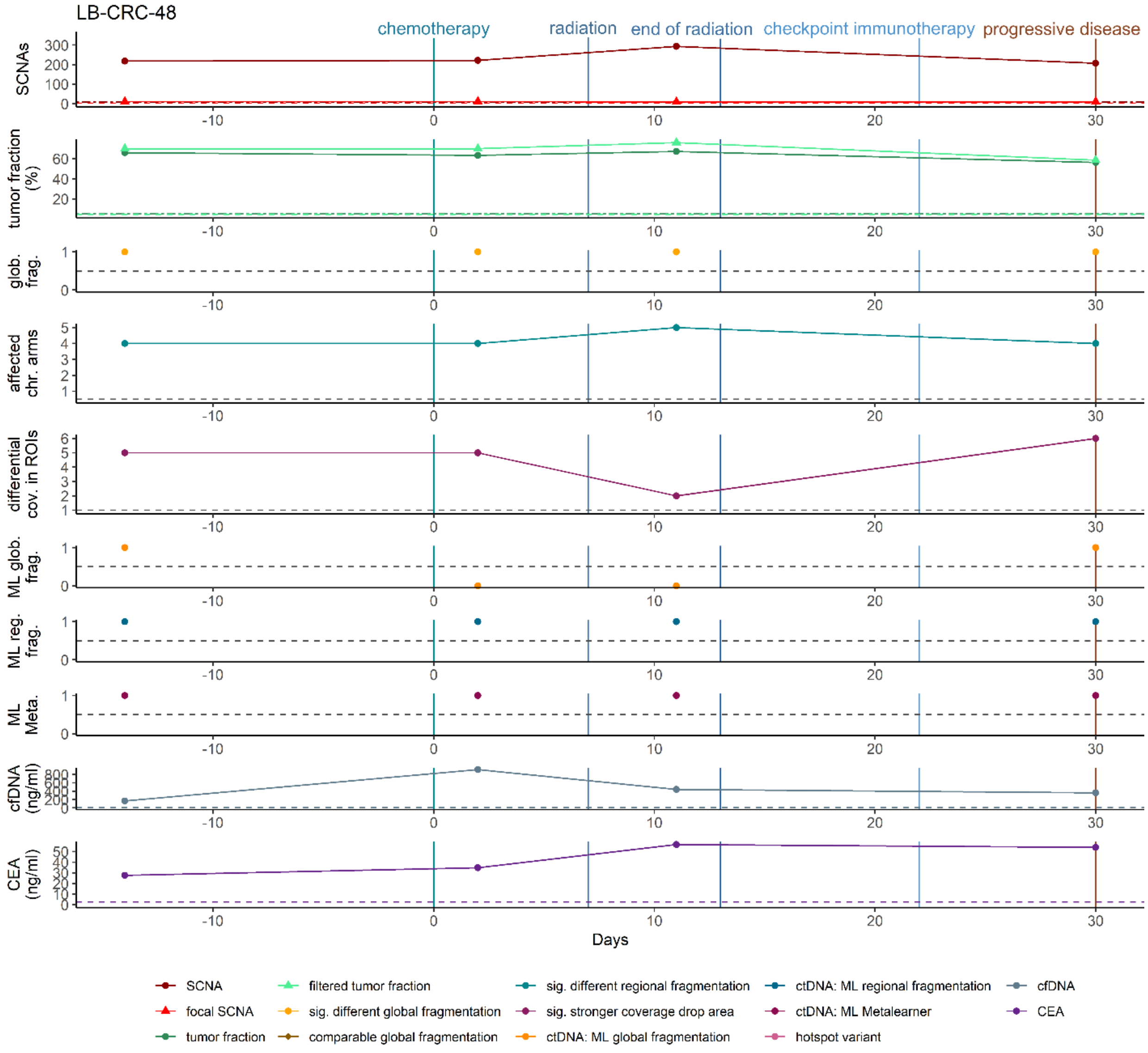

Supplementary Figure 16 Monitoring of cfDNA features analyzed with LIFE-CNA, CEA levels and cfDNA concentration in patient LB-CRC-48 stage IV.

LB-CRC-52

Patient LB-CRC-52 was diagnosed with UICC stage IIIC MSS CRC. The patient was treated with primary surgery followed by adjuvant chemotherapy for five months around one month later.

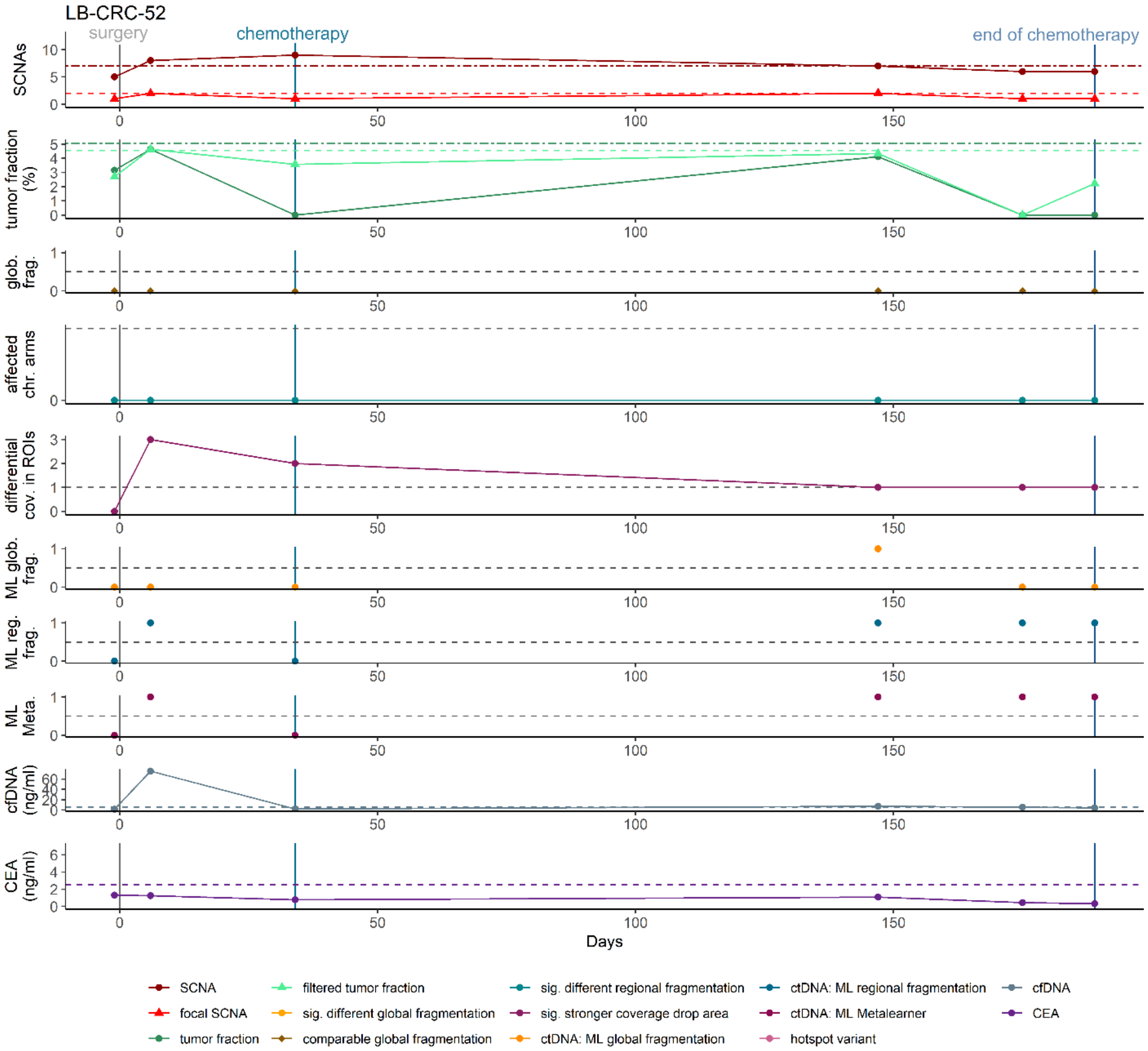

Supplementary Figure 17 Monitoring of cfDNA features analyzed with LIFE-CNA, CEA levels and cfDNA concentration in patient LB-CRC-52 stage IIIC.

LB-CRC-54

Patient LB-CRC-54 was diagnosed with UICC stage IVB MSS CRC. The patient was treated with chemotherapy.

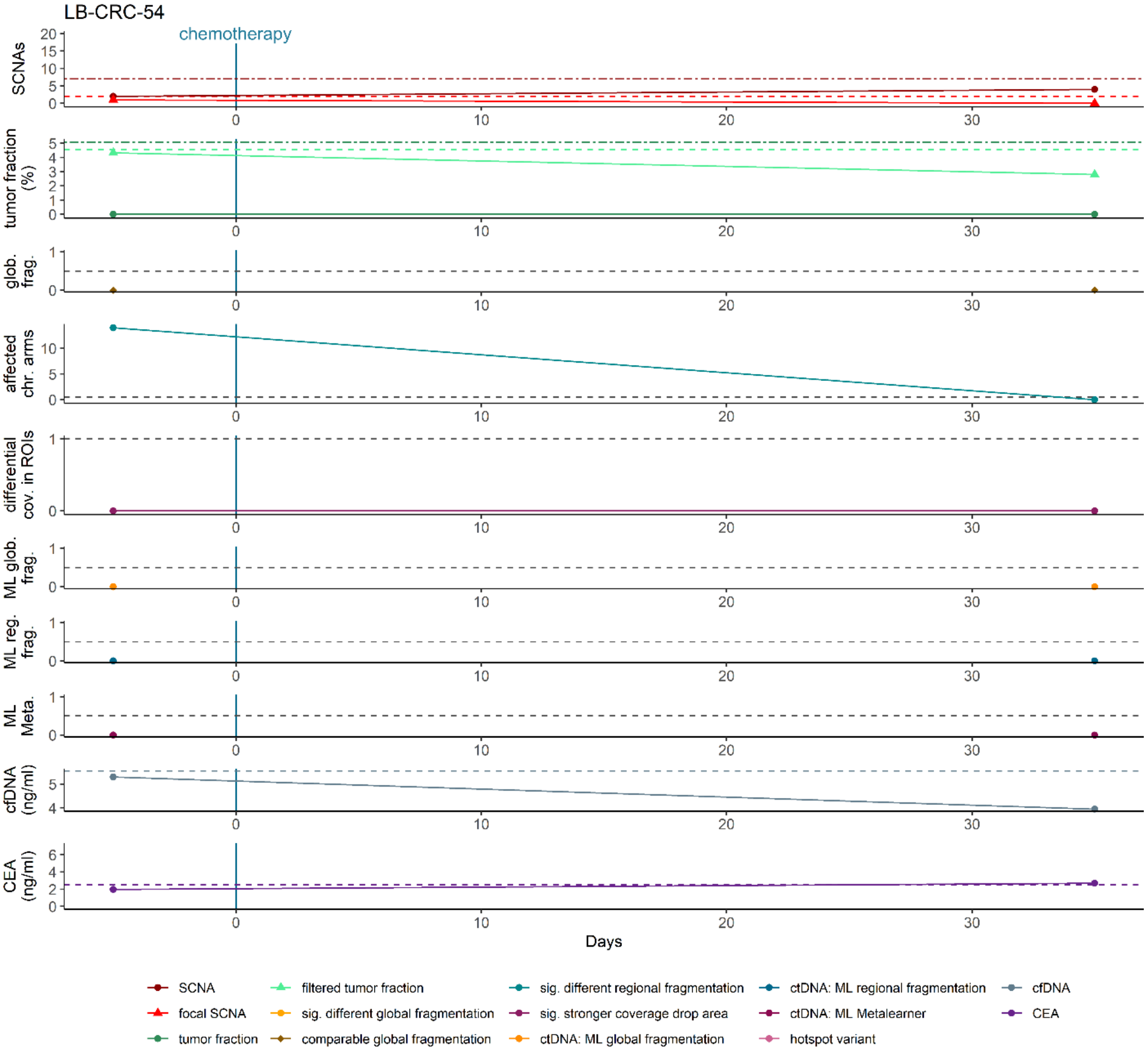

Supplementary Figure 18 Monitoring of cfDNA features analyzed with LIFE-CNA, CEA levels and cfDNA concentration in patient LB-CRC-54 stage IVB.

LB-CRC-59

Patient LB-CRC-59 was diagnosed with UICC stage IIIB CRC. The patient was treated with primary surgery. Around 2.5 months following surgery adjuvant chemotherapy was initiated.

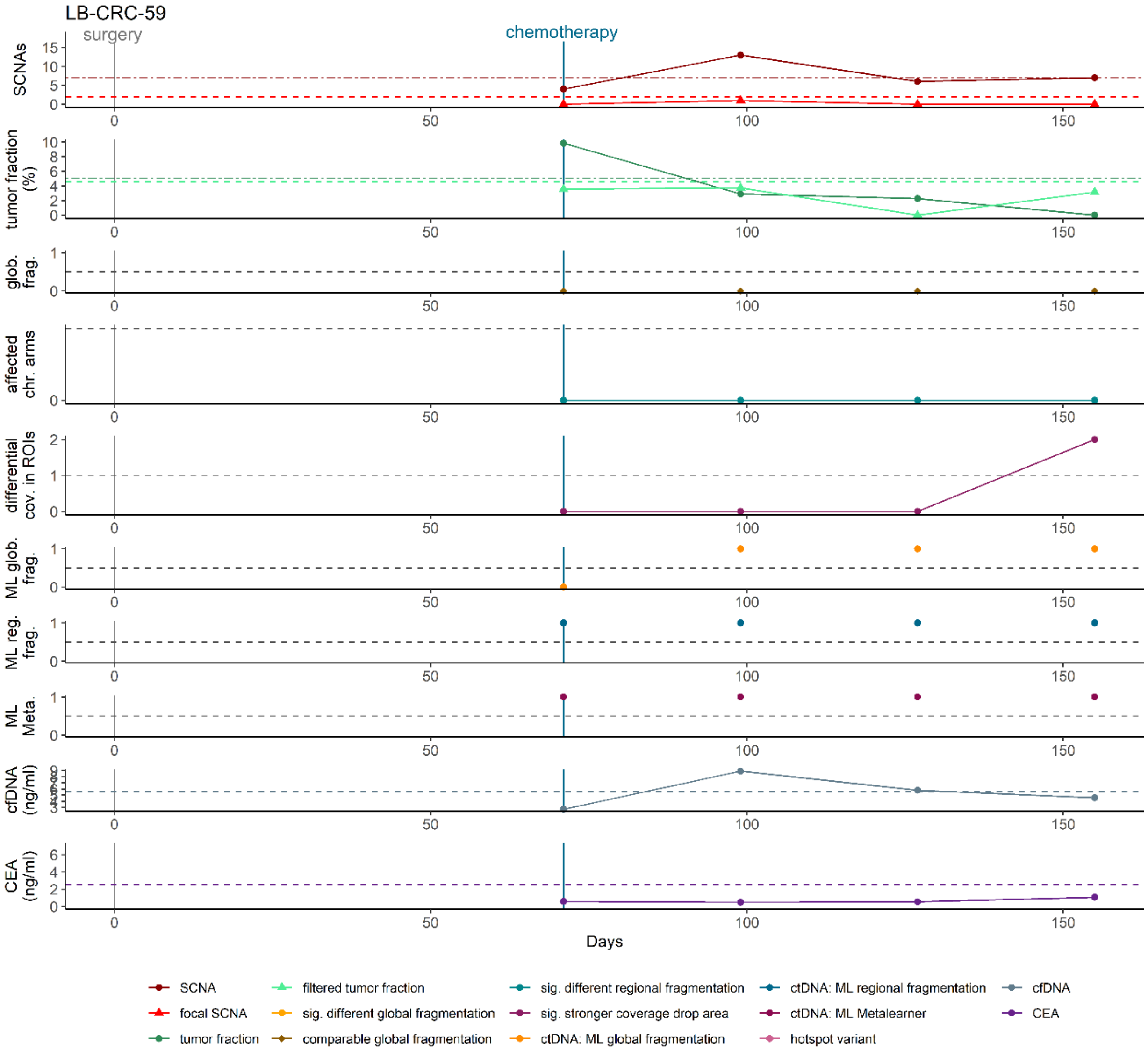

Supplementary Figure 19 Monitoring of cfDNA features analyzed with LIFE-CNA, CEA levels and cfDNA concentration in patient LB-CRC-59 stage IIIB.

LB-CRC-61

Patient LB-CRC-61 was diagnosed with UICC stage IV MSS CRC. The patient was treated with initial palliative chemotherapy.

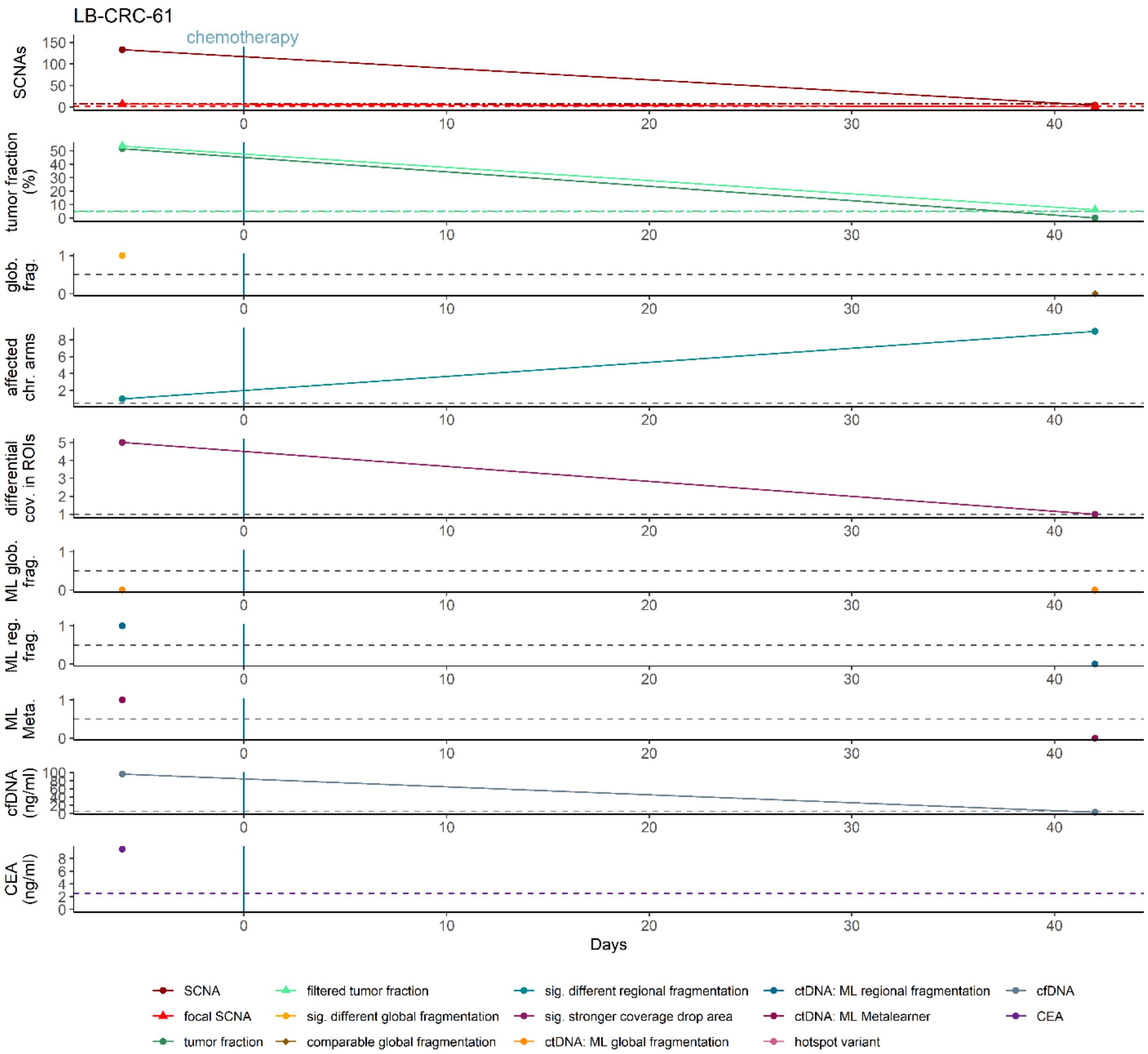

Supplementary Figure 20 Monitoring of cfDNA features analyzed with LIFE-CNA, CEA levels and cfDNA concentration in patient LB-CRC-61 stage IV.

Residual disease detection

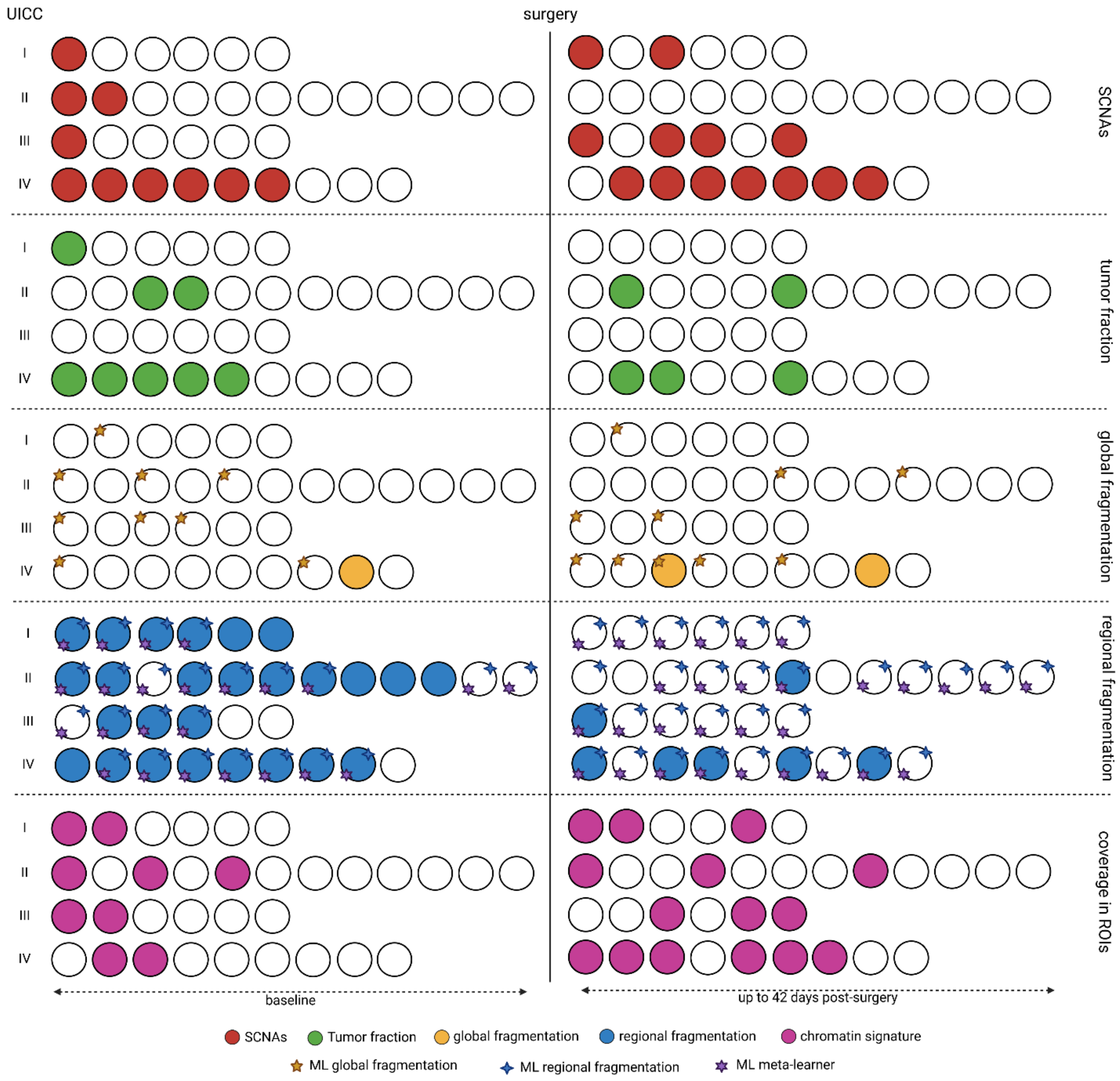

Supplementary Figure 21 Overview over all patients analyzed for MRD

Created with BioRender.com.

LB-CRC-01

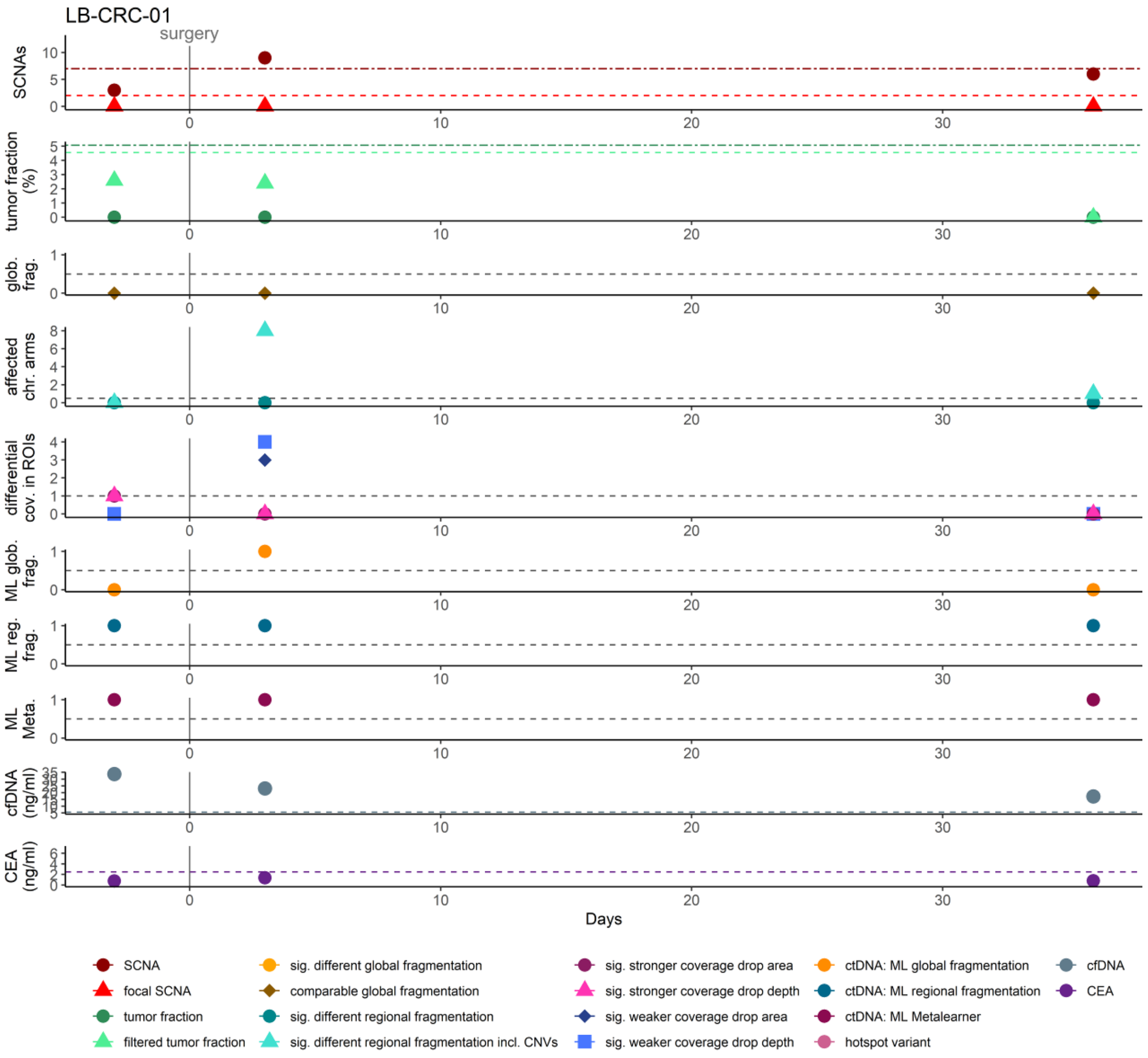

Supplementary Figure 22 Residual disease detection in patient LB-CRC-01.

Patient LB-CRC-01 was diagnosed with stage IIA CRC.

LB-CRC-08

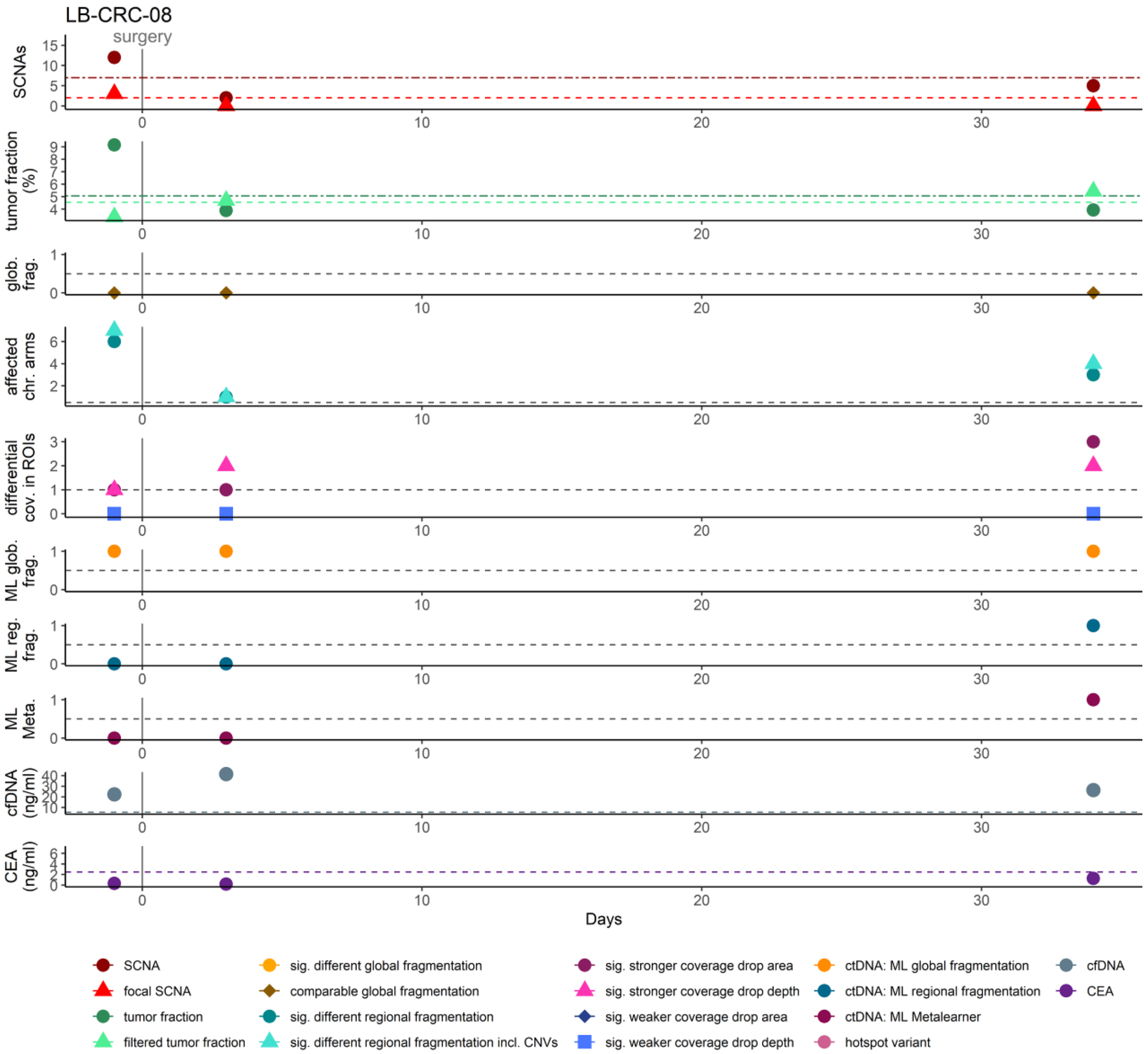

Supplementary Figure 23 Residual disease detection in patient LB-CRC-08.

Patient LB-CRC-08 was diagnosed with stage IVC CRC.

LB-CRC-13

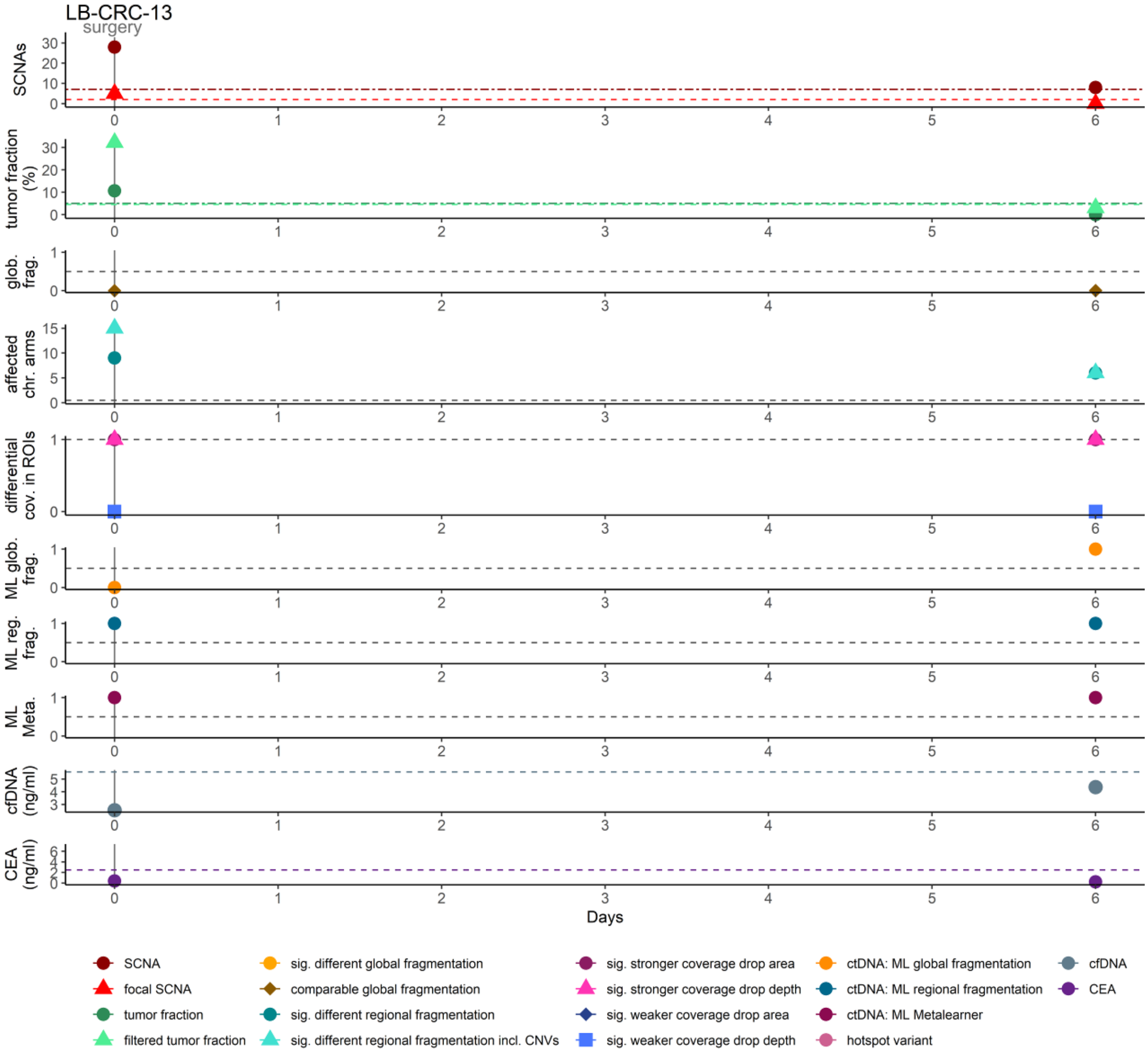

Supplementary Figure 24 Residual disease detection in patient LB-CRC-13.

Patient LB-CRC-13 was diagnosed with stage IVB CRC.

LB-CRC-15

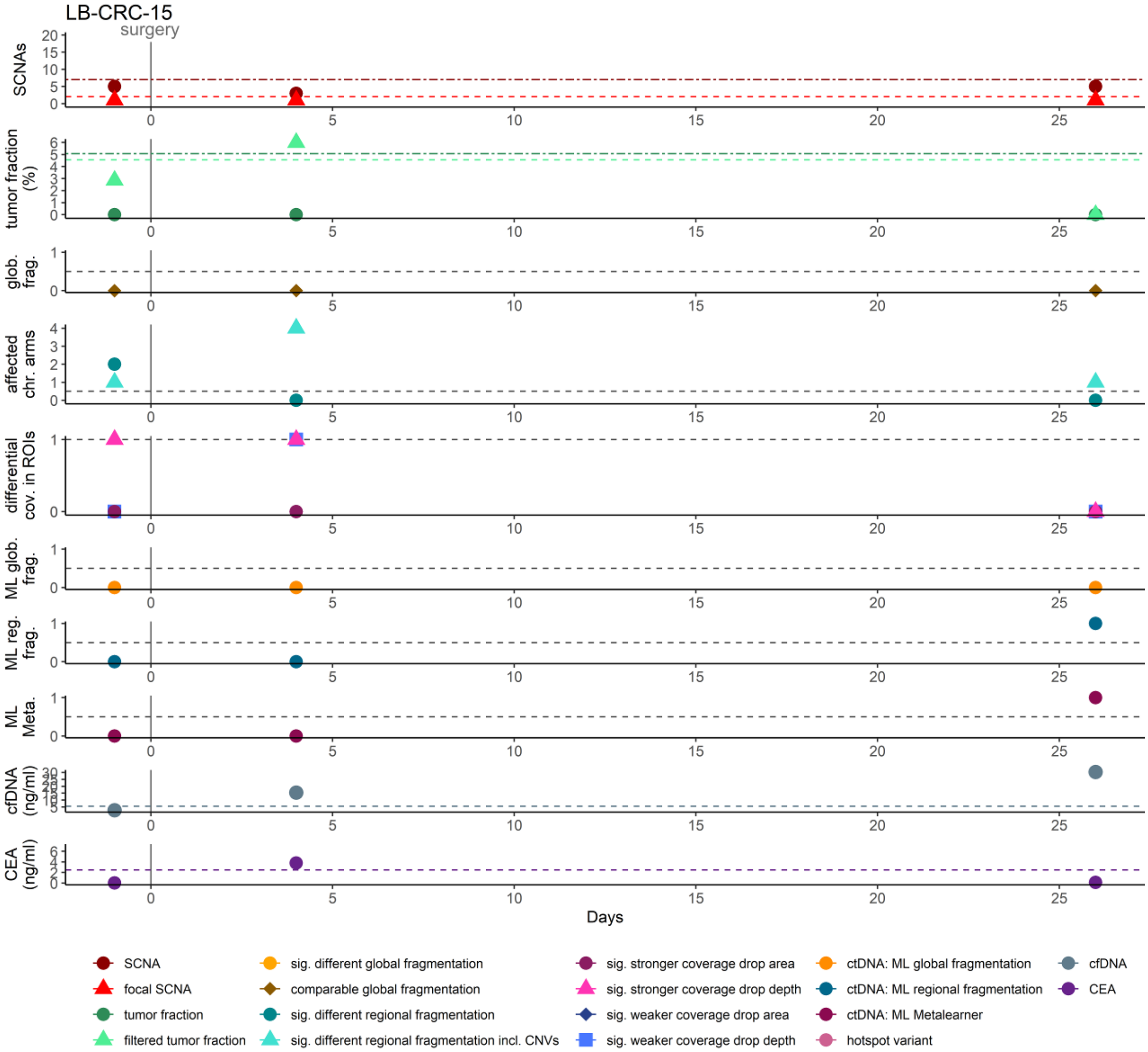

Supplementary Figure 25 Residual disease detection in patient LB-CRC-15.

Patient LB-CRC-15 was diagnosed with stage IIA CRC.

LB-CRC-17

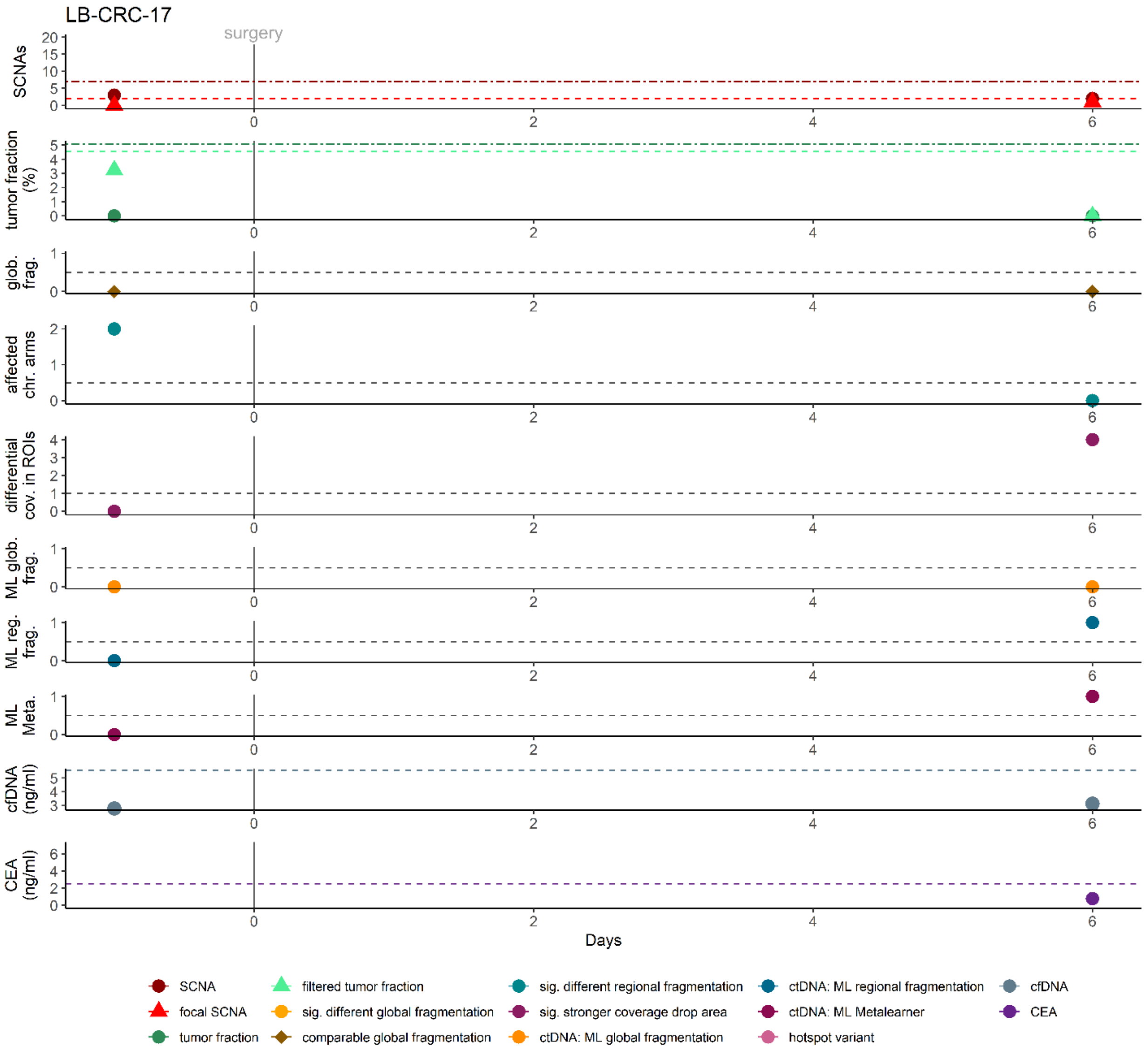

Supplementary Figure 26 Residual disease detection in patient LB-CRC-17.

Patient LB-CRC-17 was diagnosed with stage I CRC.

LB-CRC-20

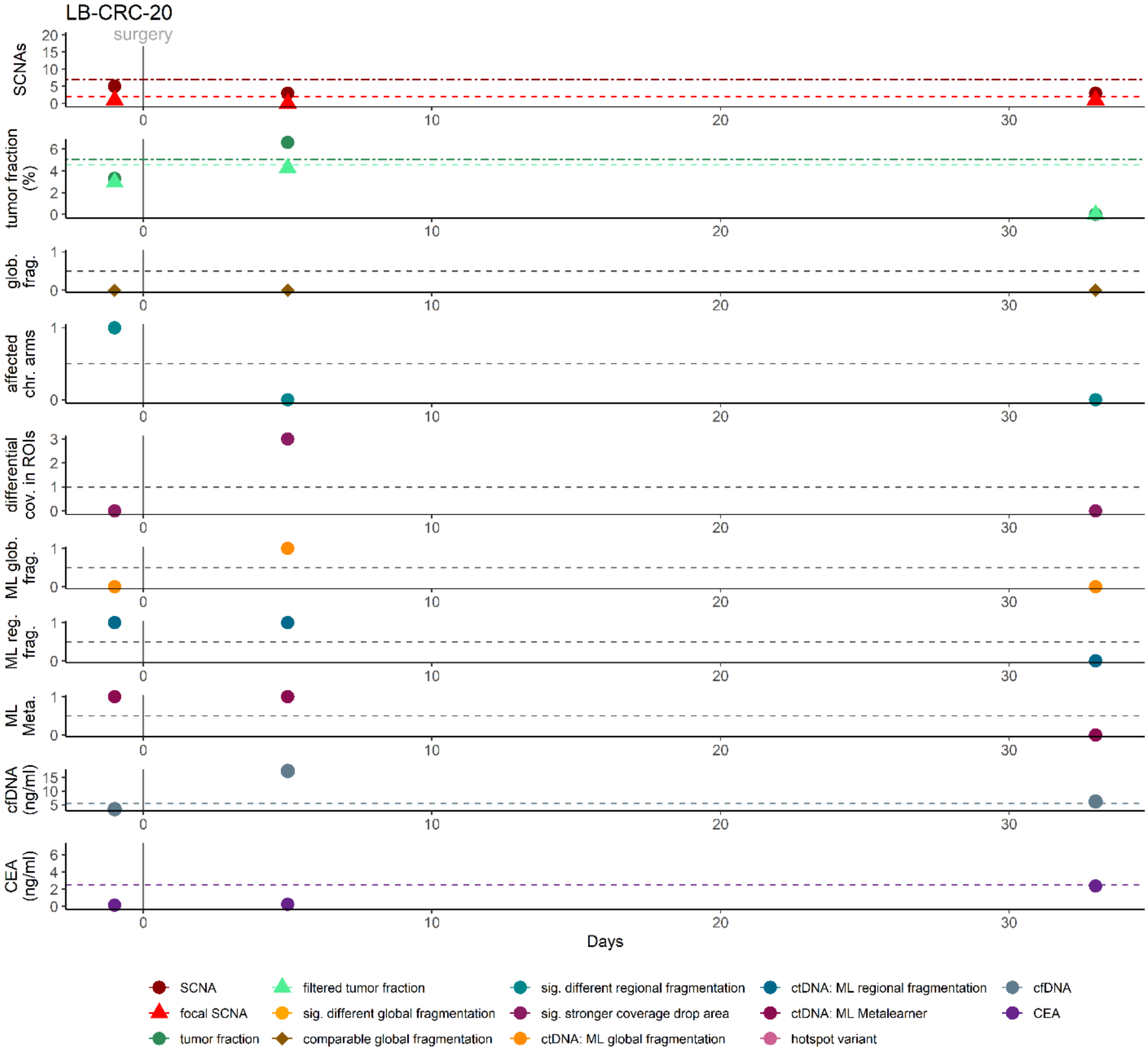

Supplementary Figure 27 Residual disease detection in patient LB-CRC-20.

Patient LB-CRC-20 was diagnosed with stage IIA CRC.

LB-CRC-21

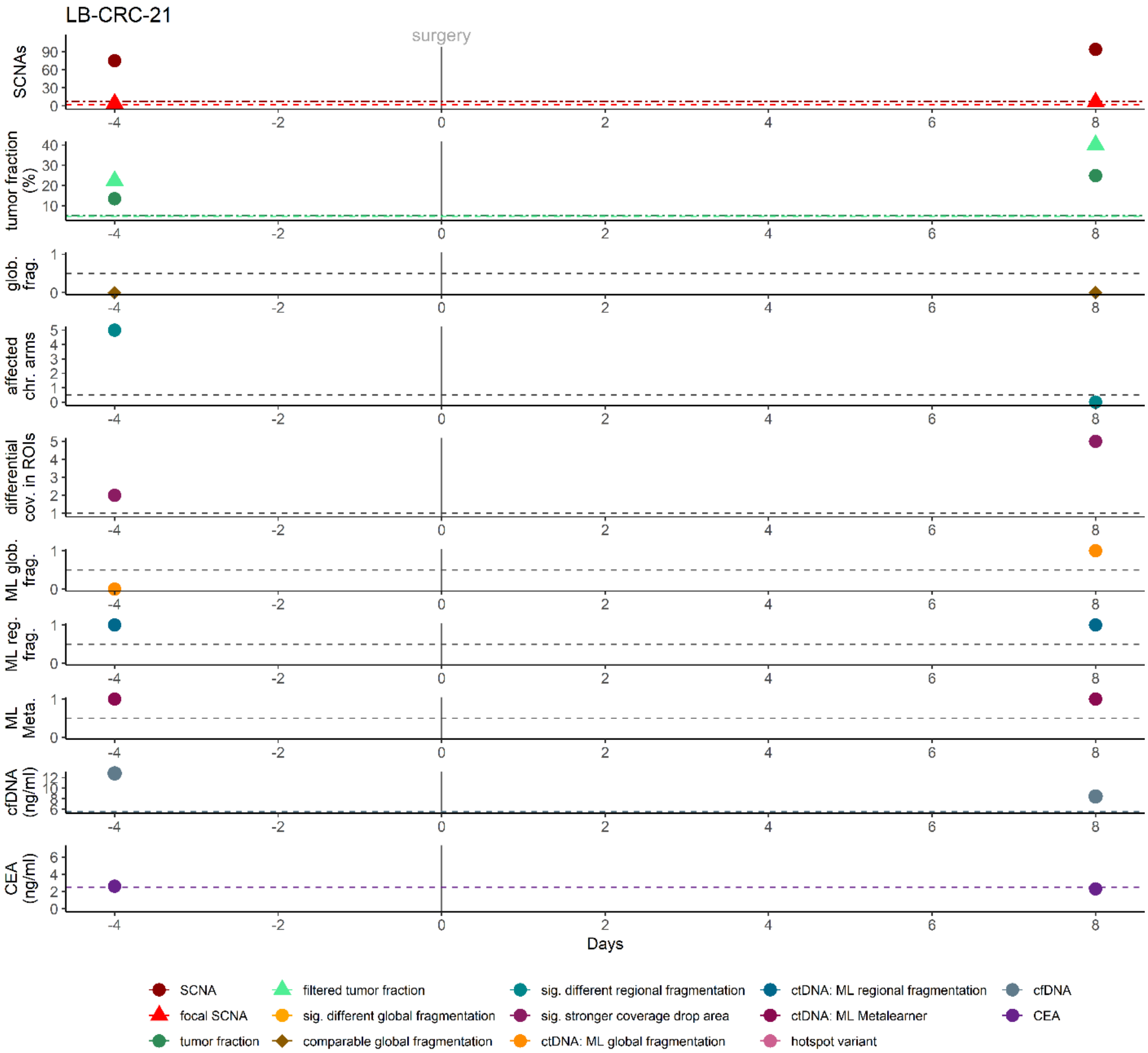

Supplementary Figure 28 Residual disease detection in patient LB-CRC-21.

Patient LB-CRC-21 was diagnosed with stage IV CRC.

LB-CRC-22

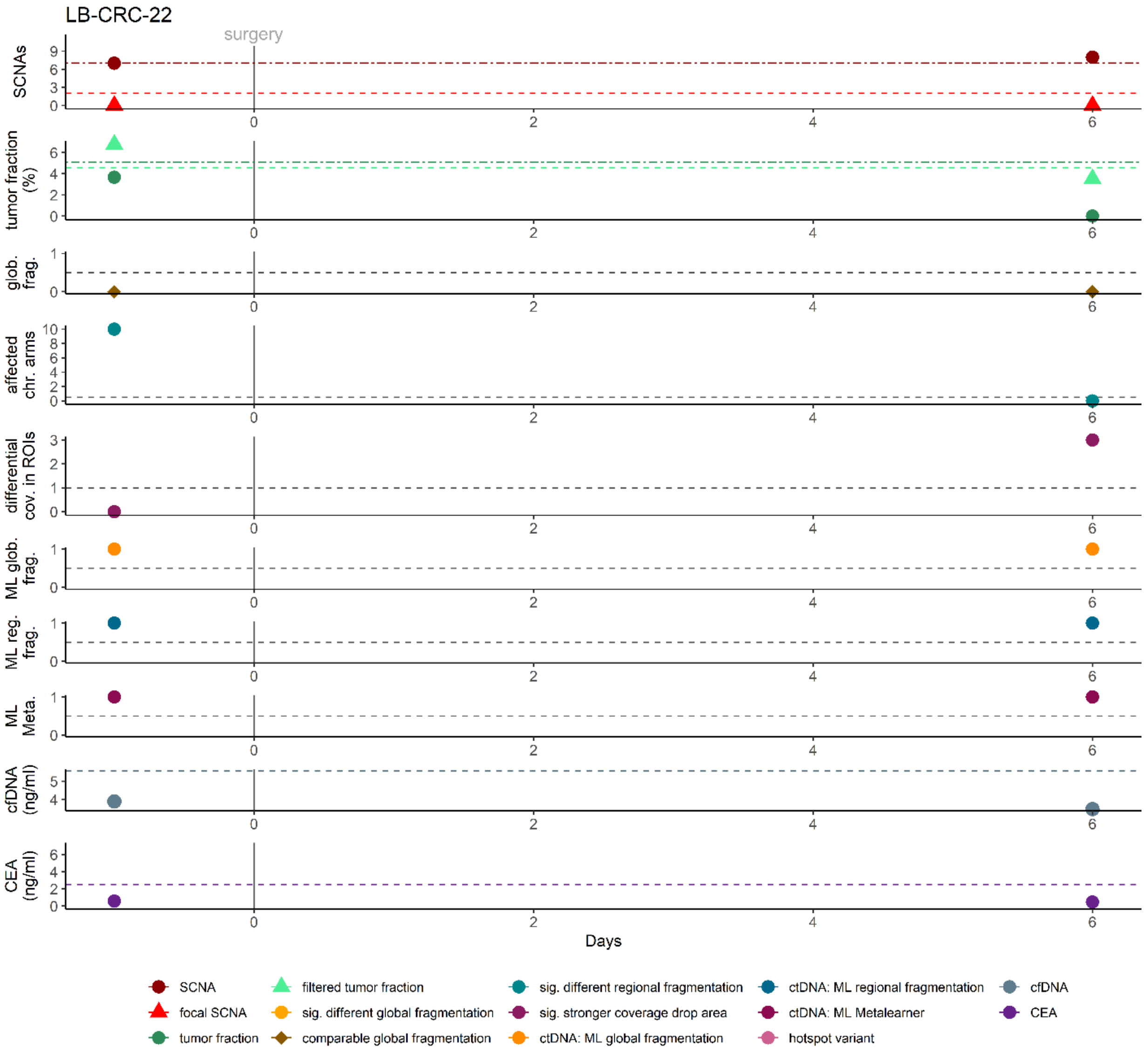

Supplementary Figure 29 Residual disease detection in patient LB-CRC-22.

Patient LB-CRC-22 was diagnosed with stage IIIC CRC.

LB-CRC-27

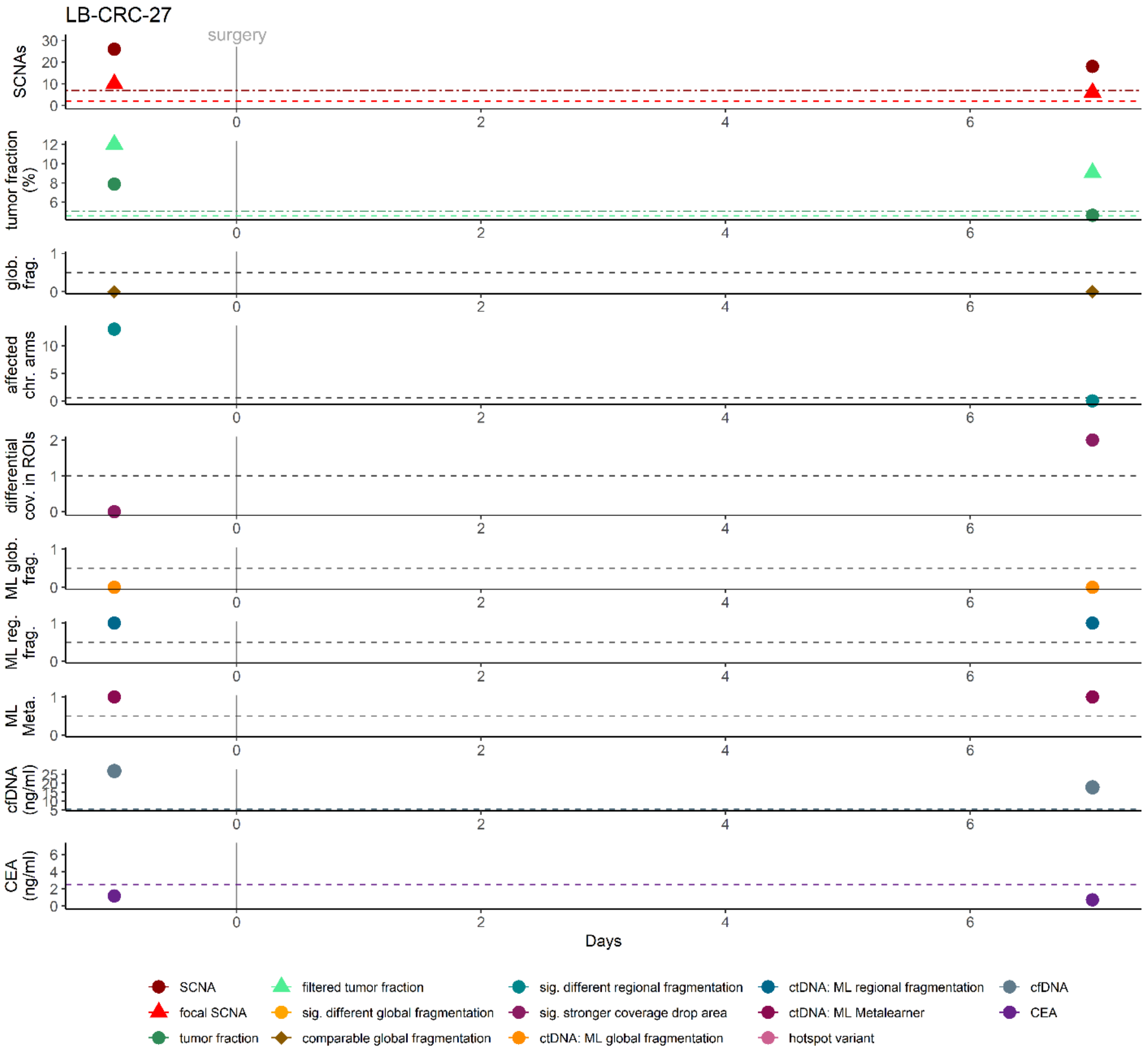

Supplementary Figure 30 Residual disease detection in patient LB-CRC-27.

Patient LB-CRC-27 was diagnosed with stage IVA CRC.

LB-CRC-28

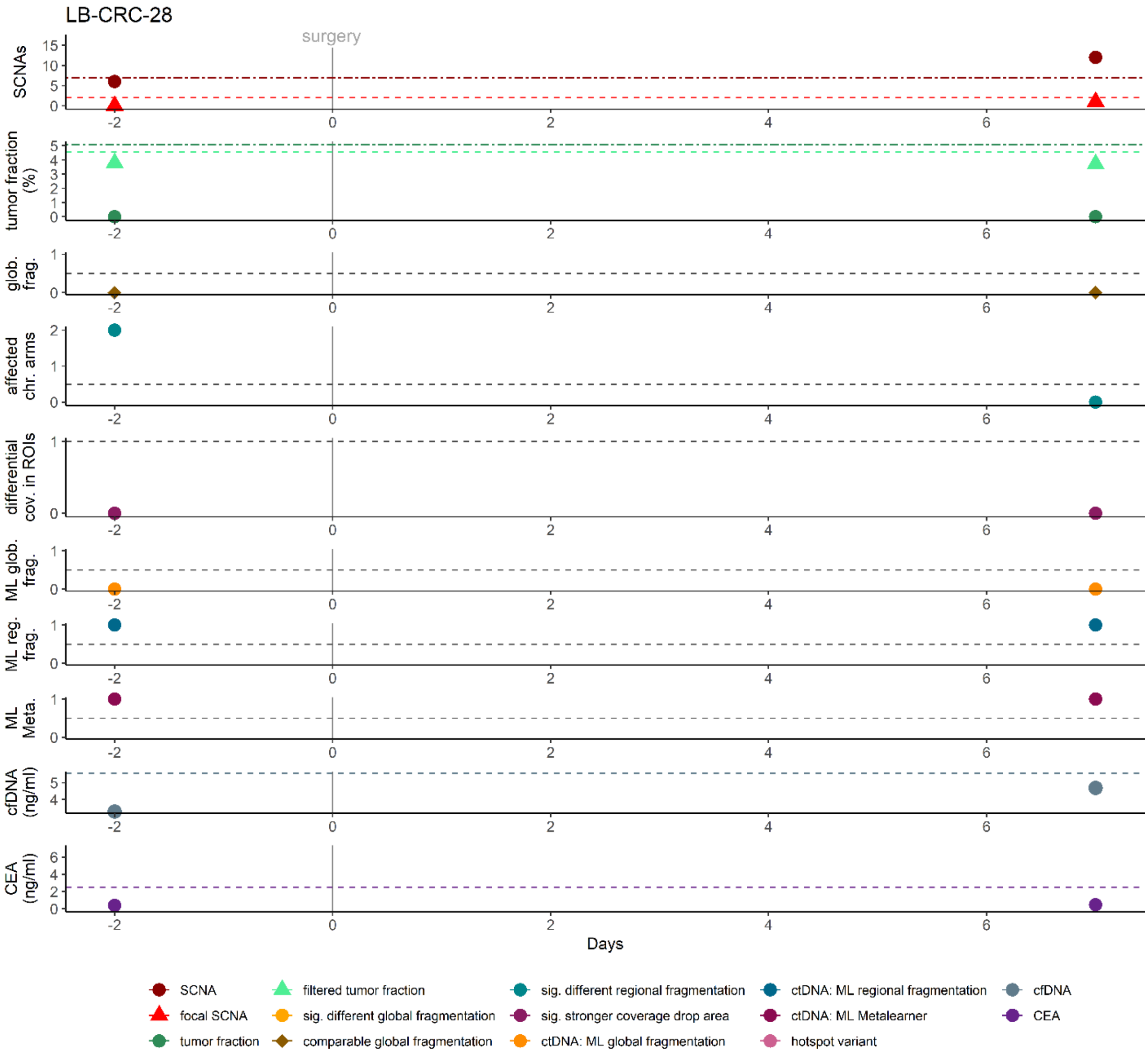

Supplementary Figure 31 Residual disease detection in patient LB-CRC-28.

Patient LB-CRC-28 was diagnosed with stage I CRC.

LB-CRC-31

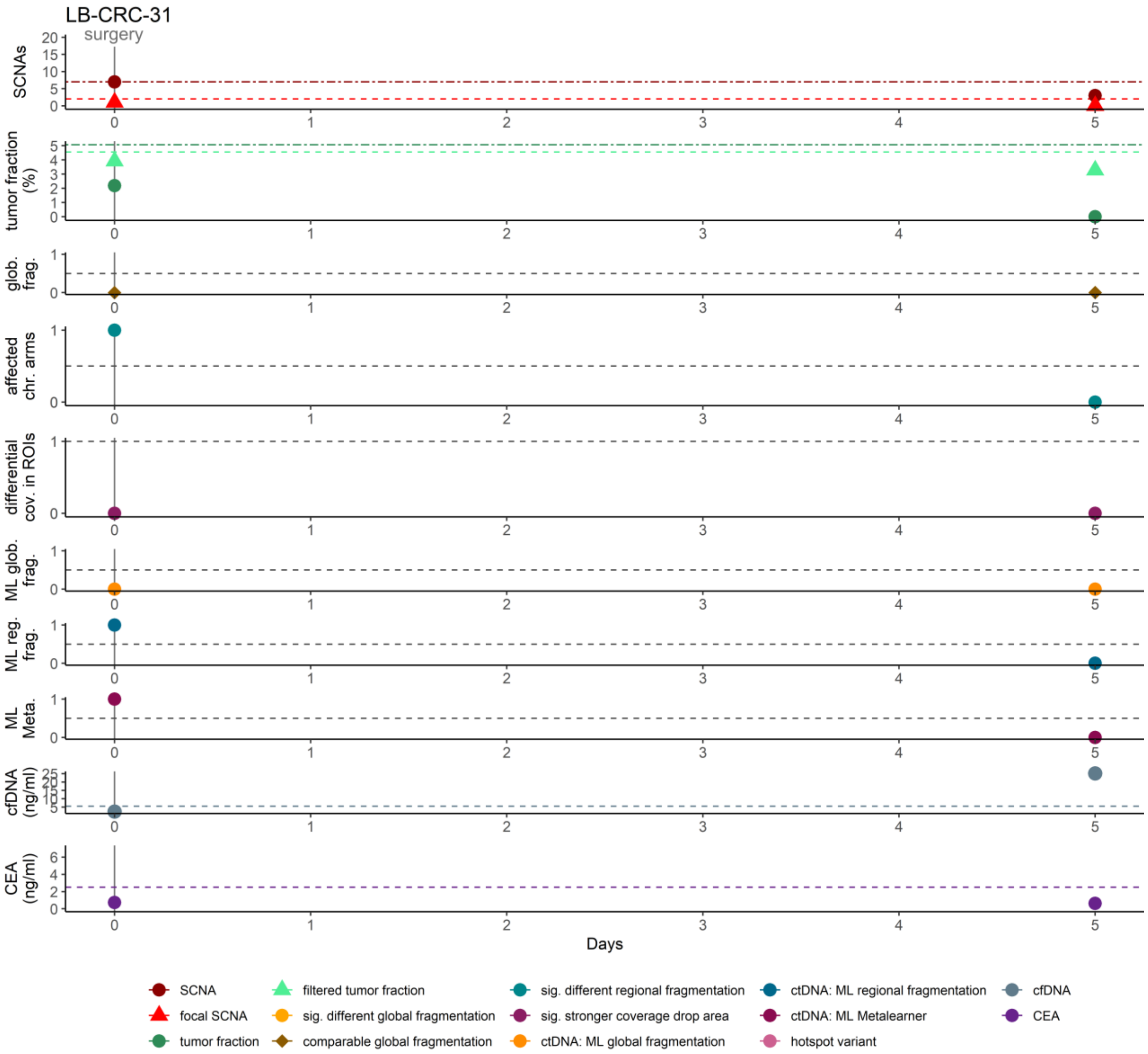

Supplementary Figure 32 Residual disease detection in patient LB-CRC-31.

Patient LB-CRC-31 was diagnosed with stage IIA CRC.

LB-CRC-36

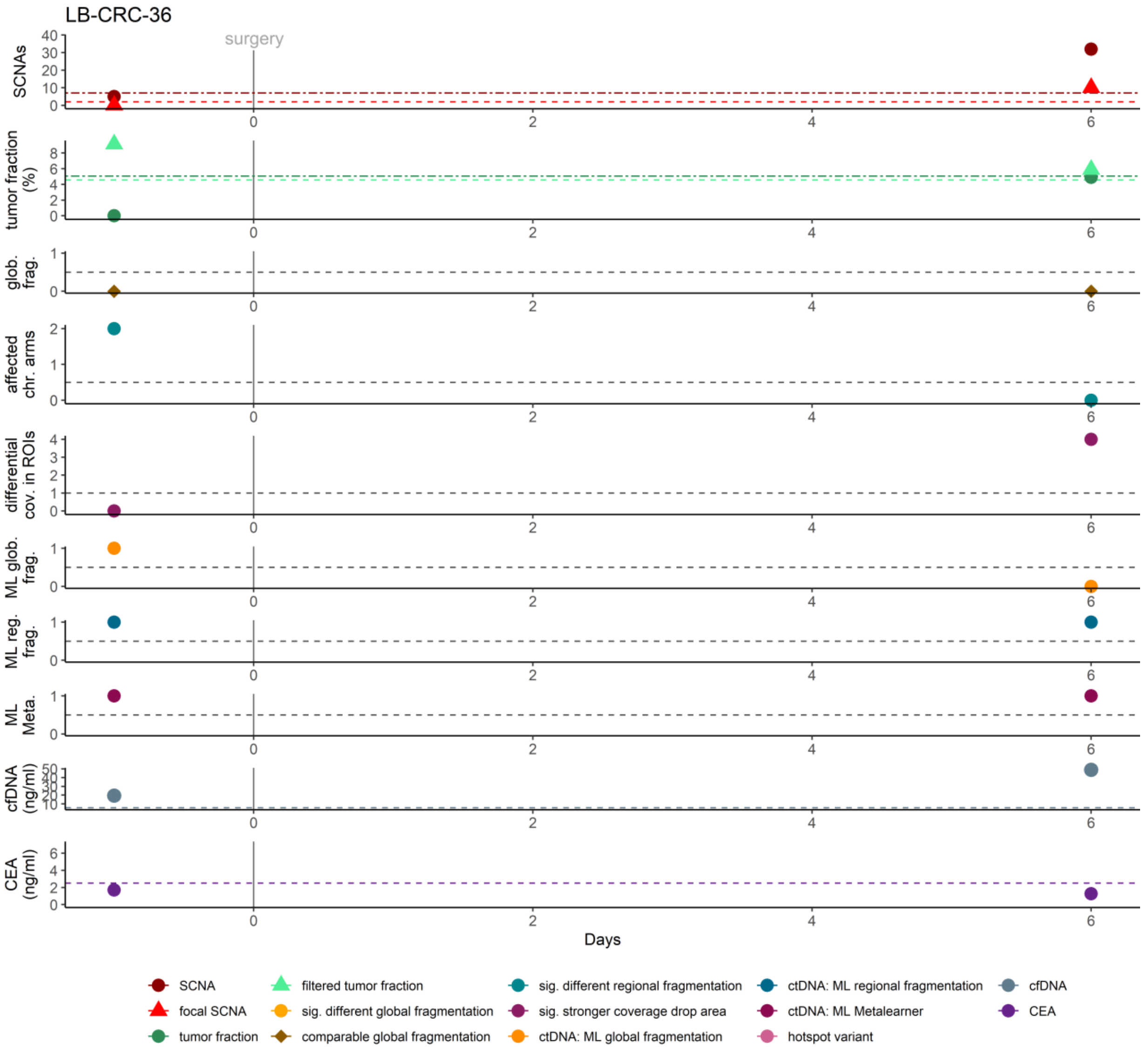

Supplementary Figure 33 Residual disease detection in patient LB-CRC-36.

Patient LB-CRC-36 was diagnosed with stage IVA CRC.

LB-CRC-38

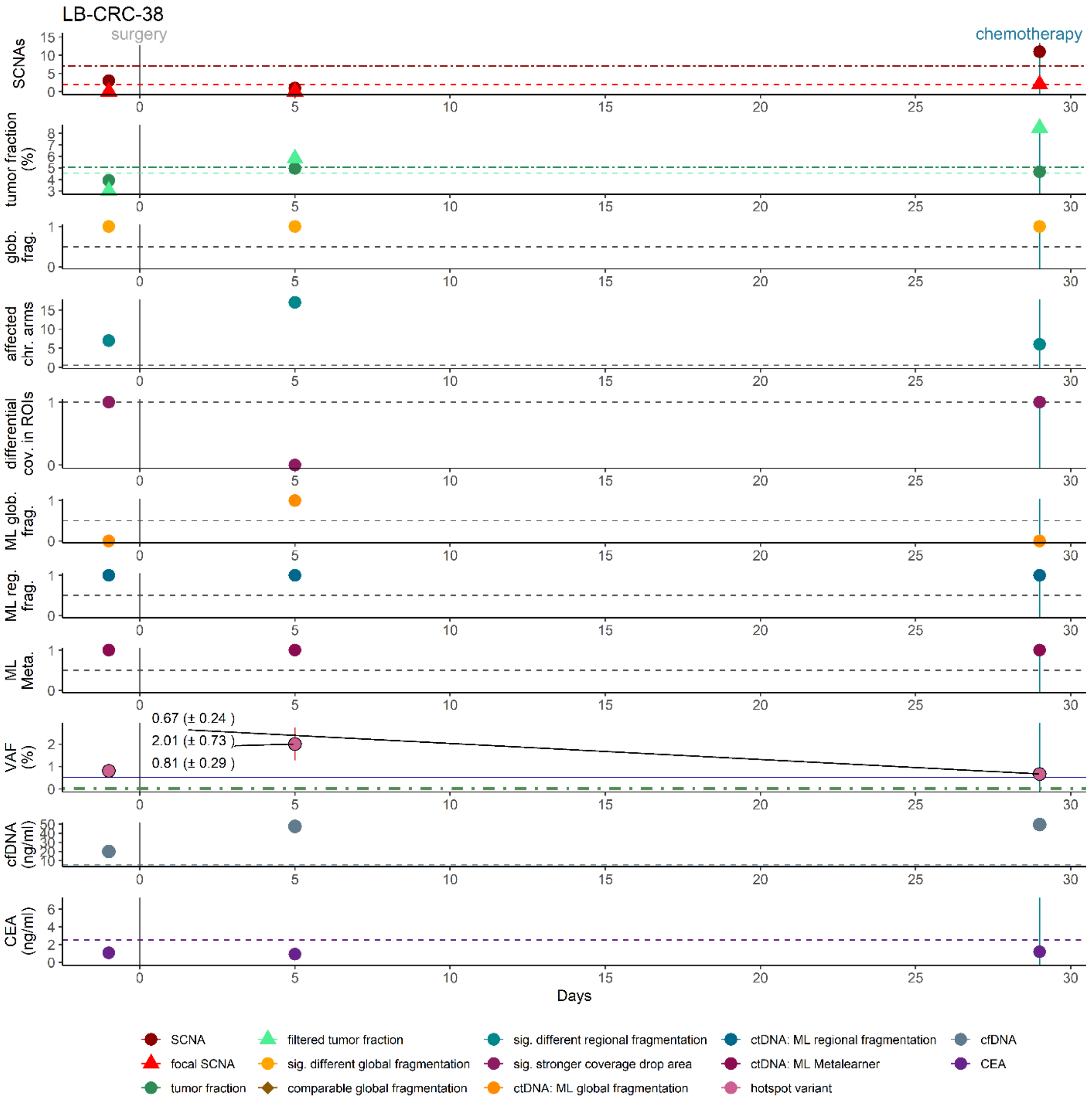

Supplementary Figure 34 Residual disease detection in patient LB-CRC-38.

Patient LB-CRC-38 was diagnosed with stage IVC CRC.

LB-CRC-42

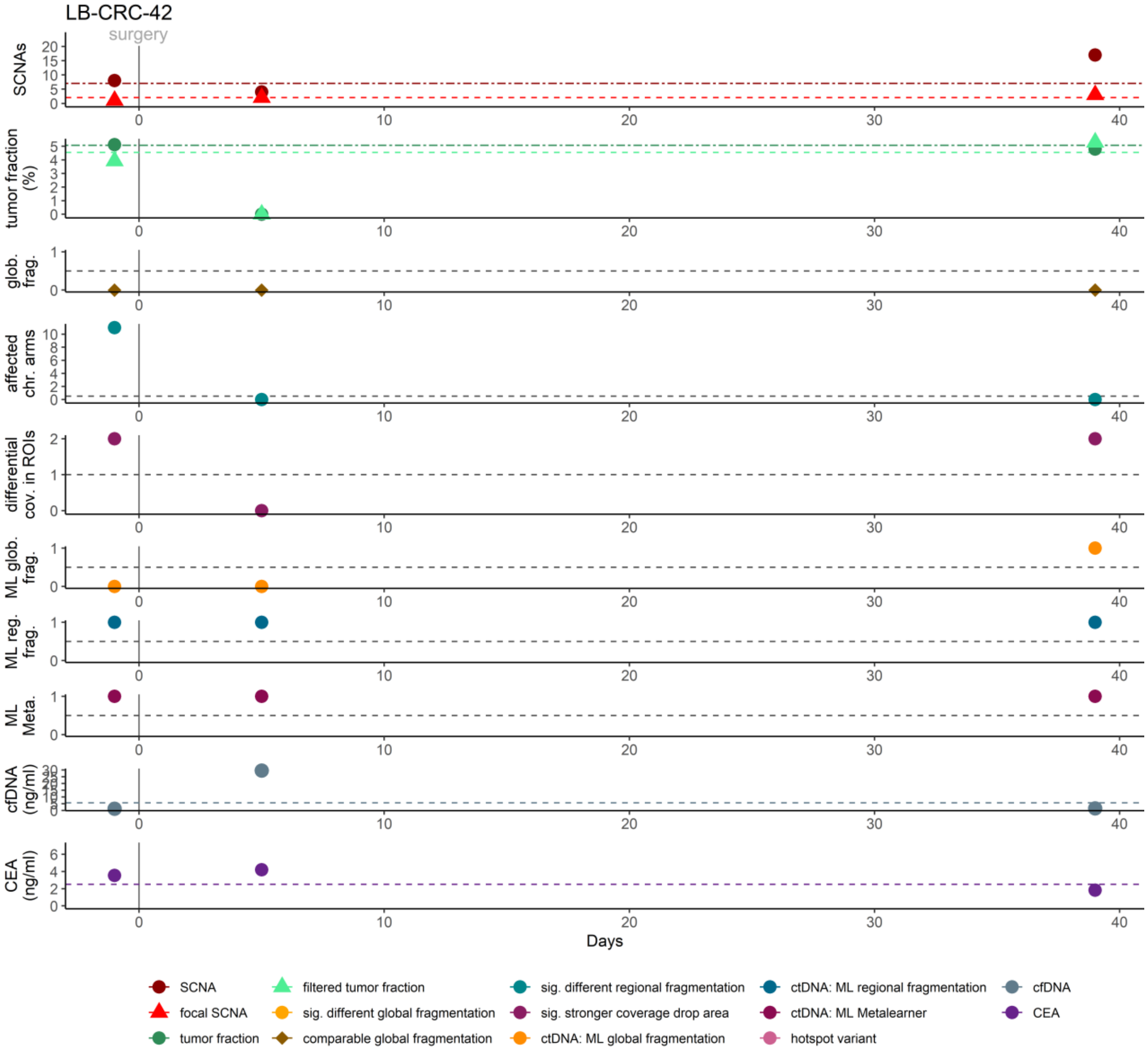

Supplementary Figure 35 Residual disease detection in patient LB-CRC-42.

Patient LB-CRC-42 was diagnosed with stage I CRC.

LB-CRC-44

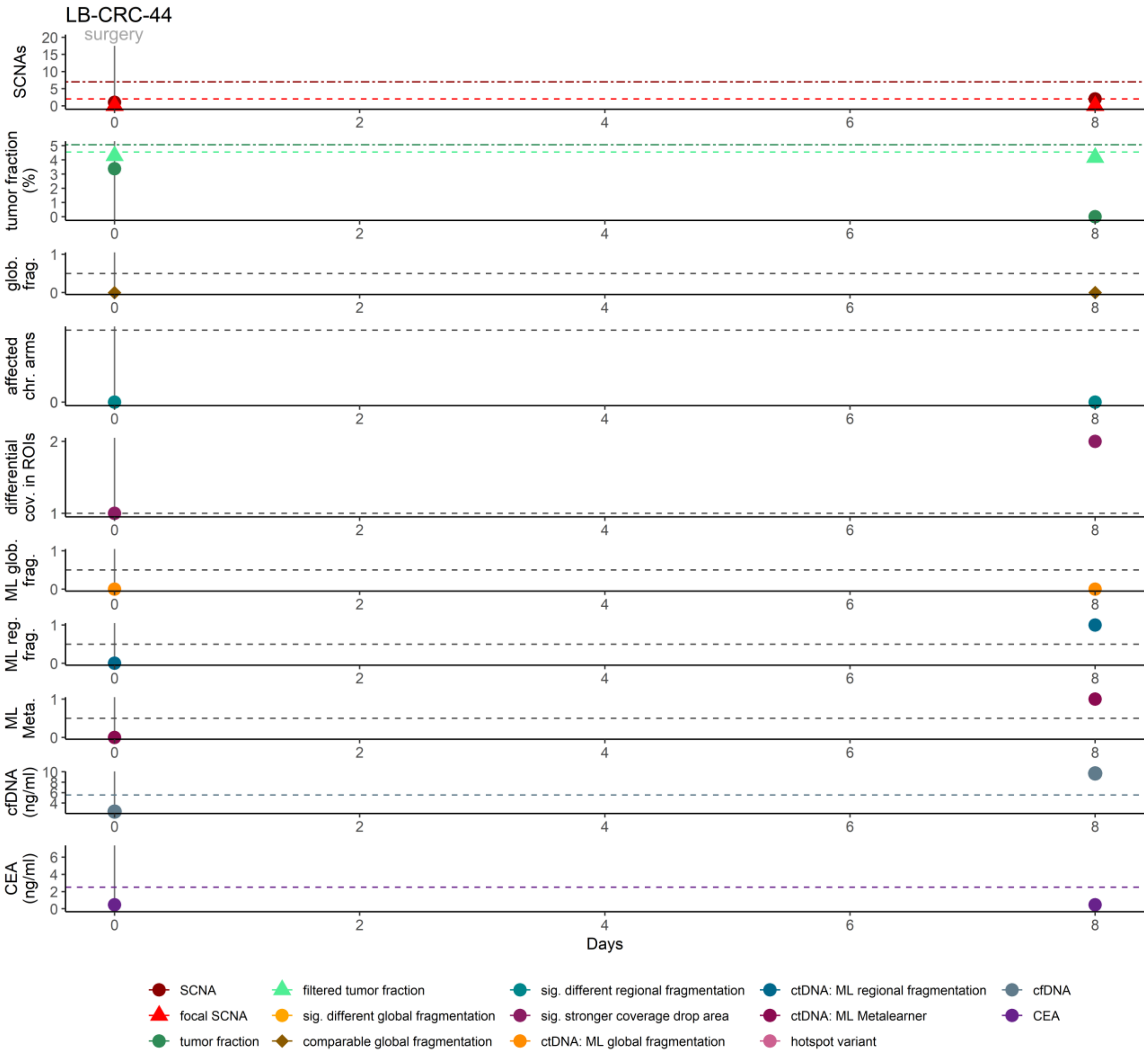

Supplementary Figure 36 Residual disease detection in patient LB-CRC-44.

Patient LB-CRC-44 was diagnosed with stage IIIB CRC.

LB-CRC-45

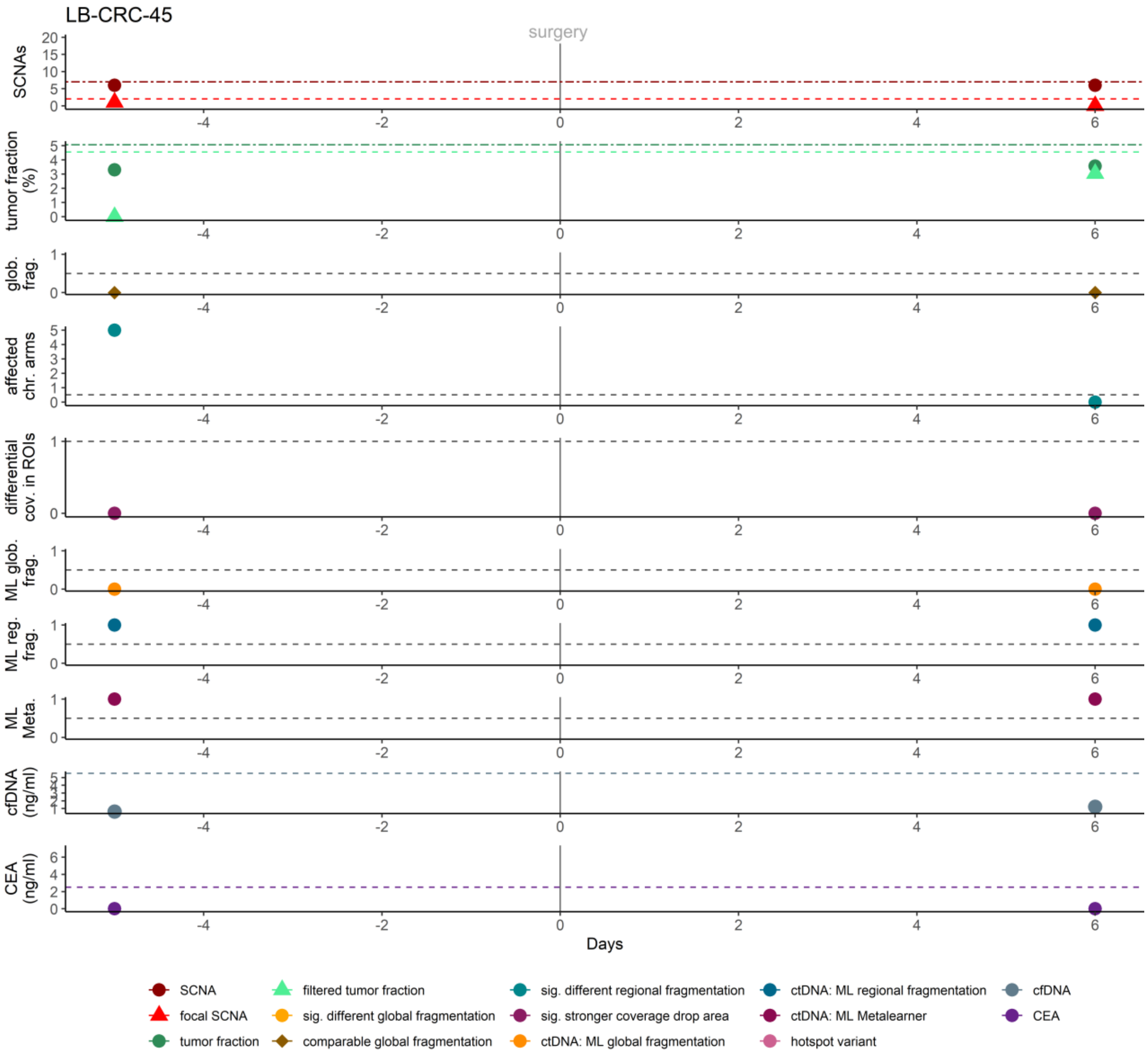

Supplementary Figure 37 Residual disease detection in patient LB-CRC-45.

Patient LB-CRC-45 was diagnosed with stage I CRC.

LB-CRC-46

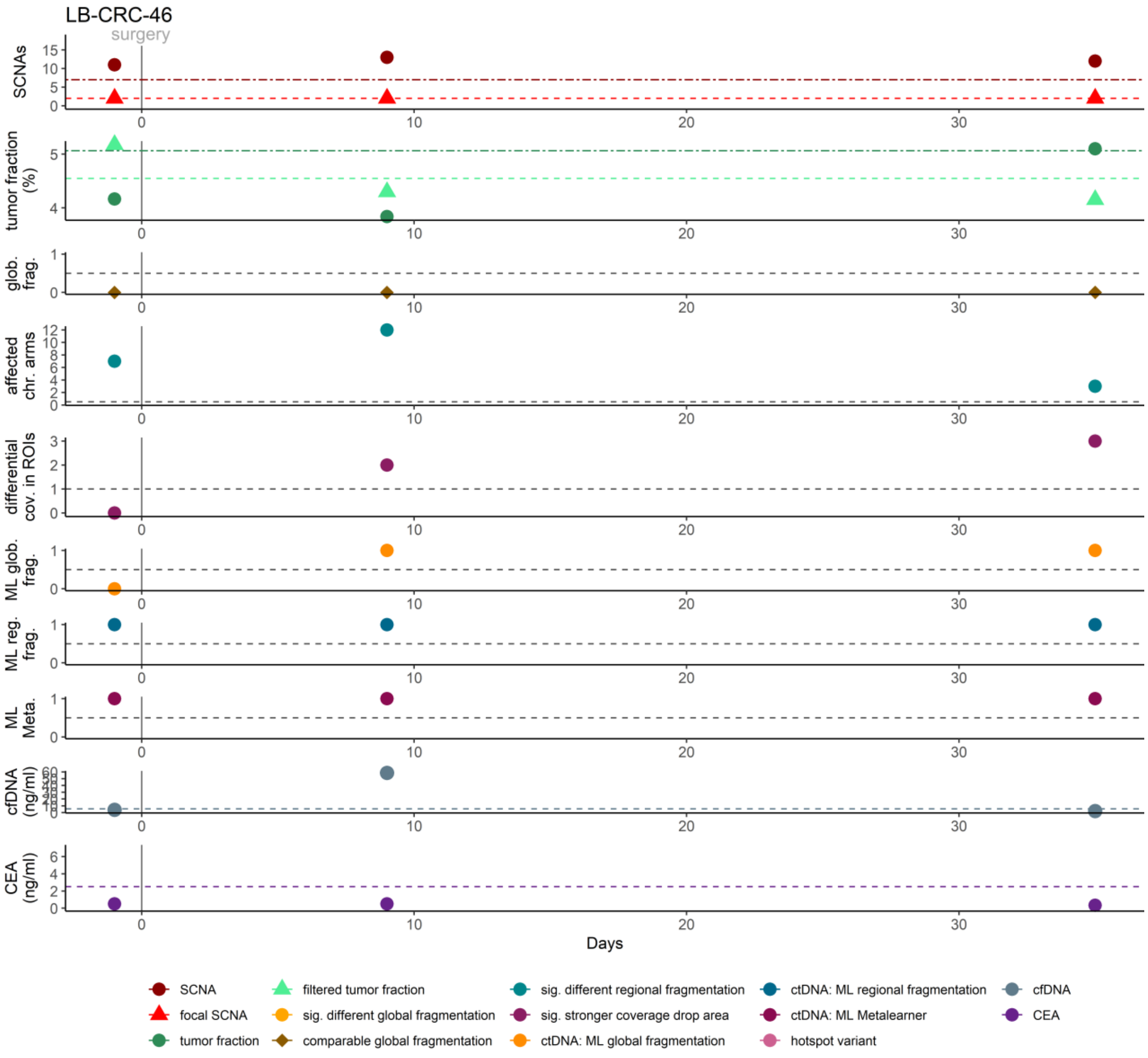

Supplementary Figure 38 Residual disease detection in patient LB-CRC-46.

Patient LB-CRC-46 was diagnosed with stage IVA CRC.

LB-CRC-47

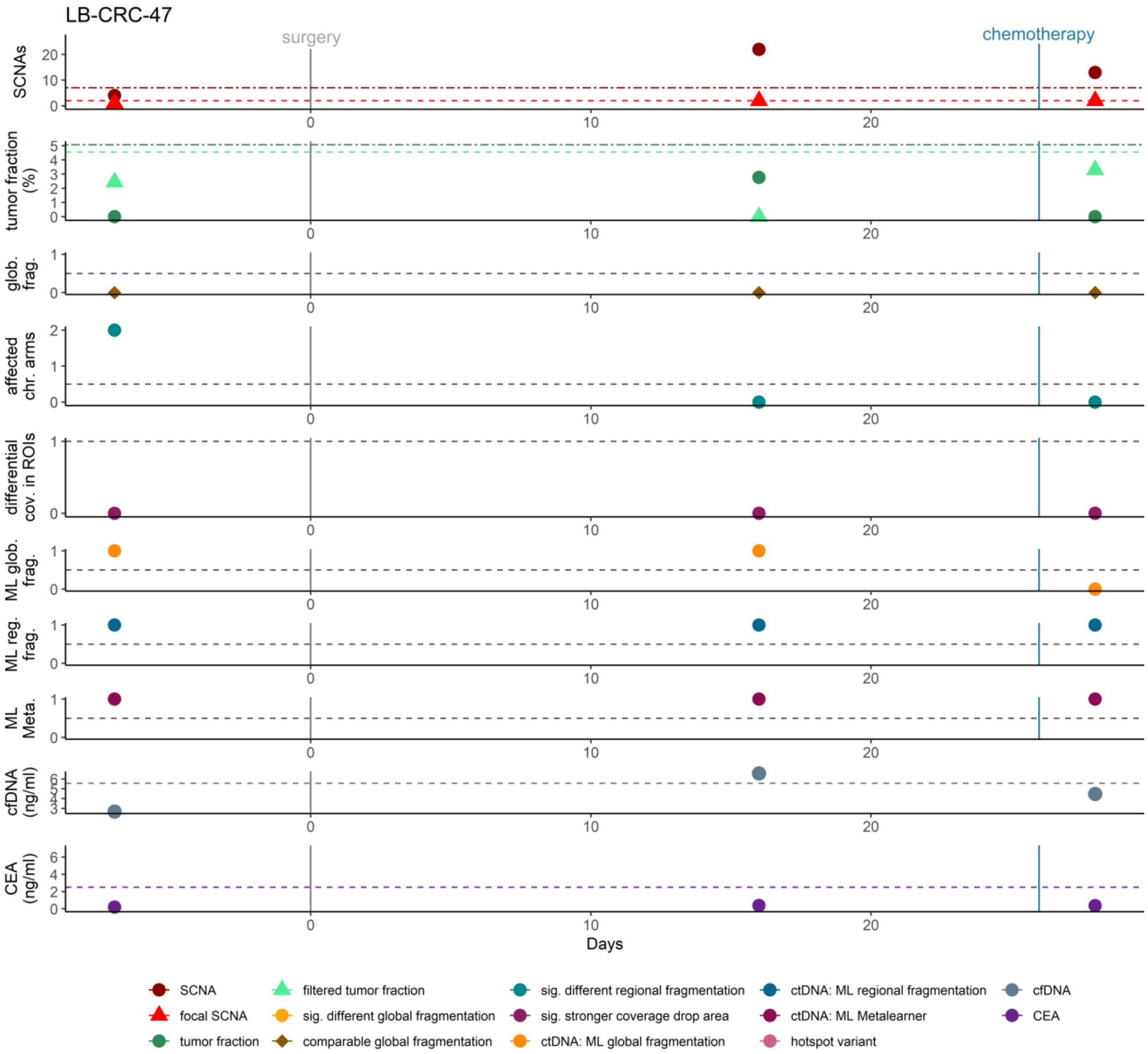

Supplementary Figure 39 Residual disease detection in patient LB-CRC-47.

Patient LB-CRC-47 was diagnosed with stage III CRC.

LB-CRC-49

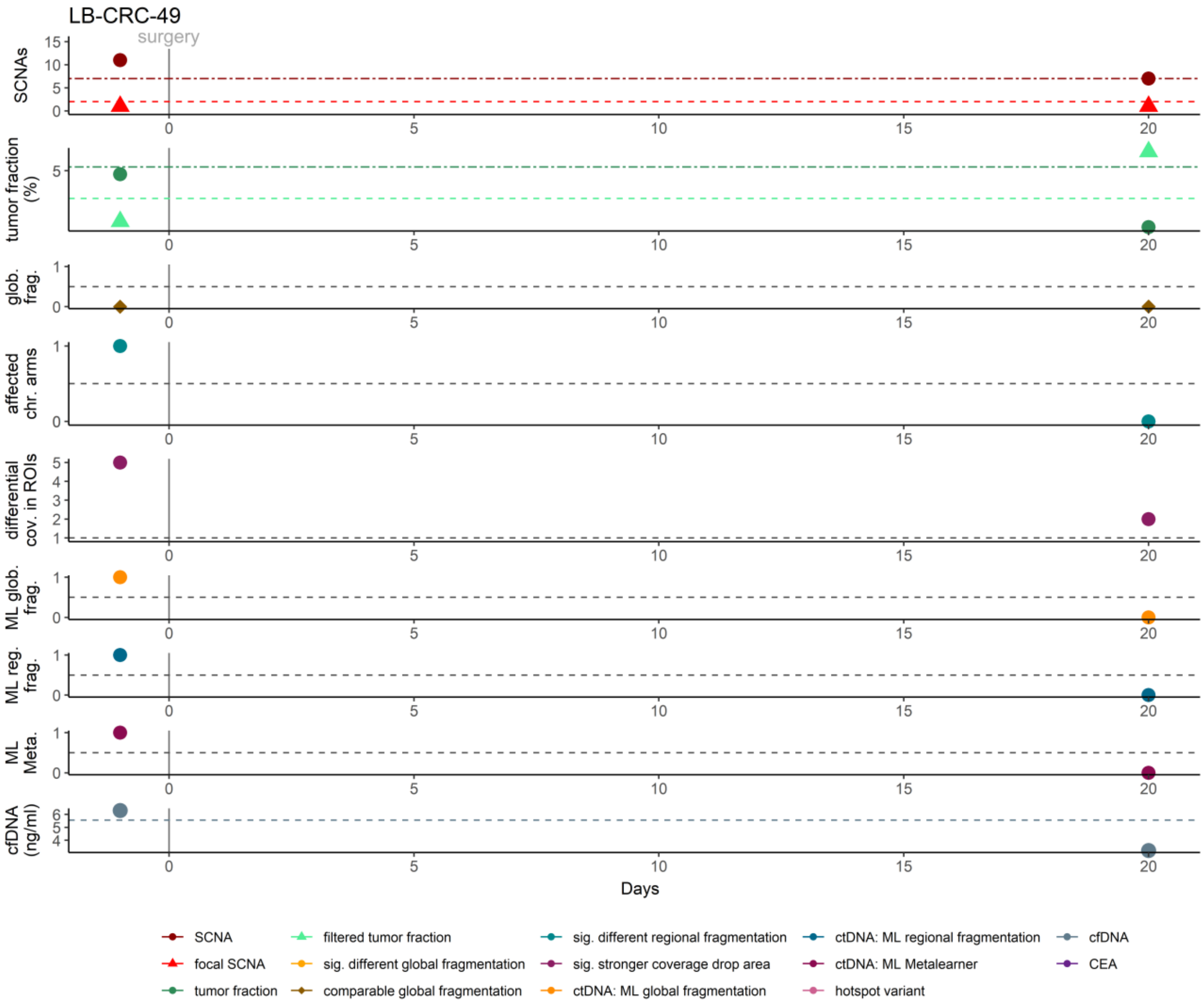

Supplementary Figure 40 Residual disease detection in patient LB-CRC-49.

Patient LB-CRC-49 was diagnosed with stage IIA CRC.

LB-CRC-50

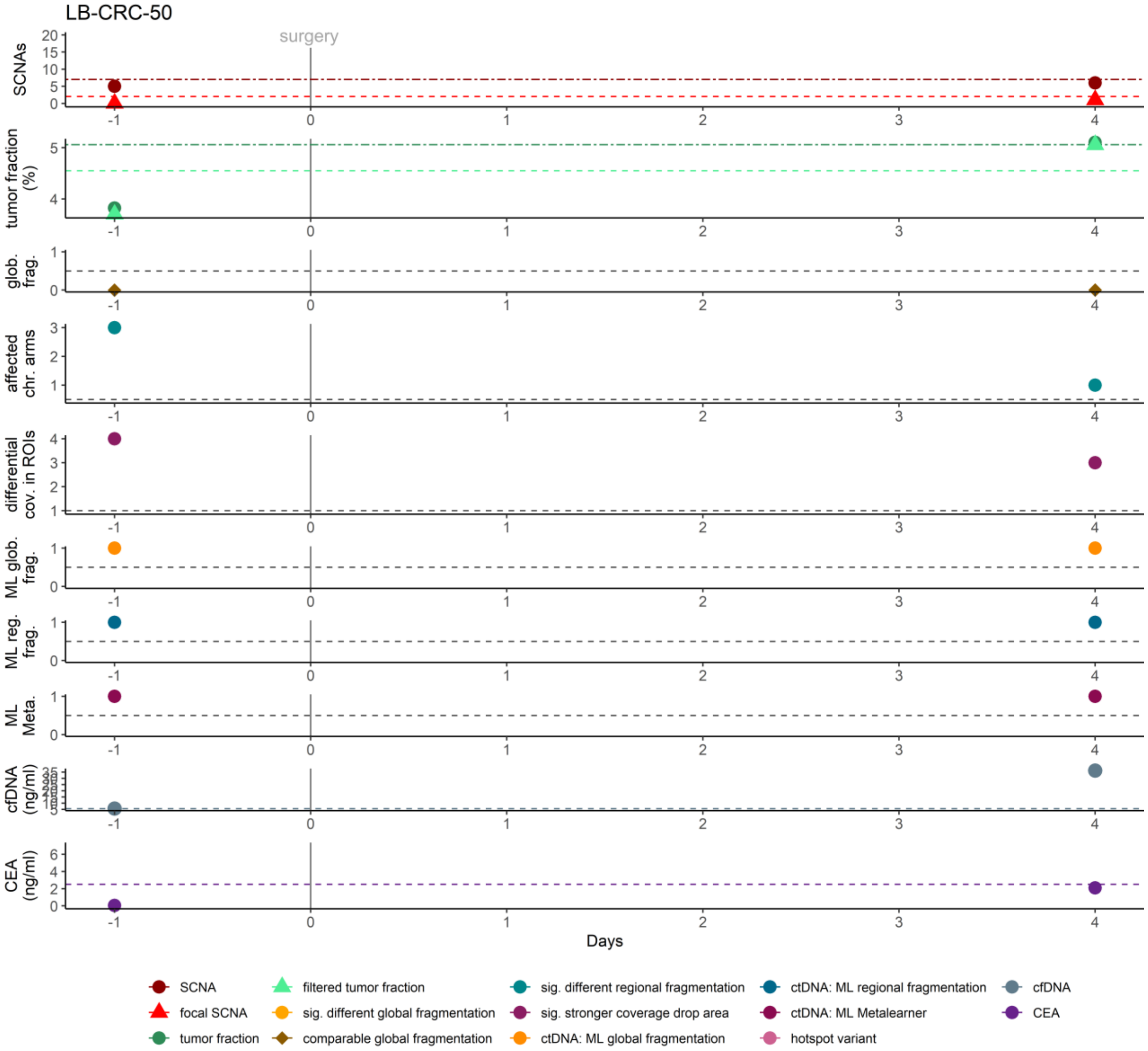

Supplementary Figure 41 Residual disease detection in patient LB-CRC-50.

Patient LB-CRC-50 was diagnosed with stage I CRC.

LB-CRC-51

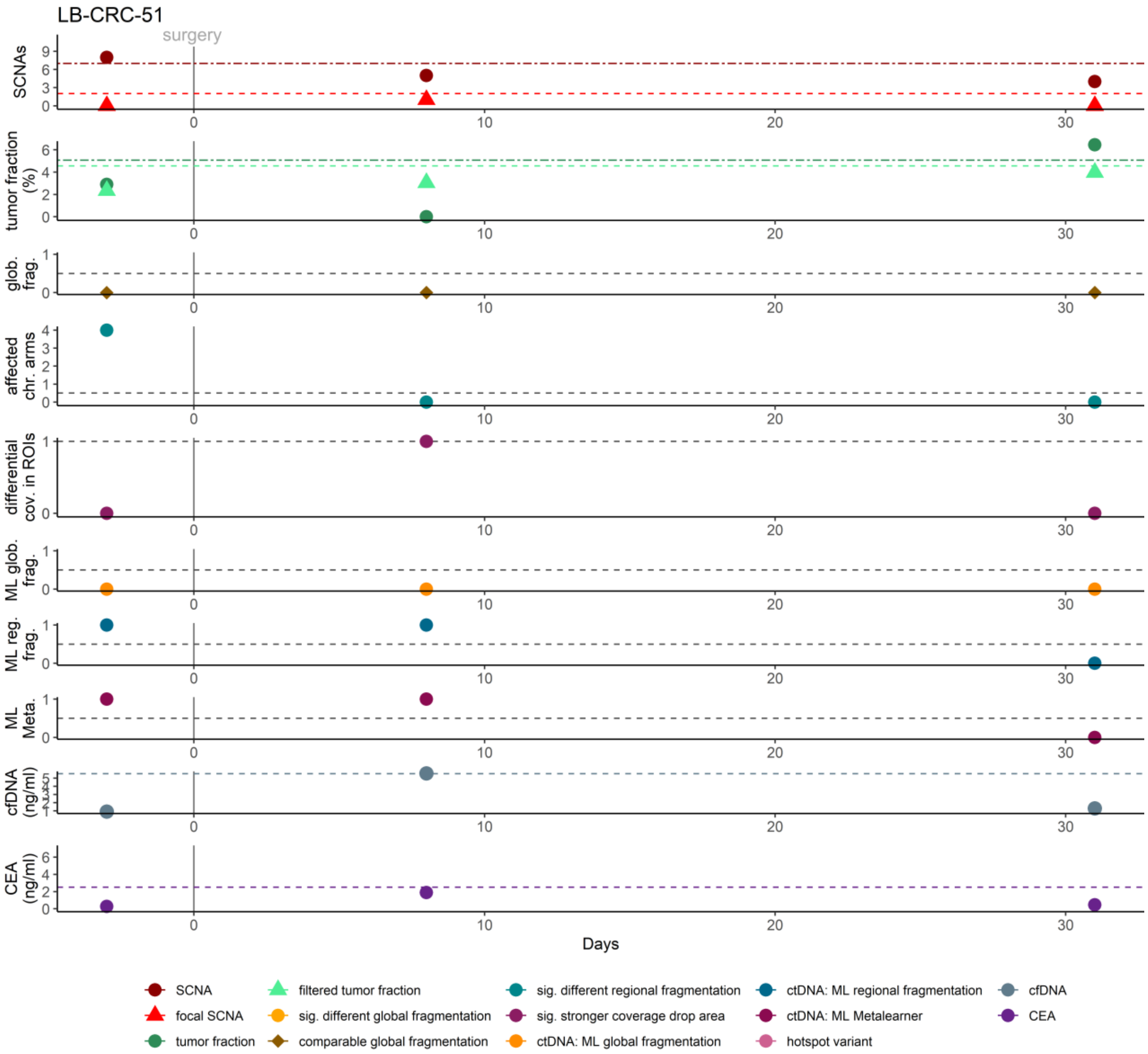

Supplementary Figure 42 Residual disease detection in patient LB-CRC-51.

Patient LB-CRC-51 was diagnosed with stage IIA CRC.

LB-CRC-52

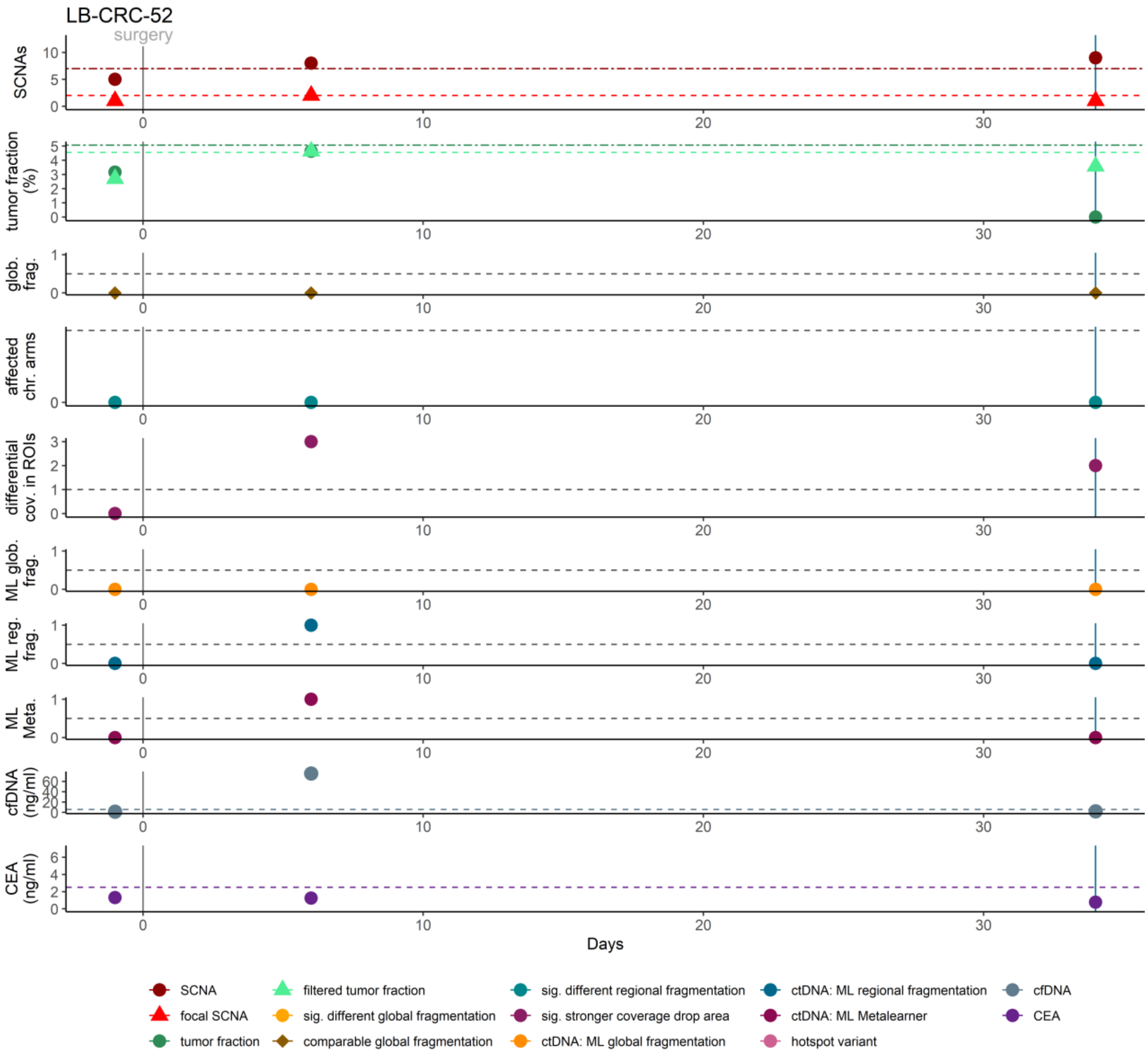

Supplementary Figure 43 Residual disease detection in patient LB-CRC-52.

Patient LB-CRC-52 was diagnosed with stage IIIC CRC.

LB-CRC-53

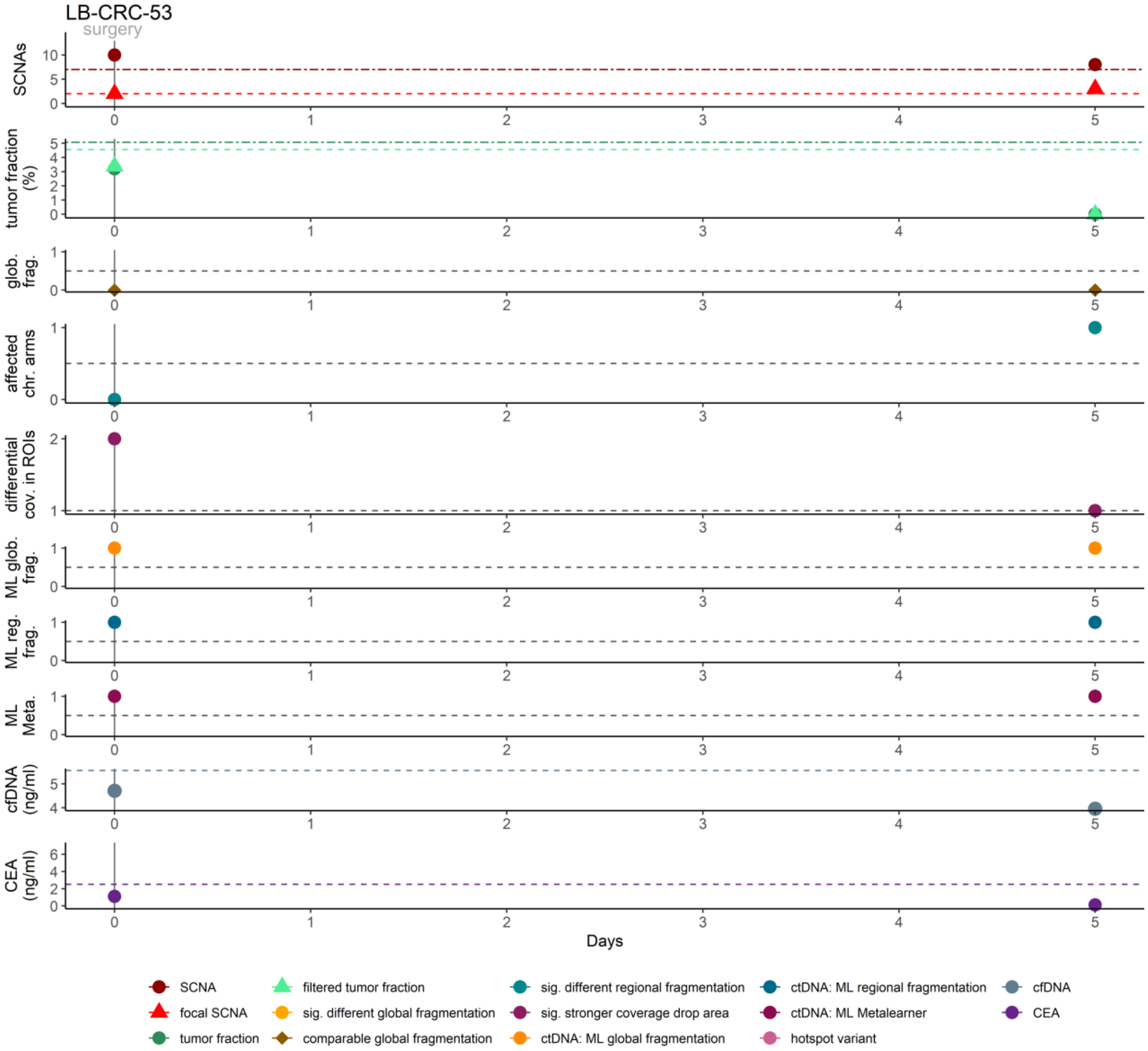

Supplementary Figure 44 Residual disease detection in patient LB-CRC-53.

Patient LB-CRC-53 was diagnosed with stage IIIC CRC.

LB-CRC-56

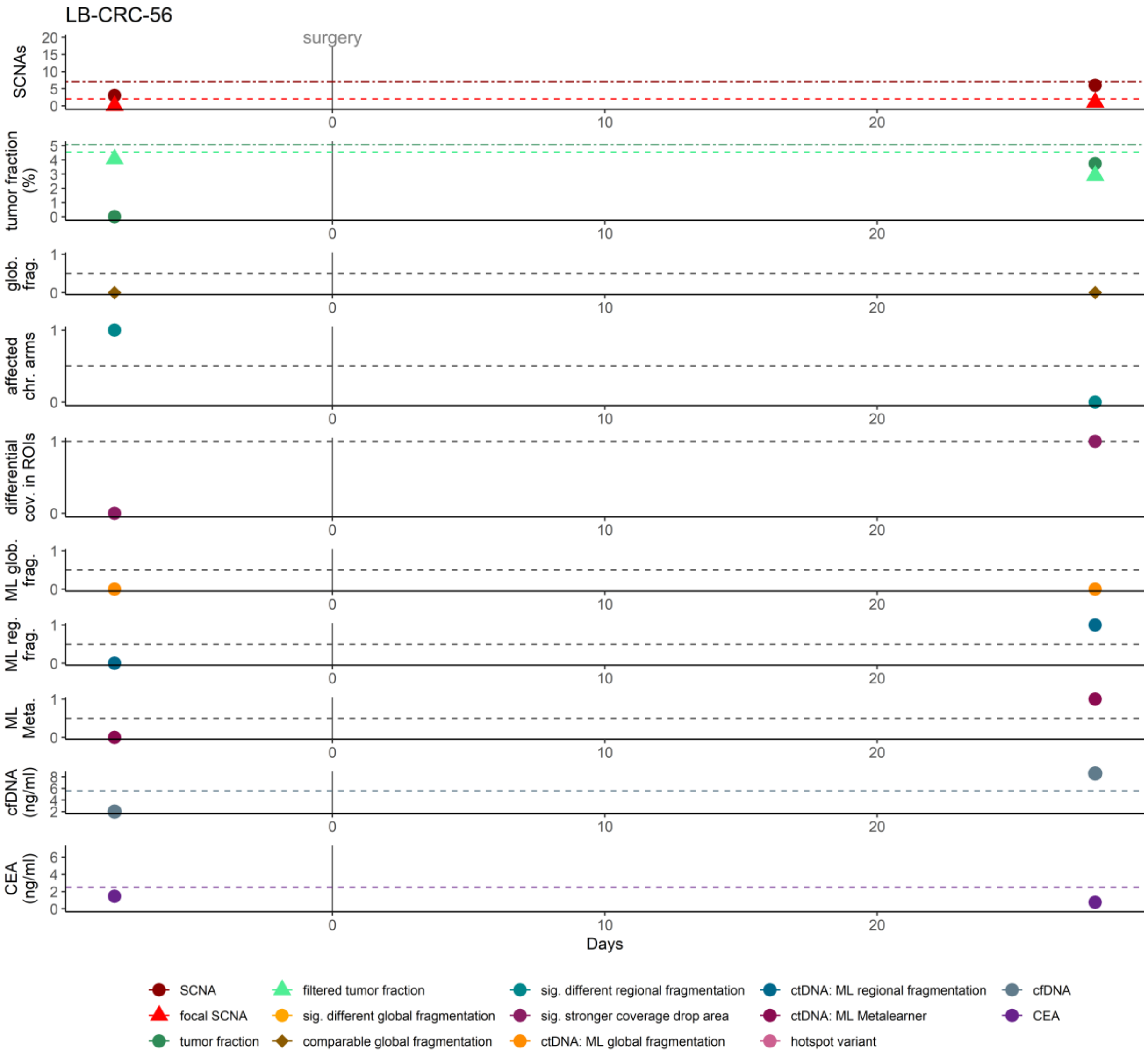

Supplementary Figure 45 Residual disease detection in patient LB-CRC-56.

Patient LB-CRC-56 was diagnosed with stage I CRC.

LB-CRC-57

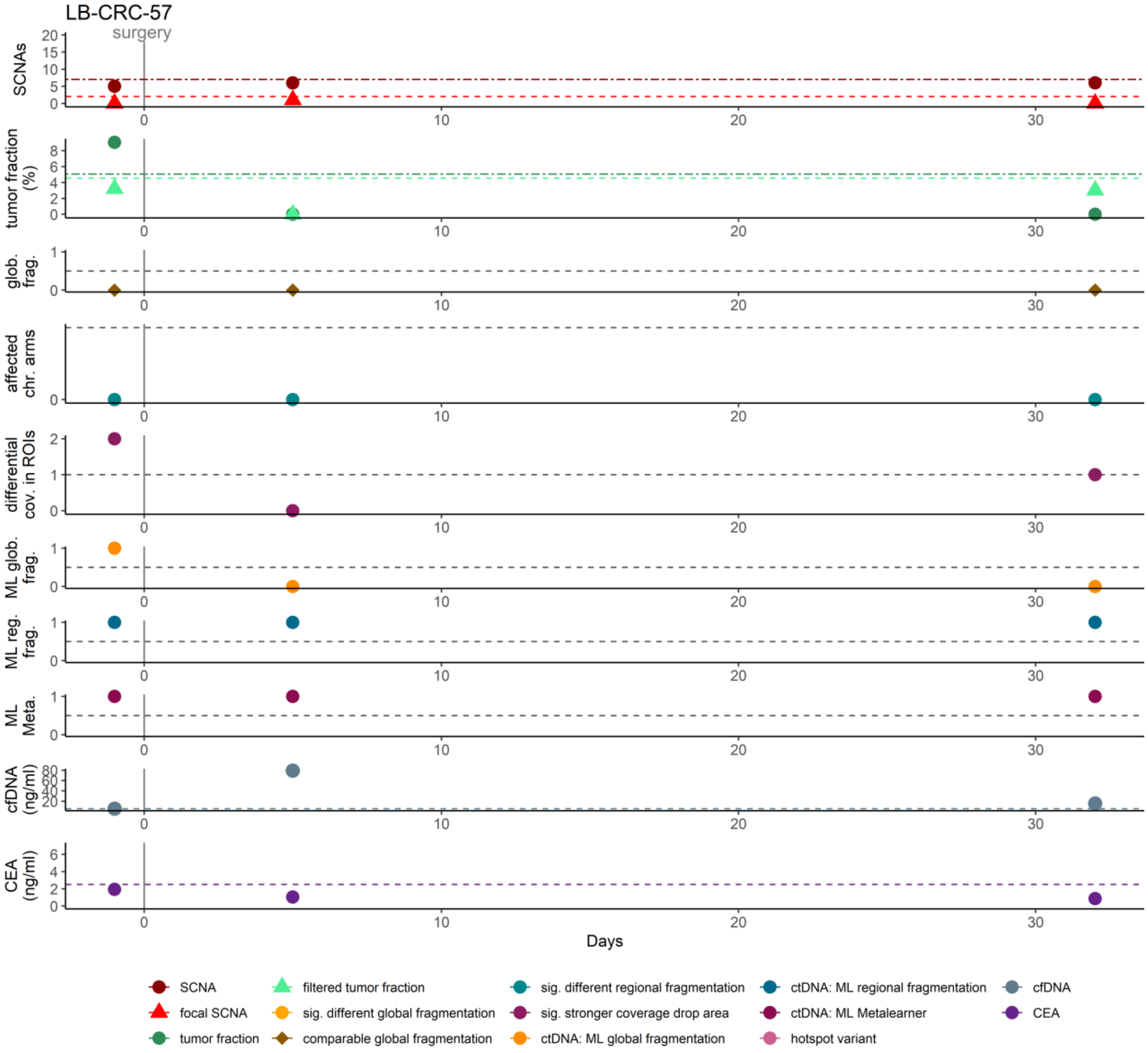

Supplementary Figure 46 Residual disease detection in patient LB-CRC-57.

Patient LB-CRC-57 was diagnosed with stage IIA CRC.

LB-CRC-58

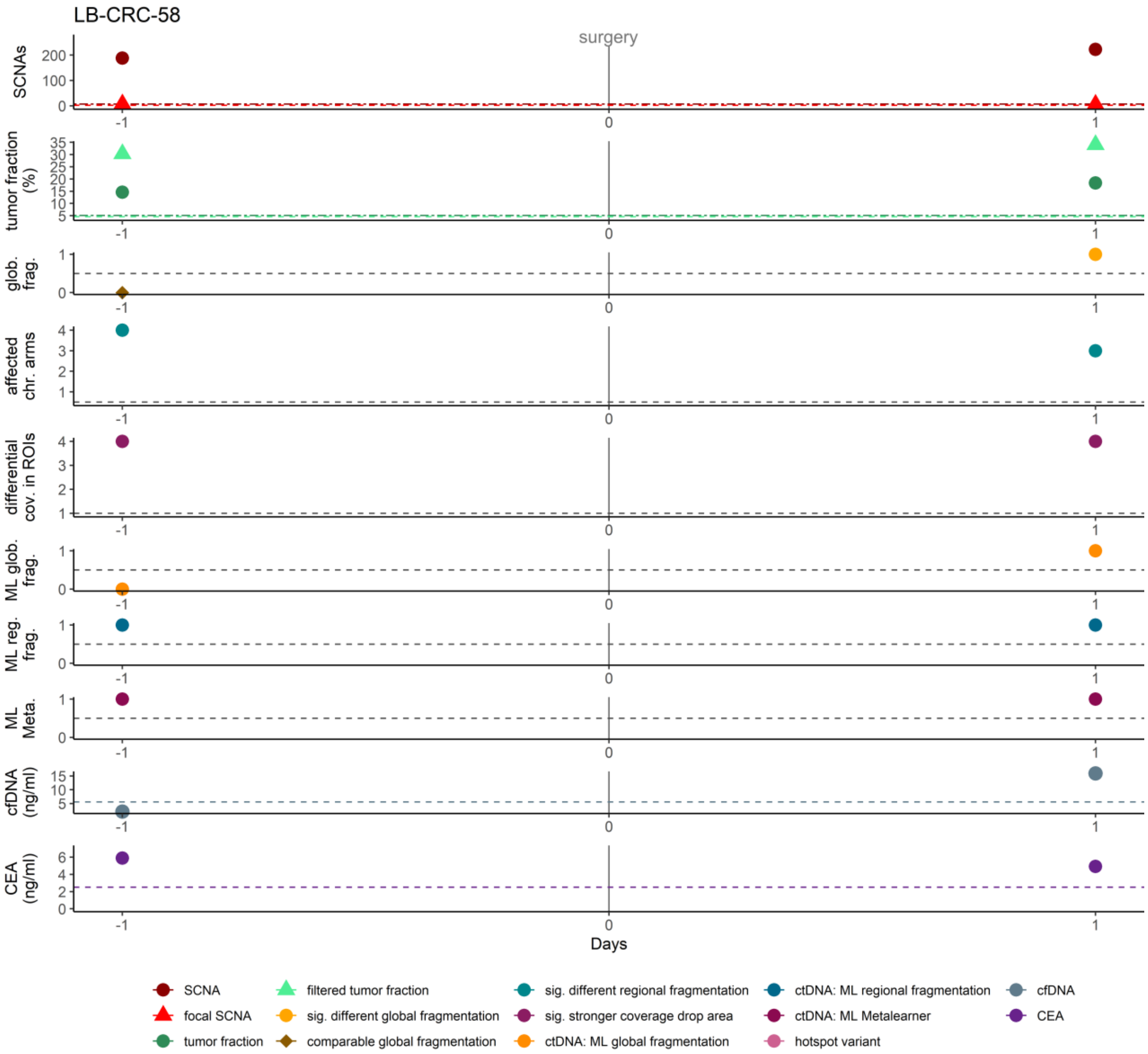

Supplementary Figure 47 Residual disease detection in patient LB-CRC-58.

Patient LB-CRC-58 was diagnosed with stage IVB CRC.

LB-CRC-60

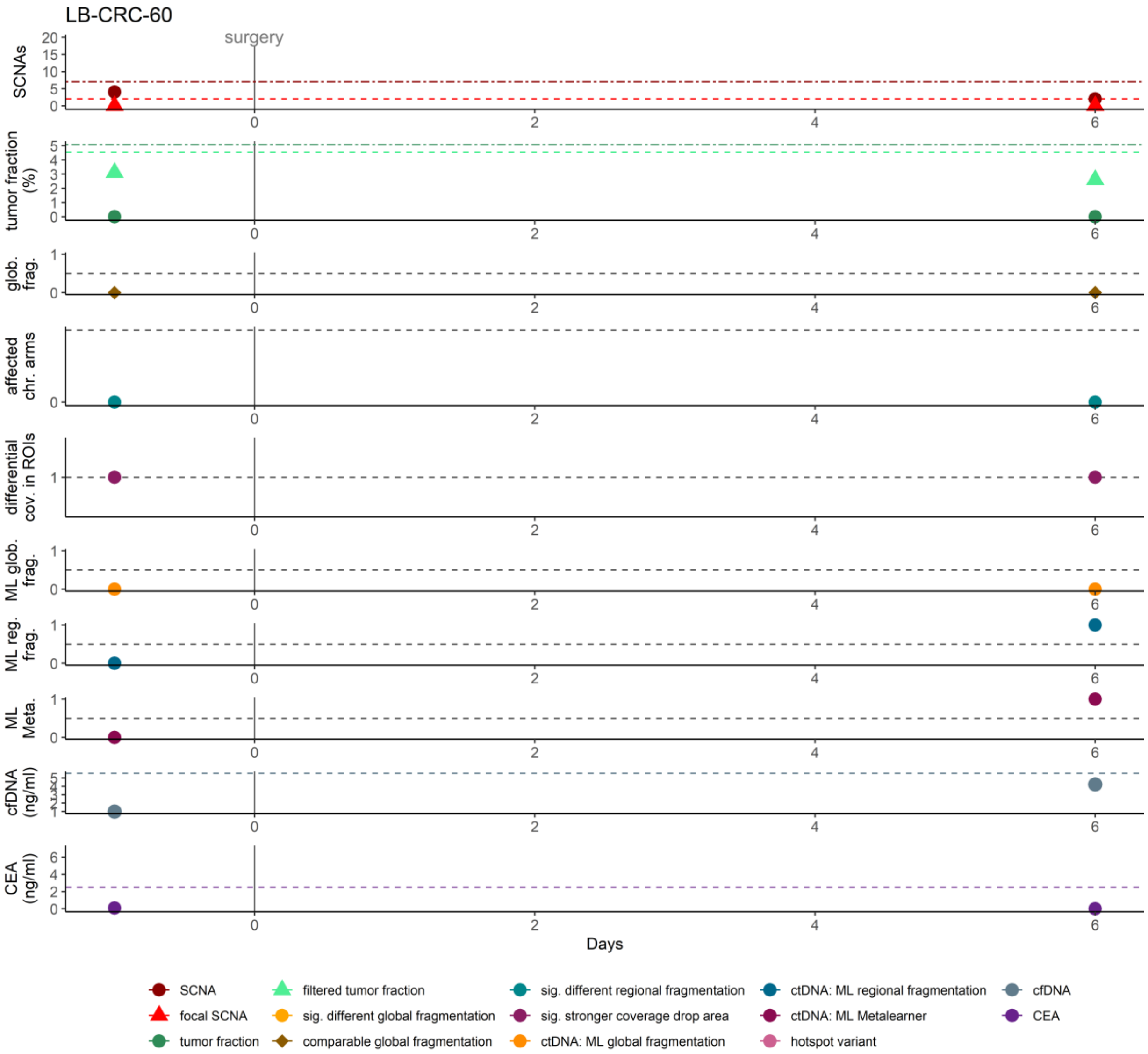

Supplementary Figure 48 Residual disease detection in patient LB-CRC-60.

Patient LB-CRC-60 was diagnosed with stage IVA CRC.

LB-CRC-62

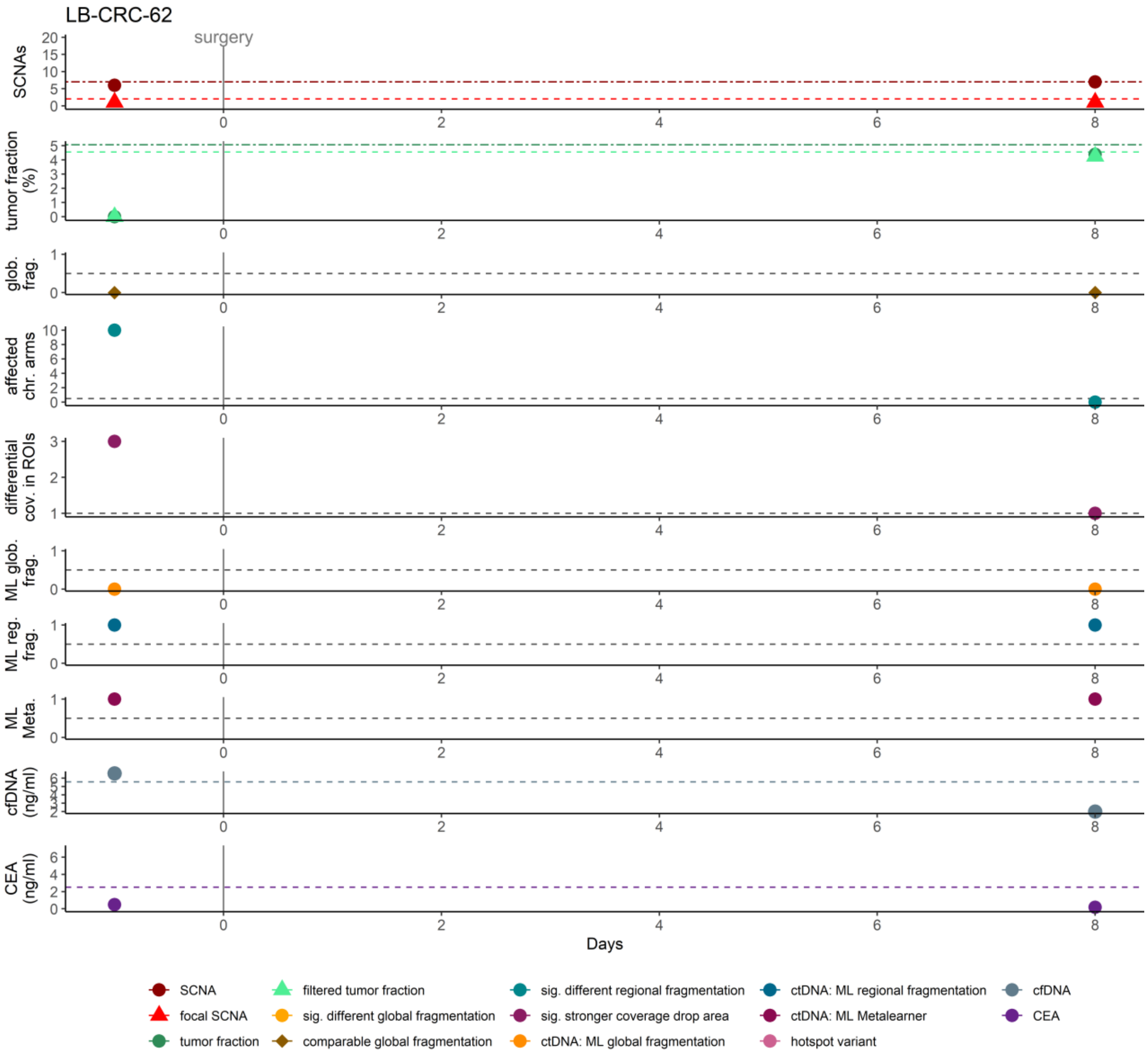

Supplementary Figure 49 Residual disease detection in patient LB-CRC-62.

Patient LB-CRC-62 was diagnosed with stage IIIB CRC.

LB-CRC-63

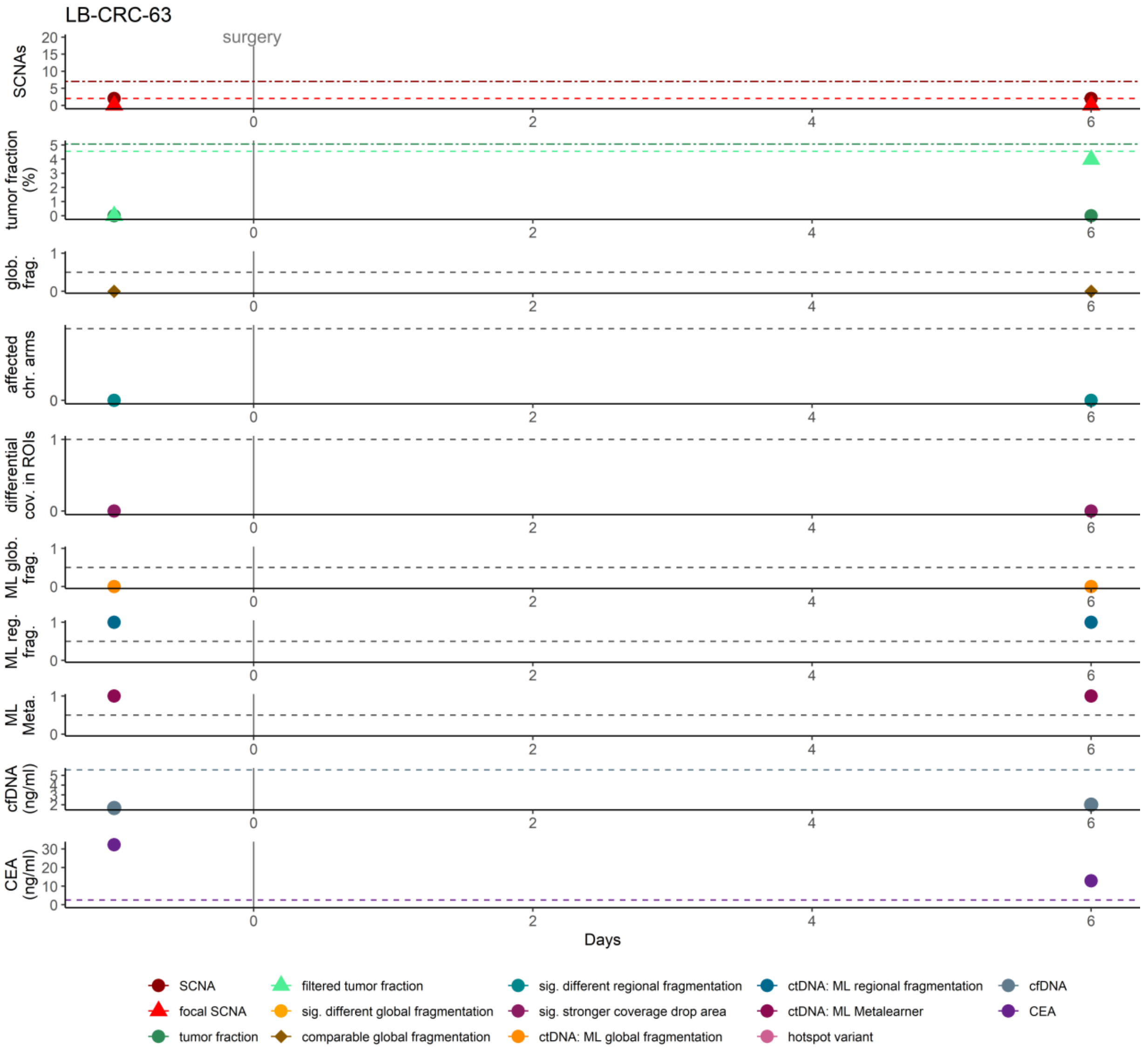

Supplementary Figure 50 Residual disease detection in patient LB-CRC-63.

Patient LB-CRC-63 was diagnosed with stage IIA CRC.

LB-CRC-64

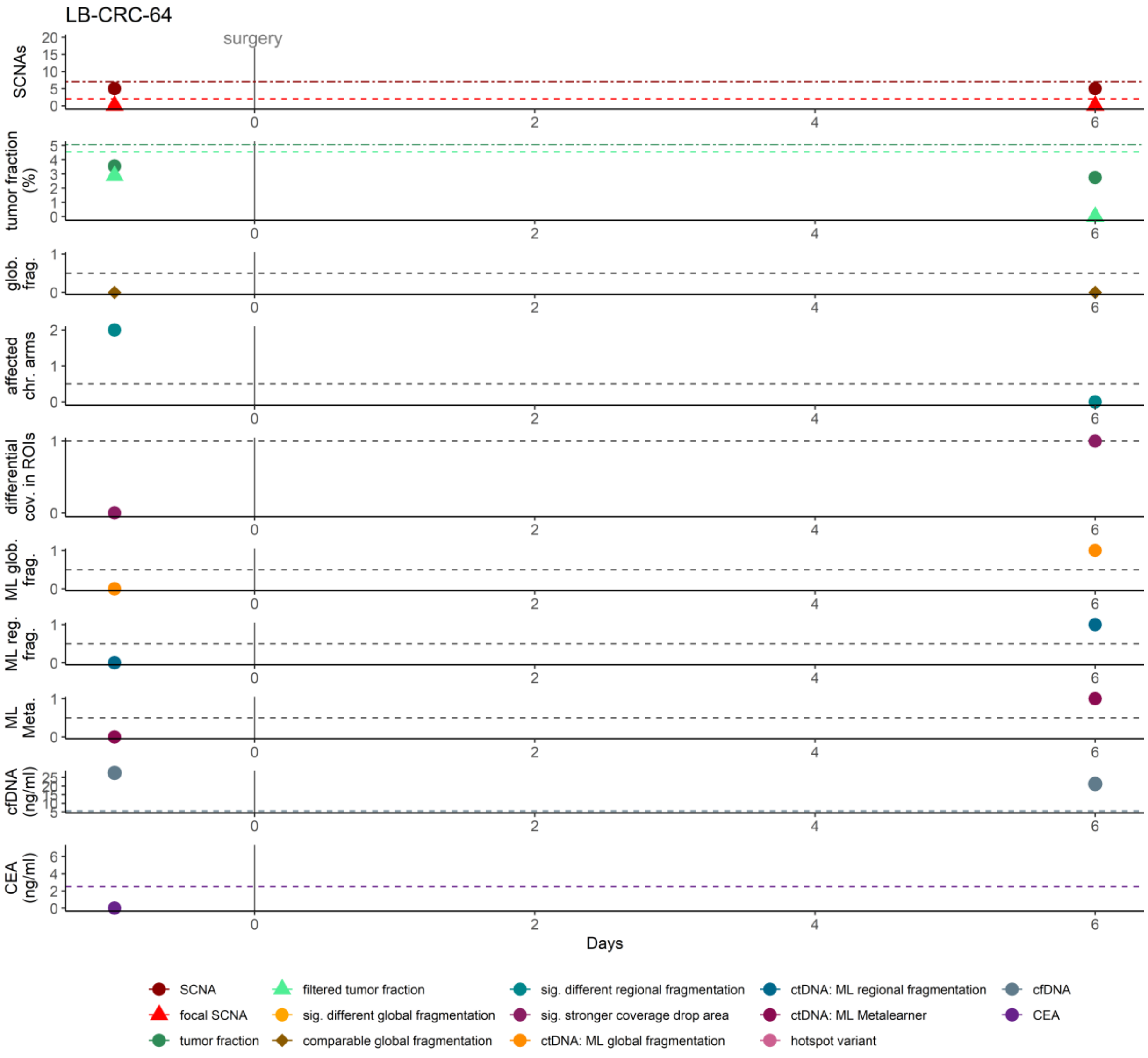

Supplementary Figure 51 Residual disease detection in patient LB-CRC-64.

Patient LB-CRC-64 was diagnosed with stage IIA CRC.

LB-CRC-65

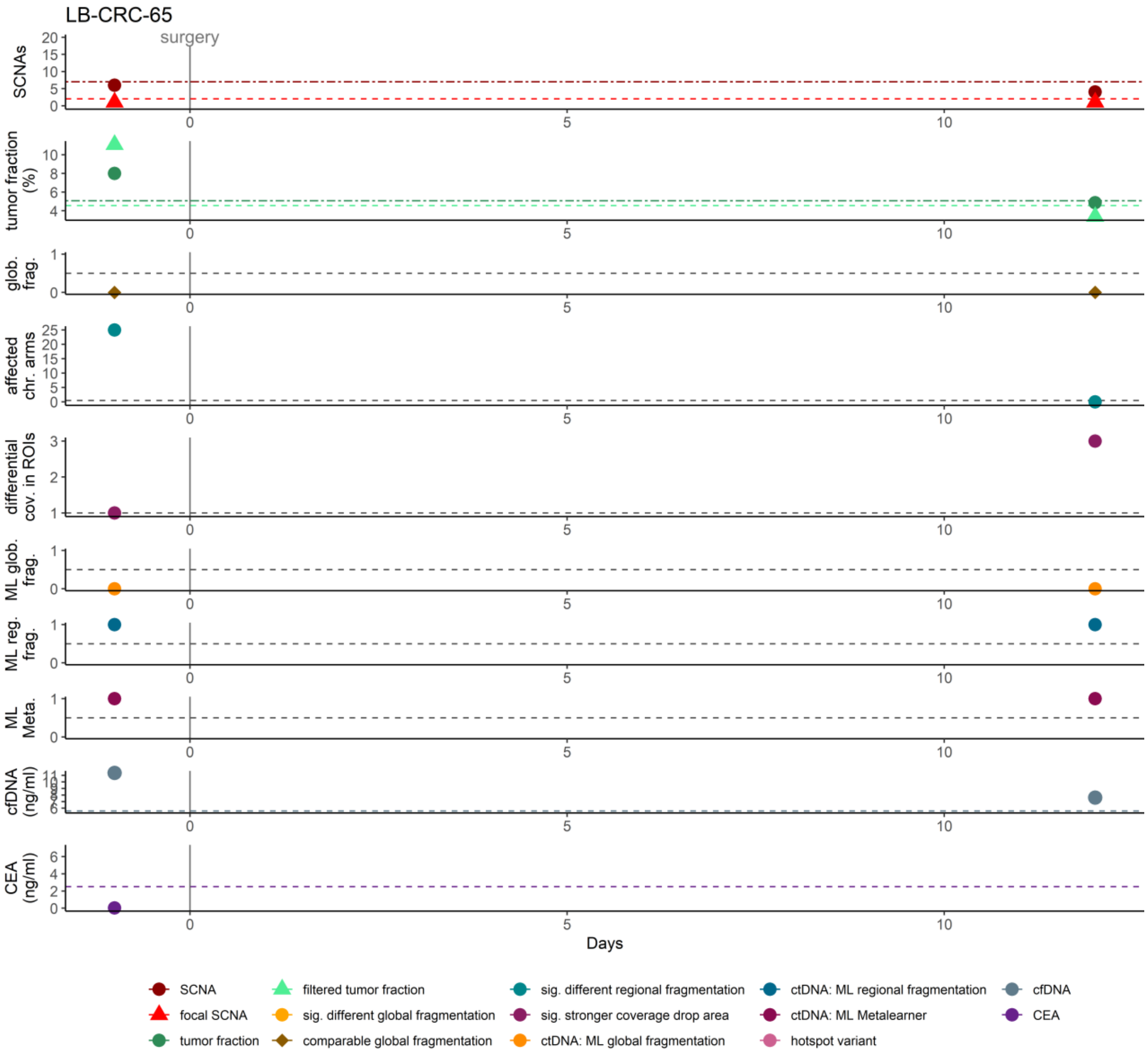

Supplementary Figure 52 Residual disease detection in patient LB-CRC-65.

Patient LB-CRC-65 was diagnosed with stage IIA CRC.

LB-CRC-67

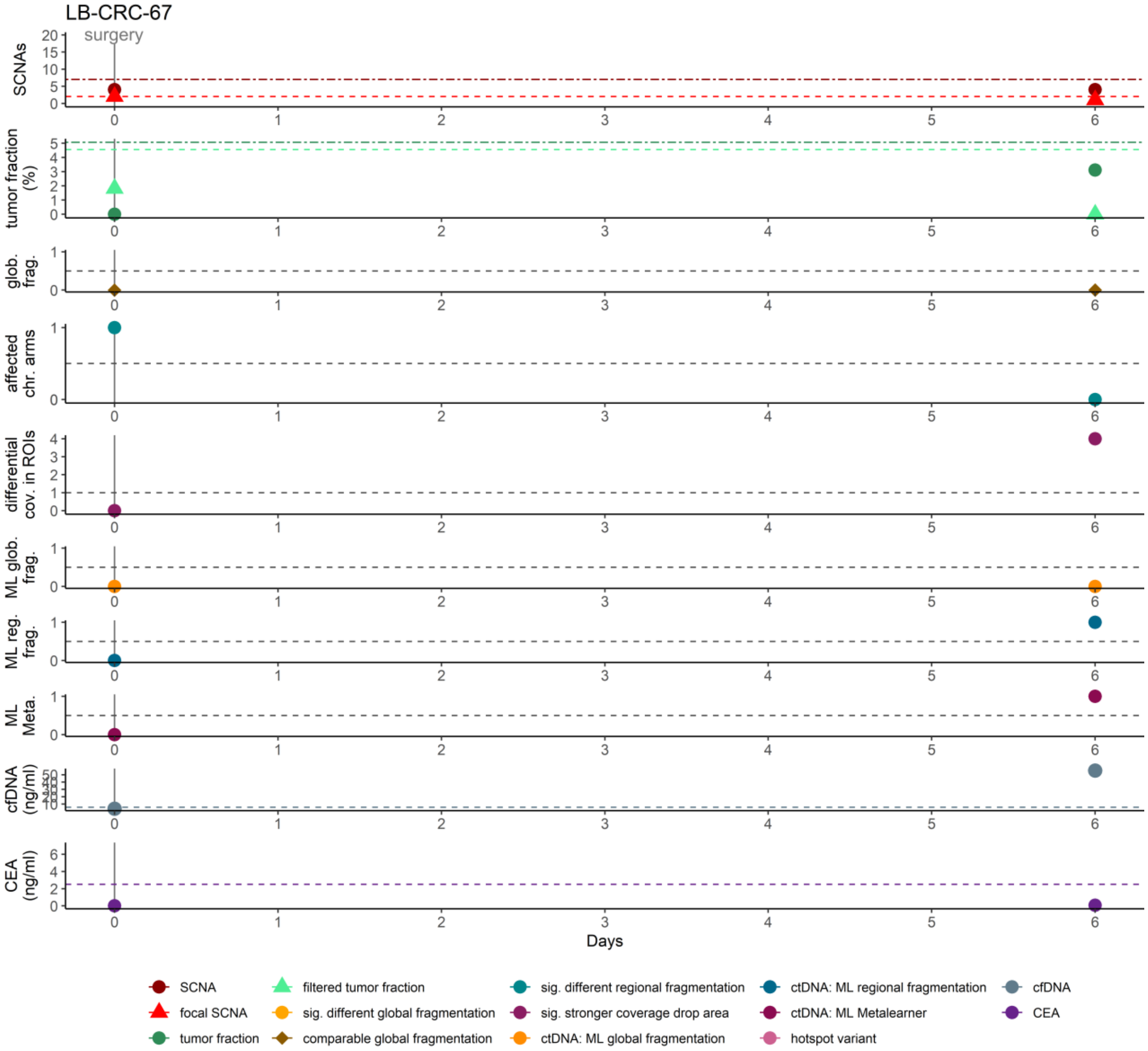

Supplementary Figure 53 Residual disease detection in patient LB-CRC-67.

Patient LB-CRC-67 was diagnosed with stage IIB CRC.

LB-CRC-68

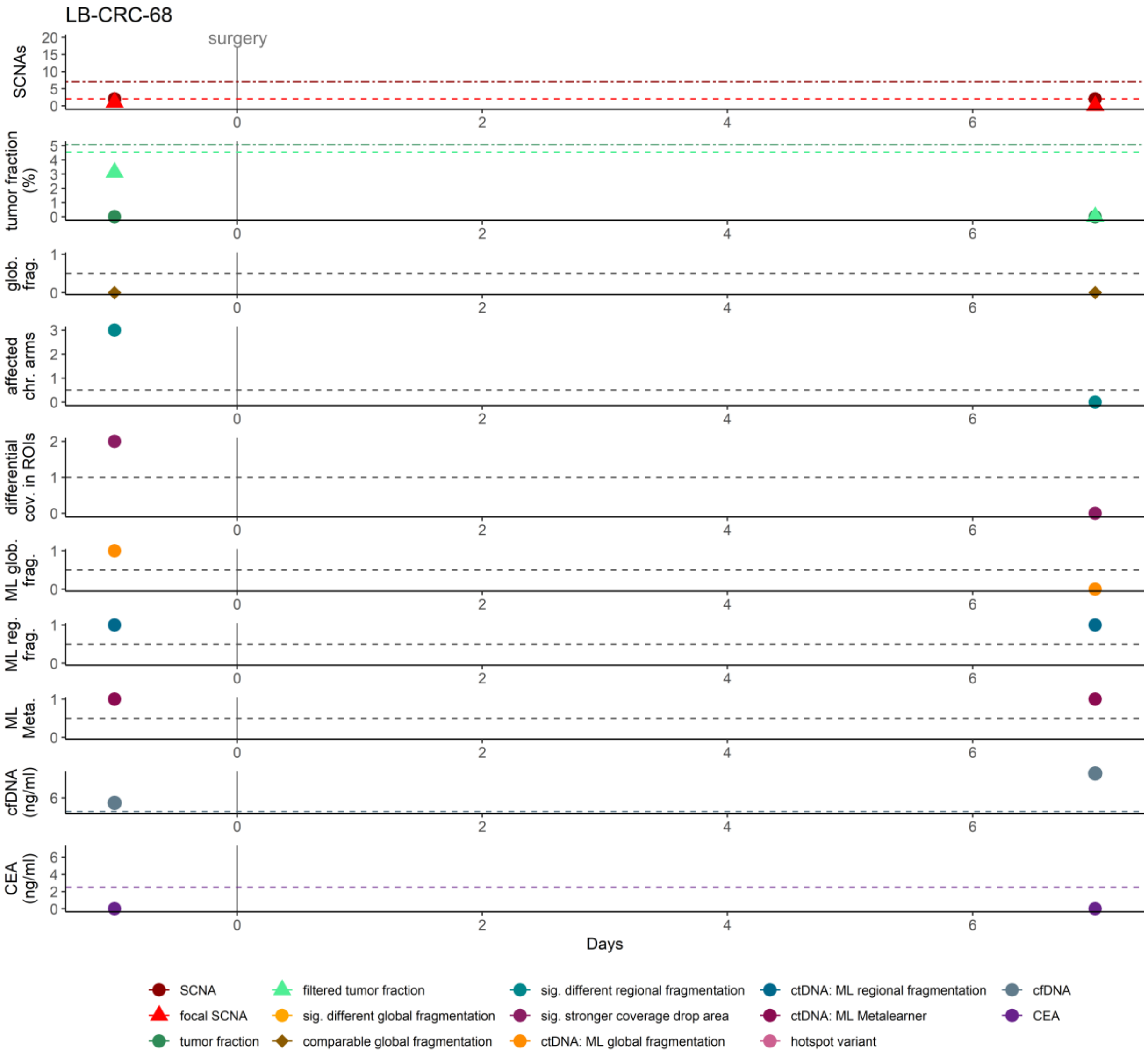

Supplementary Figure 54 Residual disease detection in patient LB-CRC-68.

Patient LB-CRC-68 was diagnosed with stage IIA CRC.

References

1. NCCLS. Protocols for Determination of Limits of Detection and Limits of QuantitationGuideline: Approved Guideline: NCCLS, 940 West Valley Road, Suite 1400, Wayne, Pennsylvania 19087-1898 USA; 2004.
2. Hallermayr A, Benet-Pagès A, Steinke-Lange V, Mansmann U, Rentsch M, Holinski-Feder E, Pickl JMA. Liquid Biopsy Hotspot Variant Assays: Analytical Validation for Application in Residual Disease Detection and Treatment Monitoring. Clin Chem 2021. doi:10.1093/clinchem/hvab124.
3. Adalsteinsson VA, Ha G, Freeman SS, Choudhury AD, Stover DG, Parsons HA, et al. Scalable whole-exome sequencing of cell-free DNA reveals high concordance with metastatic tumors. Nat Commun. 2017;8:1324. doi:10.1038/s41467-017-00965-y.
4. Lai D, Ha G. HMMcopy: A package for bias-free copy number estimation and robust CNA detection in tumour samples from WGS HTS data; 2021.
5. Peneder P, Stütz AM, Surdez D, Krumbholz M, Semper S, Chicard M, et al. Multimodal analysis of cell-free DNA whole-genome sequencing for pediatric cancers with low mutational burden. Nat Commun. 2021;12:3230. doi:10.1038/s41467-021-23445-w.
6. Mouliere F, Chandrananda, Dineika, Piskorz, Anna M., Moore EK, Morris J, Ahlborn LB, Mair R, et al. Enhanced detection of circulating tumor DNA by fragment size analysis. Sci Transl Med. 2018;10.
7. Cristiano S, Leal A, Phallen J, Fiksel J, Adleff V, Bruhm DC, et al. Genome-wide cell-free DNA fragmentation in patients with cancer. Nature. 2019;570:385–9. doi:10.1038/s41586-019-1272-6.
8. COSMIC - Catalogue of somatic mutations in cancer. Cancer Gene Census. https://cancer.sanger.ac.uk/census. Accessed 1 Oct 2021.
9. The Cancer Genome Atlas Network. Comprehensive molecular characterization of human colon and rectal cancer. Nature. 2012;487:330–7. doi:10.1038/nature11252.
